# Liver and Adipose Expression Associated SNPs Are Enriched for Association to Type 2 Diabetes

**DOI:** 10.1371/journal.pgen.1000932

**Published:** 2010-05-06

**Authors:** Hua Zhong, John Beaulaurier, Pek Yee Lum, Cliona Molony, Xia Yang, Douglas J. MacNeil, Drew T. Weingarth, Bin Zhang, Danielle Greenawalt, Radu Dobrin, Ke Hao, Sangsoon Woo, Christine Fabre-Suver, Su Qian, Michael R. Tota, Mark P. Keller, Christina M. Kendziorski, Brian S. Yandell, Victor Castro, Alan D. Attie, Lee M. Kaplan, Eric E. Schadt

**Affiliations:** 1 Department of Genetics, Rosetta Inpharmatics, Seattle, Washington, United States of America; 2 Department of Metabolic Disorders, Merck and Co., Rahway, New Jersey, United States of America; 3 Department of Biostatistics, University of Washington, Seattle, Washington, United States of America; 4 Department of Biochemistry, University of Wisconsin, Madison, Wisconsin, United States of America; 5 Department of Biostatistics and Medical Informatics, University of Wisconsin, Madison, Wisconsin, United States of America; 6 Department of Statistics, University of Wisconsin, Madison, Wisconsin, United States of America; 7 Massachusetts General Hospital Weight Center, Boston, Massachusetts, United States of America; 8 Department of Medicine, Harvard Medical School, Boston, Massachusetts, United States of America; 9 Department of Integrative and Systems Biology, Sage Bionetworks, Seattle, Washington, United States of America; 10 Pacific Biosciences, Menlo Park, California, United States of America; North Carolina State University, United States of America

## Abstract

Genome-wide association studies (GWAS) have demonstrated the ability to identify the strongest causal common variants in complex human diseases. However, to date, the massive data generated from GWAS have not been maximally explored to identify true associations that fail to meet the stringent level of association required to achieve genome-wide significance. Genetics of gene expression (GGE) studies have shown promise towards identifying DNA variations associated with disease and providing a path to functionally characterize findings from GWAS. Here, we present the first empiric study to systematically characterize the set of single nucleotide polymorphisms associated with expression (eSNPs) in liver, subcutaneous fat, and omental fat tissues, demonstrating these eSNPs are significantly more enriched for SNPs that associate with type 2 diabetes (T2D) in three large-scale GWAS than a matched set of randomly selected SNPs. This enrichment for T2D association increases as we restrict to eSNPs that correspond to genes comprising gene networks constructed from adipose gene expression data isolated from a mouse population segregating a T2D phenotype. Finally, by restricting to eSNPs corresponding to genes comprising an adipose subnetwork strongly predicted as causal for T2D, we dramatically increased the enrichment for SNPs associated with T2D and were able to identify a functionally related set of diabetes susceptibility genes. We identified and validated malic enzyme 1 (*Me1*) as a key regulator of this T2D subnetwork in mouse and provided support for the association of this gene to T2D in humans. This integration of eSNPs and networks provides a novel approach to identify disease susceptibility networks rather than the single SNPs or genes traditionally identified through GWAS, thereby extracting additional value from the wealth of data currently being generated by GWAS.

## Author Summary

Genome-wide association studies (GWAS) seek to identify loci in which changes in DNA are correlated with disease. However, GWAS do not necessarily lead directly to genes associated with disease, and they do not typically inform the broader context in which disease genes operate, thereby providing limited insights into the mechanisms driving disease. One critical task to providing further insights into GWAS is developing an understanding of the genetics of gene expression (GGE). We present the first empiric study demonstrating that SNPs in human cohorts that associate with gene expression in liver and adipose tissues are enriched for associating with Type 2 Diabetes (T2D) in humans. By filtering “eSNPs” based on causal gene networks defined in an experimental cross population segregating T2D traits, we demonstrate a dramatically increased enrichment of T2D SNPs that enhance our ability to assess T2D risk. We demonstrate the utility of this approach by identifying malic enzyme 1 (ME1) as a novel T2D susceptibility gene in humans and then functionally validating the causal connection between ME1 and T2D in a mouse knockout model for Me1. This approach provides a path to identifying disease susceptibility networks rather than single SNPs or genes traditionally identified through GWAS.

## Introduction

Genome-wide association studies (GWAS) have revolutionized our ability to identify the causal determinants for common human diseases over the past several years, delivering an unprecedented number of DNA loci associated with a diversity of common human diseases like age-related macular degeneration [1], [2], Crohn's disease [3], type 1 diabetes [3], [4], coronary artery disease [3], [5], HIV-1 infection [6], and type 2 diabetes (T2D) [3], [7]–[10]. One interesting characteristic of single nucleotide polymorphisms (SNPs) identified as associated with disease in these studies is that the great majority do not affect the coding sequence of genes, most often falling in introns or intergenic regions [11]. As a result, GWAS do not necessarily lead directly to the gene or genes in a given locus associated with disease, they do not typically inform the broader context in which the disease genes operate, and even in cases where the susceptibility gene is identified, GWAS do not usually indicate whether you would knock the gene down or activate it in order to treat the corresponding disease. Therefore, GWAS on their own provide limited insights into the mechanisms driving disease [12]–[14]. In addition, the amount of genetic variation explained by GWAS for a given disease is most often significantly less than the heritabilities estimated for the disease. For example, a number of studies estimate the genetic heritability for T2D to be as high as 40%, but the 18 DNA loci identified for T2D to date account for only ∼3% of the variation in T2D [10]. This raises the question of whether there are many more common DNA variants with smaller effects that are not being identified in the GWAS due to lack of power, whether there are many more rare variants with stronger effects that explain the missing variation, or some combination of the two [11], [15].

In fact, in the span of just a few short years in which large-scale GWAS have been carried out, the realization that tractable drug targets and clinically useful biomarkers of disease are not immediately falling out of the data, has for some reduced the enthusiasm for the GWAS approach, intensifying the debate over whether GWAS are the best strategy to elucidate the causes of disease [16]–[18]. Some have attempted to look for enrichments in pre-defined sets of pathways defined by GO, KEGG or other pathway sources and found common variants involved in T2D risk are likely to occur in or near genes in multiple pathways [19]. One clear and immediate task to provide further insights into GWAS is to develop an understanding of the genetics of gene expression (GGE) to facilitate a systems-based understanding of disease. Recently, detailed GGE studies have provided a way to address several of these GWAS limitations [13], [14], [20]–[22]. By mapping the genetic architecture of gene expression in human populations, GGE studies can provide functional support for candidate genes within a given locus. This has been demonstrated a number of times, but most recently in identifying *SORT1*, *PSRC1*, and *CELSR2* as candidate susceptibility genes for heart disease and plasma lipid levels [14], and *ORMDL3* as an asthma susceptibility gene [20], [23]. More generally, GGE studies provide the necessary information to infer causal relationships among genes and between genes and clinical traits, leading to whole gene networks that provide a broader context within which to elucidate the biological function of any given gene with respect to diseases of interest [12]–[14], [24], [25].

**Figure 1:**
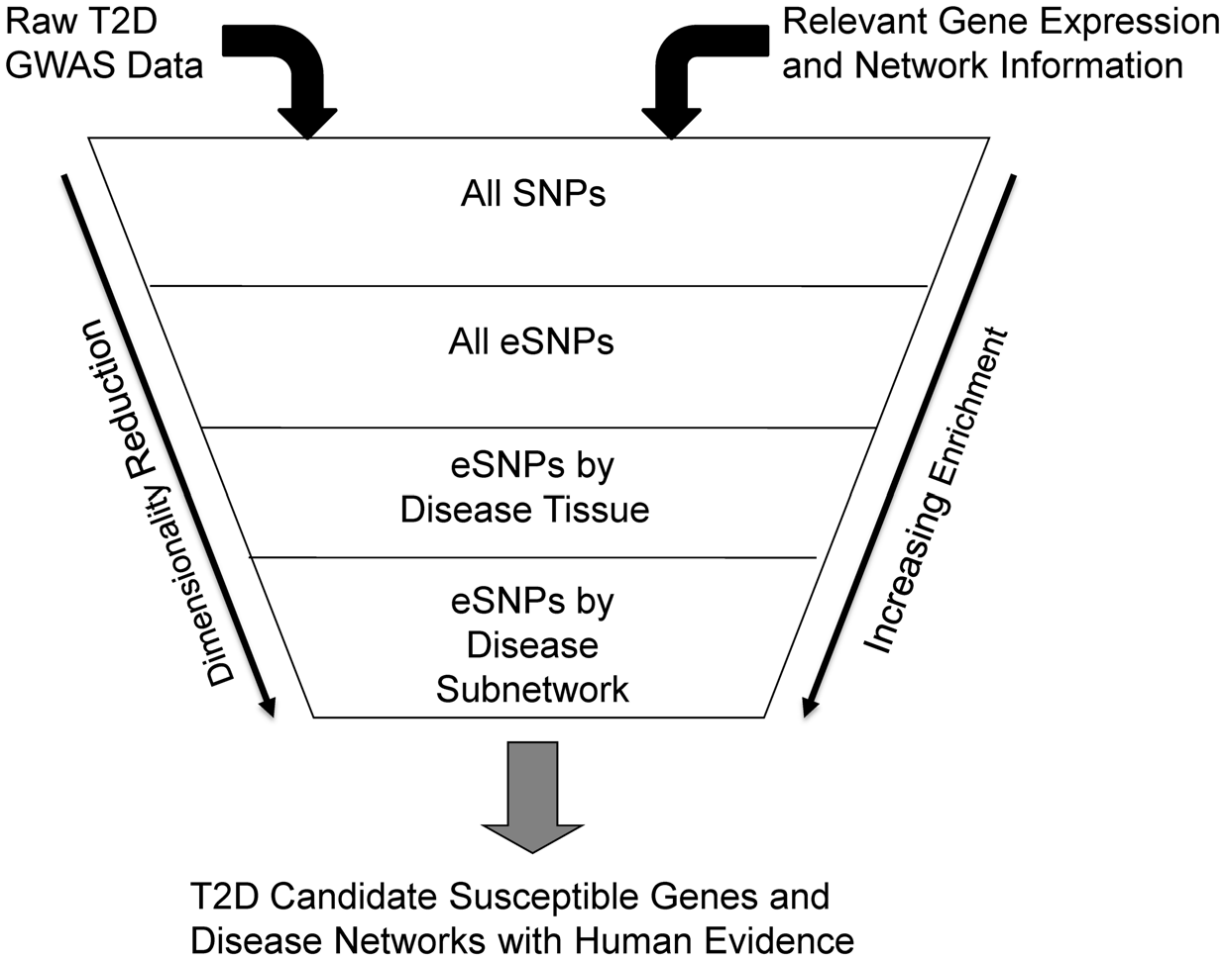
Diagram depicting the process of filtering SNPs using eSNPs and disease associated networks.

One way GGE studies can impact interpretation of GWAS is by providing a way to reduce the dimensionality of the DNA variation space, limiting focus to those DNA variants that have been associated with expression traits and testing whether such SNPs are associated with disease [12]. The set of SNPs associated with expression (eSNPs) in disease-relevant tissues can be considered a functionally relevant subset of all SNPs across the human genome, given they associate with a biologically relevant event (gene expression). However, the extent to which eSNPs inform on disease biology has not been comprehensively characterized for any disease. In this paper, we systematically examined whether eSNPs are more likely to associate with T2D compared to SNPs that a priori have no association to biologically relevant events. We assembled a comprehensive set of eSNPs identified in two GGE study cohorts representing three tissues [12]: liver, subcutaneous fat and omental fat tissues. Given the metabolic relevance of these tissues and the large-scale GWAS undertaken for T2D [26], we tested whether this set of eSNPs was more likely to associate with T2D than randomly selected SNPs. We further constructed a co-expression network from subcutaneous adipose tissue isolated from a mouse population segregating T2D traits and asked whether eSNPs associated with genes comprising these networks and sub-networks were enriched for association with T2D (Figure 1). By comparing the relative enrichments for association to T2D at these increasing levels of granularity, we sought to identify disease-associated subnetworks whose member genes might play important roles in T2D pathogenesis.

## Results

### eSNPs Are Enriched for Association to T2D

We identified eSNPs from two GGE studies: 1) a liver tissue cohort comprised of 427 individuals [12], and 2) a cohort comprised of ∼900 individuals from which liver, subcutaneous and omental adipose tissues were collected from each individual. The number of eSNPs from each tissue and the corresponding cohort sample sizes are summarized in Table S1. To test whether the eSNPs were enriched for association to T2D, we assembled GWAS results from three previously published T2D studies: 1) the Wellcome Trust Case Control Cohort (WTCCC) [3], 2) the Diabetes Genetics Initiative (DGI) [7], and 3) the Diabetes Genetics Replication And Meta-analysis (DIAGRAM) Consortium [10], which combines the results from WTCCC, DGI, and Finland–United States Investigation of NIDDM Genetics (FUSION) [8].

To assess whether these distributions were enriched for SNPs associated with T2D, we empirically estimated the null distribution by randomly sampling 100,000 sets of SNPs from a set of SNPs genotyped in each study (chosen from the full set of SNPs in each study) such that the SNP set size, the location distribution of the SNPs with respect to protein coding genes, and the minor allele frequency (MAF) distribution were similar to that of the eSNP set.

**Figure 2:**
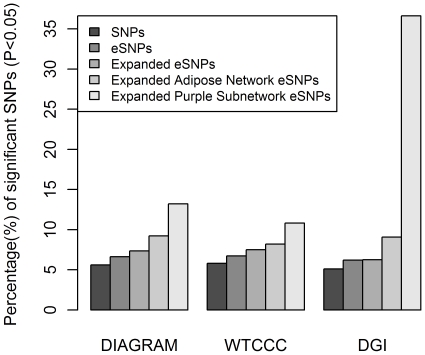
eSNP sets enriched for T2D associated SNPs in three GWAS. The Y axis shows the proportion of SNPs with P_T2D_< = 0.05. The P_T2D_ are from DIAGRAM, WTCCC, and DGI from left to right. In each GWAS cohort, from left to right, the 1^st^ bar shows the observed proportion of all studied SNPs; the 2^nd^ bar shows the proportion of all eSNPs, the 3^rd^ bar shows the proportion of the expanded eSNPs; the 4^th^ bar shows the proportion of the expanded adipose network eSNPs; and the 5^th^ bar shows the proportion of the expanded T2D adipose causal subnetwork eSNPs. In DIAGRAM study, the second bar is significantly higher than the first (p = 2.05×10^−9^), the third bar is higher than the second (p = 1.33×10^−9^), the fourth is higher than the third (p<10^−16^), and the fifth is higher than the fourth (p = 2.97×10^−4^). In WTCCC, the second bar is higher than the first (p = 1.09×10^−2^), the third is higher than the second (p = 1.22×10^−6^), but the fourth and fifth bars are not significantly higher than the third (p = 0.35) or fourth (p = 0.15), respectively. In the DGI study, the second bar is higher than the first (p = 8.0×10^−4^), the third is higher than the second, (p = 2.19×10^−7^), the fourth is higher than the third (p = 1.10×10^−12^), and the fifth is higher than the fourth (p<10^−16^).

The distribution of T2D eSNP association p values from the GWAS (referred to here as P_T2D_) differed significantly from the null distribution in that the eSNP P_T2D_ values were skewed towards the significance end of the P_T2D_ spectrum. For example, in the DGI study, 6.2% of the eSNPs (241 out of 3,888 total) had P_T2D_<0.05, compared to a mean of 5.2% (202 out of 3,888; 95% confidence interval (CI): 4.6% to 5.8%) over the 100,000 randomly generated matched sets (Z = 3.16; p = 8.00×10^−4^, Table 1, Figure 2), representing a 1.19 fold enrichment for SNPs in the eSNP set over the random sets. In addition to testing for enrichments of eSNPs with P_T2D_<0.05, we compared the overall average P_T2D_ of the eSNP set to randomly selected SNP sets matched to the eSNP set with respect to location and MAF. The results were similar to the enrichment observed for eSNPs with P_T2D_<0.05 (Figure S1).

**Table 1:** eSNP Set Enrichment Summary for the DIAGRAM, DGI and WTCCC GWAS.

		*DIAGRAM*	*DGI*	*WTCCC*
	Number Genotyped	16,801	3,888	4,047
All eSNPs	% P_T2D_<0.05 (%)	6.63	6.20	6.72
	% P_T2D_<0.05 from random set (95% CI)	5.70 (5.40, 6.00)	5.19 (4.56, 5.82)	5.94 (5.27, 6.60)
	% P_T2D_<0.05 Fold Increase over random SNP sets(p)	1.16 (2.05×10^−9^)	1.19 (8.00×10^−4^)	1.13 (1.09×10^−2^)
	Number Genotyped	144,660	24,220	25,591
All expanded eSNPs	% P_T2D_<0.05 (%)	7.34	6.26	7.50
	% P_T2D_<0.05 from random set (95% CI)	6.12 (5.72, 6.52)	5.28 (4.90, 5.66)	6.43 (5.99, 6.87)
	% P_T2D_<0.05 Fold Increase over random expanded SNP sets (p)	1.20 (1.33×10^−9^)	1.19 (2.19×10^−7^)	1.17 (1.22×10^−6^)
	Number Genotyped	19,853	3,342	3,539
Adipose network expanded eSNPs	% P_T2D_<0.05 (%)	9.21	9.07	8.20
	% P_T2D_<0.05 from random expanded eSNP set (95% CI)	7.30 (6.93, 7.65)	6.13 (5.31, 6.95)	7.40 (6.53, 8.26)
	% P_T2D_<0.05 Fold Increase over random expanded eSNP sets (p)	1.26 (<10^−16^)	1.48 (1.10×10^−12^)	1.11 (3.49×10^−1^)
	Number Genotyped	628	101	111
Adipose purple subnetwork expanded eSNPs	% P_T2D_<0.05 (%)	13.22	36.63	10.81
	% P_T2D_<0.05 from random adipose network expanded eSNP set (95% CI)	9.21 (6.94, 11.50)	9.04 (3.44, 14.63)	8.20 (3.08, 13.29)
	% P_T2D_<0.05 Fold Increase over random adipose network expanded eSNP sets (p)	1.44 (2.97×10^−4^)	4.05 (<10^−16^)	1.32 (1.57×10^−1^)

Because different SNP panels were used in the different GGE and GWA studies, many of the eSNPs were not genotyped in any of the T2D GWAS. Therefore, we recomputed the P_T2D_ distributions based on all SNPs in strong linkage disequilibrium (LD) with the eSNPs. A SNP was considered in strong LD with an eSNP if the correlation between the two SNPs was >0.89. These SNPs were considered to be representative of our eSNPs and were included in the analysis set (referred to here as the expanded eSNP set) in order to extract the most information from the GWAS data. We again tested whether this expanded eSNP set was enriched for SNPs associated with T2D by empirically estimating the null distribution. For example, in the DGI study, 1,516 SNPs in the expanded eSNP set of 24,220 SNPs (6.3%) had P_T2D_<0.05, compared to an average of 1,279 SNPs (5.3%; [95% CI: 4.9% to 5.7%]) in the random sets (Z = 5.05; p = 2.19×10^−7^), representing a 1.19-fold enrichment for SNPs in the expanded set over the random sets. Similar enrichments were observed in the DGI and WTCCC studies (Table 1, Figure 2).

### Adipose Gene Network Enhances eSNP Association with T2D

While the eSNP P_T2D_ enrichments in liver, omental and subcutaneous tissue were statistically significant, the enrichment was modest (1.19 fold enrichment for the expanded eSNP set). One explanation for this could be that these enrichments were calculated using an eSNP set spanning three distinct tissues without considering how the expression traits relate to networks associated with disease. Therefore, even though the eSNPs considered herein were derived from metabolically active tissues, we considered the possibility that restricting attention to eSNPs corresponding to expression traits in T2D-relevant tissues that are most variable in populations segregating T2D traits may enhance the enrichment for eSNPs associated with T2D.

Towards this end, we tested whether eSNPs corresponding to genes comprising an adipose tissue gene network constructed from an F_2_ intercross between C57BL/6 *ob/ob* and BTBR *ob/ob* mice (referred to here as the B6×BTBR cross) were enriched for association with T2D. The B6×BTBR cross has been previously established as a model population for T2D [27]. While the C57BL/6 *ob/ob* strain becomes obese and develops moderate hyperglycemia, it is compensated by hyperinsulinemia, preventing beta-cell failure and the development of a T2D phenotype. In contrast, the BTBR *ob/ob* strain develops obesity, accompanied by severe hyperglycemia and insulin resistance, ultimately resulting in beta-cell failure and a severe T2D phenotype. Therefore, the gene networks in T2D-relevant tissues in the B6×BTBR mice have the potential to provide insight into pathways and regulatory networks in obesity-induced diabetes [28], [29]. In this setting, we define a gene network as a graphical model comprised of nodes and edges, where the nodes represent gene expression traits or clinical traits, and the edges represent significant, weighted correlations between the corresponding two nodes (expression traits) [30]. Because gene expression, DNA variations and T2D traits were all scored in B6×BTBR cross, there is the potential to identify tissue-specific subnetworks that are causally associated with T2D traits, given DNA variations can be treated as a perturbation on the gene expression and clinical traits, thereby enabling the edges in the network to be directed [12], [24], [31]–[33]. Of the 39,600 genes represented on the microarray used in this study, the upper 25 percent of the most differentially expressed genes were used as input to construct the coexpression network [30]. We then restricted our eSNP set to those omental adipose eSNPs corresponding to genes in the adipose network that mapped to human orthologs (referred to as the adipose eSNP set) and found the expanded eSNP set significantly more enriched for T2D associated SNPs compared to randomly selected eSNPs in the DIAGRAM and DGI studies. In the DGI study, of the 3,342 expanded eSNPs from the adipose set considered, 303 (9.07%) were associated with T2D at the 0.05 significance level, compared to a mean of 6.2% [95% CI: 5.31% to 6.95%] in random expanded eSNP sets (Z = 7.02; p = 1.10×10^−12^). In the DIAGRAM study, 9.2% were associated with T2D at the 0.05 significance level, compared to a mean of 7.3% [95% CI: 6.93% to 7.65%] in random expanded eSNP sets (Z = 10.40; p<10^−16^). However, the adipose eSNP set was not significantly more enriched with small P_T2D_ in the WTCCC study (p = 0.35; Figure 2; Table 1). The lack of significance in the WTCCC cohort was of interest, and given DIAGRAM contains both the DGI and WTCCC cohorts, the intermediate enrichment of DIAGRAM with respect to WTCCC and DGI reflects the strong significance in DGI and lack of significance in WTCCC. It is of particular note that one critical difference between the DGI and WTCCC studies was the matching of DGI cases and controls for BMI, whereas no such matching was done in the WTCCC study. As the adipose network was derived from a mouse cross whose parental strains are both on an *ob/ob* background, the BMI matching in DGI may confer more biological similarities to the cross design and hence better overlap. In addition, while the BMI matching in DGI may enhance power to identify beta-cell loci, rather than loci whose effect on T2D risk was mediated by obesity [34], the BMI matching would not fully account for waist circumference, where those individuals with increased waist circumference compared to individuals with a similar BMI are at increased risk of T2D, where omental adipose tissue is thought to play a role [35].

### Subnetwork Supported as Causal for T2D Further Enhances eSNP Association with T2D

**Figure 3:**
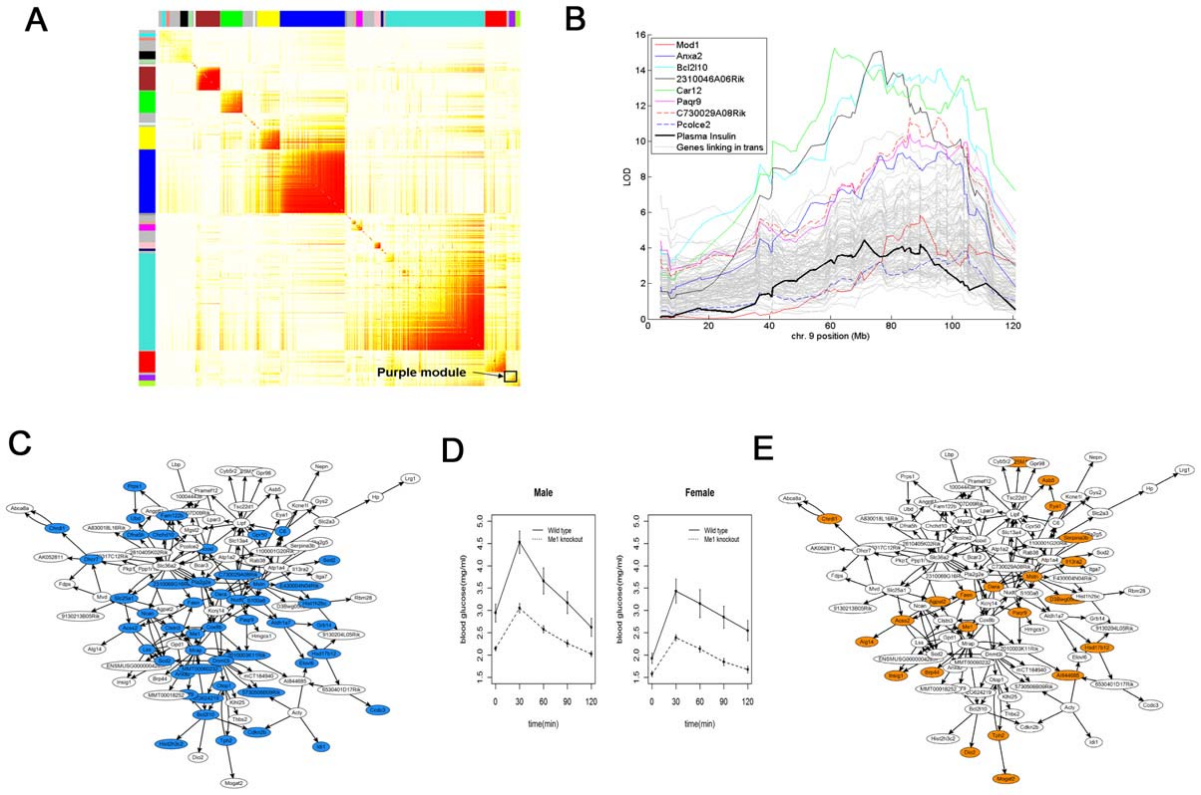
Adipose T2D causal subnetwork and human supporting evidence. A) The adipose coexpression network is comprised of 9,900 gene expression traits. The purple subnetwork comprised of 159 genes is highlighted as the subnetwork most enriched for genes supported as causal for T2D. B) LOD score plots for plasma insulin (solid black), *Me1* adipose expression (solid red), *Anxa2* adipose expression (solid blue), *Bcl2l10* adipose expression (solid cyan), *2310046806Rik* adipose expression (solid black), *Car12* adipose expression (solid green), *Paqr9* adipose expression (solid magenta), *C730029A08Rik* adipose expression (dashed red), *Poclce2* adipose expression (dashed blue), and adipose expression traits linking to this region *in trans* (grey), all measured in the B6×BTBR cross. C) The T2D adipose causal subnetwork is enriched for genes supported as having a causal relationship with plasma insulin levels in the B6×BTBR cross (blue nodes). The white nodes represent genes in the T2D adipose causal subnetwork not supported as causal for insulin traits in the B6×BTBR cross. D) OGTT curves for *Me1−/−* (Male n = 19; Female n = 14) and wild-type control (Male n = 25; Female n = 16) mice (p = 3.16×10^−4^ for male OGTT AUC and p = 1.84×10^−3^ for female OGTT AUC; overall sex adjusted difference p = 7.30×10^−8^). E) The T2D adipose causal subnetwork is enriched for genes in the *Me1−/−* adipose gene expression signature (orange nodes). The white nodes represent genes in the purple subnetwork not in the *Me1−/−* adipose gene signature.

The genes comprising the adipose and islet co-expression networks are not expected to uniformly affect T2D traits [12], [13]. Figure 3A depicts the most highly connected expression traits in the adipose network as a topological overlap map [30]. The adipose network is composed of distinct subnetworks or modules that emerge among the highly interconnected expression traits [36]. Such co-expression subnetworks often contain genes of related biological function [37]. For example, the purple subnetwork in the adipose network was found to be the subnetwork most significantly associated with T2D traits. The genes comprising this subnetwork were enriched for the Panther biological process lipid, fatty acid and steroid metabolism (p = 4.49×10^−8^, Table 2). The first principal component of the gene expression traits making up this subnetwork explained 45.6% of the expression variation of the subnetwork and was strongly positively correlated with several T2D clinical traits measured in the B6×BTBR mice: number of islets (*R* = 0.52, p<1×10^−70^), plasma insulin levels (*R* = 0.70, p<1×10^−70^), and plasma glucose levels (*R* = −0.57, p<3.9×10^−41^).

**Table 2:** Gene sets significantly over-represented in the mouse and human T2D adipose causal subnetwork.

*Gene set type*	*Gene set description*	*Mouse subnetwork (N = 159)*	
		Gene set count^a^	overlap (Enrichment p value^c^, fold enrichment^b^)
Panther biological process	Lipid, fatty acid and steroid metabolism	729	27 (4.95×10^−10^, 4.1)^d^
Causal gene sets	Genes supported as causal for plasma insulin	432	57 (5.26×10^−68^, 29.3)^f^
	Genes supported as causal for plasma glucose	357	44 (1.40×10^−50^, 27.4)^f^
	Genes supported as causal for number of islets	605	47 (1.55×10^−44^, 17.3)^f^
Single gene perturbation experiments	*Me1*−/− versus wild-type signature	2958	32 (9.25×10^−7^, 2.4)^f^

a The number of genes represented on the mouse array that mapped to orthologs in human.

b The overlap count is computed by counting the number of transcripts in the intersection between the indicated gene set and the subnetwork. The fold enrichment is computed as the observed overlap count divided by the expected overlap count, estimated by multiplying the subnetwork gene count by the fraction ‘gene set count divided by total gene count’.

c Nominal p values represent the significance of the Fisher Exact Test statistic under the null hypothesis that the frequency of the indicated gene set is the same between a reference set of all genes represented on the array and the set of genes comprising the subnetwork.

d Total gene count is 17,413.

f Total gene count is 35,345 transcripts on the array.

We next applied a previously described method for inferring causal relationships between the expression traits and T2D traits with respect to genetic loci controlling for the islet count phenotype and plasma glucose and insulin levels [24]. We have previously shown that subnetworks under the control of genetic loci that are also associated with disease traits can be enriched for genes predicted to cause disease trait variation [38]. The purple subnetwork was supported as the most strongly causal subnetwork for the T2D traits in the adipose coexpression network in the B6xBTBR cross (referred to here as the T2D adipose causal subnetwork), with 36% (Fisher Exact Test p = 5.26×10^−68^), 27% (Fisher Exact Test p = 1.40×10^−50^) and 29% (Fisher Exact Test p = 1.55×10^−44^) of the genes in this subnetwork supported as causal for plasma insulin levels, plasma glucose levels, and number of islets, respectively (Figure 3C, Table 2, Table S2). Therefore, while there are many subnetworks identified in the adipose network, they are not all associated with T2D traits, and in the context of the B6xBTBR cross there is a single subnetwork in adipose that is very significantly enriched for genes causally associated with T2D.

Given the strong causal relationship inferred between the T2D adipose causal subnetwork and T2D in the BTBRxB6 cross, we tested whether omental adipose eSNPs corresponding to genes in this subnetwork were more significantly enriched for association to human T2D compared to the adipose network filtered eSNP sets. Astonishingly, of the 101 SNPs in the expanded eSNP set that were associated with the expression of genes in the T2D adipose causal subnetwork, 37 (37%) corresponded to P_T2D_<0.05 in the DGI study, compared to an average of 9.0% SNPs ([95% CI: 3.44% to 14.63%]) in the matched random SNP sets sampled from the adipose network expanded eSNPs (p<10^−16^), further supporting that this subnetwork is an important network for human T2D, and further supporting that the this causal subnetwork may reflect important molecular states associated with increased omental fat mass and the link of this increased fat mass to T2D. Similar enrichments were observed in the DIAGRAM study, although as in the case of the adipose network, the WTCCC enrichment was not significant (Table 1, Figure 2).

### Tissue Specific eSNP Set Enrichment

In addition to the dramatic enrichments observed in restricting attention to those human omental eSNPs corresponding to genes in the B6xBTBR T2D adipose causal subnetwork, the eSNPs generated from the other tissues and from all tissues combined were also enriched for lower P_T2D_ values. The increasing enrichment trend was consistently observed from all tissue-GWAS combinations (Figure S2). While the enrichment magnitude and significance levels were somewhat tissue dependent, there were no profound differences among liver, omental fat and subcutaneous fat tissue eSNPs, possibly reflecting that all three tissues are metabolically active and important in obesity and diabetes.

When comparing eSNPs identified in independent tissues from the same cohort, 72% of the cis eSNPs identified in liver, 79% of those found in omental adipose, and 81% from subcutaneous adipose were also found in the other two tissues: 2,189, 2,286 and 1,999 tissue specific eSNPs were identified in liver, omental adipose, and subcutaneous adipose, respectively. This is consistent with previous findings on tissue-specific effects [13], [39]. Because there is reduced numbers of eSNPs represented in the tissue specific sets, there is reduced power overall to detect enrichments. We note that in cases where the WTCCC enrichments were not significant in restricting attention to omental eSNPs, the enrichments were significant when focusing on eSNPs over all tissues combined. By pooling eSNPs from liver and adipose tissue, our main aim was to increase power to detect enrichments by increasing the number of eSNPs. While pooling of eSNPs from the three tissues was a first step in our analysis, restricting attention to the most disease relevant tissue in this case resulted in the most dramatic enrichment, highlighting the importance of the mouse cross in identifying the most causal subnetworks for disease in the most disease relevant tissue corresponding to the disease relevant tissues we had available in the human cohort.

### eSNP Filtering Methods Lead to the Identification of *ME1* as a T2D Susceptibility Gene

While the enrichment of eSNPs associated with genes in the T2D adipose causal subnetwork was encouraging (37% of the eSNPs in this subnetwork associated with T2D at the nominal 0.05 significance level), the effect sizes were all small, providing for very little power to prioritize the list for direct experimental validation based on the human association data alone. Given the number of putative causal genes represented in this module, we could not carry out experimental validation on all of them. Therefore, we integrated the mouse and human data to prioritize the list of T2D susceptibility genes for validation. To identify susceptibility genes for validation, we identified genes in the T2D adipose causal subnetwork that harbored DNA variations in mouse and human that associated with its expression levels and that were supported as causal for T2D [24]. This is a natural filter to apply, given DNA variations that directly affect the activity of a gene in multiple species, and that in turn are supported as causing variations in disease traits [24], strongly implicate such genes as affecting disease susceptibility [14]. Specifically, for validation purposes, we focused on genes meeting the following three criteria: 1) adipose expression levels in the B6xBTBR cross were associated with genotypes for markers proximal to the gene of interest (i.e., genes that gave rise to *cis* eQTL); 2) supported as causal for T2D traits using a previously described statistical procedure to infer causal relationships between expression and clinical traits [24]; and 3) gave rise to an adipose *cis*-eSNP in humans that associated with T2D in human GWAS. Application of this filter identifies those expression traits in the B6xBTBR cross and human GGE cohorts that are perturbed by *cis* DNA variation, and that in turn associate with T2D traits, directly supporting the genes as causal for T2D in the B6xBTBR cross and the human population.

Of the 159 expression traits in the T2D adipose causal subnetwork, 117 gave rise to cis or trans expression QTL (eQTL) in a distinct region on chromosome 9 (from 65Mb to 95Mb of the chromosome). However, only 8 of these genes were identified with strong adipose *cis*-eQTLs (i.e., the structural genes were located within the chromosome 9 linkage region). Further, 5 of these 8 genes (*Anxa2*, *Bcl2l10*, *C730029A08Rik*, *Me1*, *Paqr9*) were supported by the mouse data as causal for T2D traits (Figure 3B). Among these, only human *ME1* adipose expression was associated with at least one *cis*-eSNP that was also nominally associated with T2D in the DIAGRAM study (P_T2D_ = 0.002) (Figure S3). Therefore, while *Me1* was supported as causal in the B6xBTBR cross, it was one of hundreds of genes supported as causal for T2D traits, but then the only gene of those hundreds whose expression in humans associated with a SNP that also associated with human T2D.

The role of *Me1* in obesity [40]–[42], energy homeostasis [43] and diabetes [44] has been well documented in the literature. Encoding a cytosolic NADP(+)-dependent enzyme involved in the formation of pyruvate from malate, it produces NADPH to supply reducing equivalents for lipogenesis, thus siphoning the reducing equivalents originally derived from glycolysis as NADH to NADPH for fatty acid synthesis [45]. *Me1* is co-regulated together with fatty acid synthetic enzymes by *Chrebp* and *Srebp-1c* and is therefore described as a lipogenic enzyme. Further, we recently provided direct experimental support for the involvement of *Me1* in obesity-related phenotypic characteristics and in gene networks associated with obesity using a *Me1* knockout (*Me1*−/−) mouse model [31].

Here, we extended the validation experiment to T2D related traits. As shown in Table 3, the *Me1*−/− mice fed a high fat diet (HFD) demonstrate significantly lower insulin levels compared to the controls (p = 1.23×10^−9^), thus validating our prediction. In addition, the *Me1*−/− mice showed lower serum glucose levels (p = 3.30×10^−6^) and an improved glucose tolerance at week 23 (Figure 3D), with a 29.5% decrease in the area under the oral glucose tolerance test (OGTT) curve (AUC) relative to wild-type mice (p = 7.30×10^−8^). All of these lines of evidence support a diabetes-resistant phenotype in *Me1*−/− mice. Furthermore, the *Me1*−/− mice also possessed significantly improved lipid profiles including lower total cholesterol (p = 2.19×10^−3^) and triglyceride (p = 1.40×10^−7^) levels. Consistent with the lower body fat reported earlier [31], the serum leptin levels were also significantly lower in the *Me1*−/− mice than in the controls. Therefore, the *Me1*−/− mice appeared to be resistant to both diabetes and obesity development.

**Table 3:** Comparison of metabolic traits between Me1−/− mice and wild-type controls.

	*Male*			*Female*			
*Trait*	Wild type^a^ (n = 25)	*Me1*−/− (n = 19)	Percentage change (%)	Wild type (n = 16)	*Me1*−/− (n = 14)	Percentage change (%)	Difference p value^b^
OGTT AUC (min (mg·ml^−1^))	424.5(117.4)	299.4(36.9)	−29.5	350.2(116.3)	239.8(23.8)	−31.5	7.30×10^−8^
Glucose (mg·ml^−1^)	2.4(0.6)	1.8(0.2)	−24.9	1.9(0.5)	1.6(0.2)	−18.1	3.30×10^−6^
Insulin (mg·ml^−1^)	10.9(6.1)	2.0(1.6)	−81.5	2.9(2.2)	0.6(0.3)	−78.9	1.23×10^−9^
Leptin (mg·ml^−1^)	11.2(3.3)	6.1(2.6)	−45.4	4.6(4.0)	2.4(2.4)	−47.8	1.69×10^−6^
Cholesterol (mg·ml^−1^)	2.2(0.4)	1.9(0.5)	−13.8	1.3(0.3)	1.0(0.1)	−22.7	2.19×10^−3^
Triglycerides (mg·ml^−1^)	2.3(1.0)	1.2(0.4)	−48.6	1.4(0.4)	1.0(0.1)	−26.9	1.40×10^−7^

a Presented as mean (SD).

b All p values reported represent the significance of the t statistic under the null hypothesis that the difference in mean, sex-adjusted values between the *Me1*−/− and wild-type groups is equal to 0.

In order to explore the mechanisms underlying the observed phenotypic changes in the context of the subnetworks identified by the eSNP filtering method, we constructed a single gene perturbation gene expression signature for *Me1*, comprised of 2,958 genes, by identifying adipose genes differentially expressed between wild type and *Me1*−/− male mice. The molecular perturbation signature can serve as an important molecular validation that a putative causal gene underlying a linkage region associated with disease is in fact one of the genes in the linkage region explaining the linkage signal [46]. We found that the *Me1* perturbation signature was significantly enriched for many metabolic pathways, including insulin receptor signaling pathway (p = 2.27×10^−5^), fatty acid (p = 5.49×10^−6^), amine (p = 8.67×10^−8^), lipid (p = 5.47×10^−7^), and monocarboxylic acid metabolic processes (p = 4.73×10^−7^; similar to the purple mouse subnetwork depicted in Figure 3A). The *Me1* perturbation signature was significantly enriched for expression traits in the T2D adipose causal subnetwork: 32 genes overlapped this network whereas only 13 would have been expected by chance, a greater than 2-fold enrichment (Fisher Exact Test p = 2.95×10^−7^; Figure 3E, Table 2). This serves as an important molecular validation of the eSNP filtering method and confirms the causal nature of a gene identified through this approach, *Me1*.

## Discussion

GGE studies provide the necessary information to infer causal relationships among genes and between genes and clinical traits, leading to the construction of gene networks that underlie diseases of interest [12]–[14], [24], [25]. Three fundamental advances presented herein significantly extend this earlier work: 1) to our knowledge, we have demonstrated for the first time that SNPs that associate with human gene expression traits in metabolically relevant tissues are enriched for associating with T2D in multiple T2D studies; 2) the enrichment of eSNPs associating with T2D over randomly selected SNPs dramatically increased as we restricted attention to eSNPs corresponding first to genes comprising the co-expression network from adipose tissue isolated from a mouse population segregating T2D traits, and then to genes comprising a specific adipose subnetwork strongly supported as causal for T2D-associated traits; and 3) we demonstrated directly that causal gene networks provide a path to functionally informing on genetic loci found in GWAS to associate with disease. The inability of GWAS studies to directly elucidate the causal genes and their function with respect to disease is now widely accepted as a problem in search of a solution; we provide one possible solution. Our results taken together support the idea that common forms of disease like T2D are emergent properties of networks that respond to wide-spread variation (genetic and environmental), as opposed to the result of single hits to single genes. The eSNPs for genes in the T2D adipose causal subnetwork that were enriched for associating with T2D were too subtly associated with the disease to be identified in a classic GWAS, due to lack of power. However, the associations were detectable by reducing the number of SNPs tested in a GWAS, given the focus was on those SNPs that associate with the expression of genes in a subnetwork supported as causal for the disease of interest.

The causal reasoning we have used to identify causal relationships between genes and disease traits refers to a statistical inference procedure in which statistical associations between changes in DNA, changes in expression, and changes in complex phenotypes like disease are examined for patterns of statistical dependency among these variables that support directionality among them, where the directionality then provides the source of causal information. This stands in contrast to the classic use of causality in molecular biology or biochemistry, where causality between two proteins implies that one protein has been determined experimentally to physically interact with or to induce processes that directly affect another protein, and that this in turn leads to a phenotypic change of interest. Therefore, experimental validation in this setting is critical. Towards that end, *ME1* was identified as a putative driver of a gene subnetwork containing key regulators of lipogenesis and was then validated in vivo as a gene capable of modulating multiple T2D traits. The genes whose adipose expression levels change in response to knocking out *Me*1 were enriched for genes that 1) fell in this subnetwork, and 2) were supported as causal for T2D in this mouse T2D population. As we have previously detailed, this provides direct experimental support for the gene as a causal regulator of the subnetwork [12], [24], [46]. The T2D adipose causal subnetwork contains several co-expressed genes encoding key lipogenic enzymes, such as fatty acid synthase (*Fasn*), ATP citrate lyase (*Acly*), stearoyl-Coenzyme A desaturase 2 (*Scd2*), lanosterol synthase (*Lss*), farnesyl diphosphate synthetase (*Fdps*), and phospholipase A2, group V (*Pla2g5*). The abnormal liporegulation found in obesity has previously been implicated in the pathogenesis of diabetes [47], [48], especially around the deleterious effects of the elevated levels of triglycerides in peripheral tissues, referred to as “lipotoxicity”. Excess circulating fatty acids present during obesity can accumulate in skeletal muscle tissues, contributing to insulin resistance [49], [50], [51]. Another organ negatively impacted by lipotoxicity is the pancreatic islets, where elevated fatty acid levels have been shown to contribute to β-cell apoptosis, a process thought to involve the *de novo* formation of ceramide and increased nitric oxide (NO) production, resulting in impaired glucose-stimulated insulin secretion [52], [53], [54].

Due to the key role played by *Me1* in fatty acid synthesis, we hypothesized that a genetic knockout of malic enzyme in mice fed a high-fat diet would severely perturb this pathway. This would in turn lead to a decrease in circulating free fatty acids and triglycerides, a diminished ectopic triglyceride deposition, and consequently an improved insulin sensitivity profile. Indeed, both male and female *Me1*−/− mice exhibited dramatically improved responses to an OGTT (Figure 3D), as well as significantly lower plasma triglyceride levels (Table 3; see Text S1 for further discussion of *Me1* and diabetes).

It is important to note that while an adipose subnetwork strongly supported as causal for diabetes in an experimental mouse population demonstrated increased T2D eSNP enrichment when compared to the adipose network as a whole, only moderate enrichments were observed for all eSNPs and adipose-specific eSNPs. One possible explanation could be the limited coverage of eSNPs. For instance, certain GWAS SNPs may not affect gene expression, rather, they may alter post-transcriptional mechanisms such as mRNA splicing, or protein function. In other words, eSNP selection based on the GGE might have missed classes of important functional GWAS SNPs, and thus caused a loss of power. Additionally, our GGE cohorts may not have been appropriately powered to pick up all relevant eSNPs for T2D. The eSNPs used in this study are primarily from liver and adipose tissues. Although these are relevant tissues for T2D, other key tissues such as islet, muscle, and even brain were not available for eSNP discovery and hence a significant percentage of tissue-specific eSNPs were missing from our analysis. This emphasizes the importance of tissue selection for the success of this type of approach. Since many aspects of disease pathology are confined to certain tissues, the ability for eSNPs to inform on the biology relies on having a tissue-appropriate set of eSNPs. Related to this is our characterization of human gene expression traits in non-T2D individuals, which may have caused us to miss many relevant T2D eSNPs. Our first GGE cohort was a population-based random sample, while the second was an obese cohort, hence neither represents an appropriately powered T2D-specific cohort. Finally, the sample sizes of the GGE cohorts were not powered well enough to pick out the types of modest effects found in large GWAS studies. In our analysis, we pooled the eSNPs from the two cohorts in the three tissues as a starting point, mainly to improve power to observe pathway-specific signals. Many of these caveats associated with limited coverage of eSNPs are being addressed via increased funding for very large GGE studies. Therefore, we think the results realized here provide the beginning lines of evidence that eSNPs may in fact generally be enriched with disease associating SNPs.

The set of eSNPs used in our analyses were identified at a false discovery rate (FDR)<10%. The motivation for selecting what could be considered a high FDR threshold was to increase the number of eSNPs to enhance the power to detect patterns of enrichment, as opposed to limiting attention to only the highest confidence single genes associated with disease. We also consider the possibility that the effective FDR decreases as we apply the filtering process of restricting attention to eSNPs whose associated genes are present in co-expression networks and subnetworks supported as causal for diabetes traits. We therefore suspect that this filtering process enhances the enrichment for T2D association primarily by restricting eSNPs to disease susceptibility gene networks, although a reduced effective FDR may also play a role.

Indeed, while we have singled out a single gene, *Me1*, as playing a causal role in this network, the true value of the currently described eSNP filtering approach is in its ability to identify disease susceptibility networks rather than single SNPs or genes traditionally identified through GWAS. In fact, the knockout gene expression signature for *Me1* was significantly enriched for genes in the T2D adipose causal gene network, providing direct experimental evidence of the high degree of interconnectivity within this network, where perturbing one gene supported as causal for disease affects many other genes supported as causal in this network, as we have previously shown for other disease causal networks [12], [46]. We have shown for the first time that SNPs that are associated with transcript abundance are more likely to associate with a complex trait as well. This type of approach provides a way to reduce the dimensionality of the DNA variation space and can help us reconsider how to map complex disease using gene expression traits. This approach can also help prioritize GWAS findings, for instance, by including the eSNPs corresponding to genes in causal disease networks in testing for epistasis or for consideration in future genetic association studies.

GWAS will continue to deliver high-confidence correlations between DNA changes at a given locus and disease-associated traits of interest. Our understanding of the individual genes at these loci that alter disease susceptibility and the broader context in which they operate can be enhanced by leveraging studies that seek to map the genetics of gene expression. Generating large-scale molecular profiling data sets in both human and experimental segregating populations potentially provides additional power to elucidate not only the genetic basis of disease, but also the impact the genetic basis of disease has on molecular networks that in turn drive physiological states associated with disease. Diabetes pathogenesis involves many pathways operating in different tissues and distinct physiological processes (blunted insulin signaling and failure of beta cells to compensate by producing more insulin). Therefore, the integration of large-scale molecular profiling, genotypic, clinical, and other biologically relevant data will be critical if we hope to understand more fully how genetic and environmental perturbations lead to complex traits like disease. Integration of a diversity of data in this setting will be key, since no single data dimension will provide the complete answer.

## Methods

### eSNP Processing and Analysis

For the liver-specific GGE cohort, more than 39,000 transcripts were profiled and 782,476 unique SNPs were genotyped in more than 400 human liver samples [12]. In this cohort, the genetics of gene expression analysis resulted in the detection of 3,309 unique eSNPs at an FDR<10% [14]. The eSNP processing and analysis were carried out as previous described [14]. All expression data have been deposited in the Gene Expression Omnibus database under accession number (GSE9588) [14].

The second multi-tissue GGE cohort was comprised of patients who underwent RXY gastric bypass surgery. Liver, subcutaneous adipose and omental adipose tissues were collected from each patient at the time of surgery at Massachusetts General Hospital. Genomic DNA was extracted from liver tissue for each patient, and total RNA was extracted from liver, subcutaneous adipose and omental adipose tissues. Each RNA sample was profiled on a custom 44K Agilent array. RNA processing methods are detailed in Text S1. Each DNA sample was genotyped on the Illumina 650Y BeadChip array. We successfully genotyped 950 samples. Identity by state (IBS) analysis was performed to identify related individuals within this cohort. Eighteen parent-offspring, 6 sibling and 8 second degree relatives were identified, and 4 of these were related as trios. Twenty-eight individuals were removed to eliminate IBS in the dataset, leaving 922 samples for use in the analysis. Demographic information including age, race, gender, height, type of surgery and year of surgery were collected for each patient (Text S1). We required that the minor allele frequency for a SNP be >5% in order to be considered in the analyses.

*Cis* and *trans* acting expression quantitative trait loci (eQTLs) were identified using a method similar to that previously described [14]. The *cis* eQTL for a given expression trait were defined as those with corresponding SNPs located within 1 megabase (Mb) of the transcription start or stop of the associated structural gene. All other associations were considered *trans*. SNP associations were identified using the Kruskal-Wallis test. The association p values were adjusted to control for testing of multiple SNPs and expression traits using an empirically determined FDR constrained to be <10%. For *cis* eQTL, we only tested for associations to SNPs that were within 1 Mb of the annotated start or stop site of the corresponding structural gene. The empirical FDR permutations were restricted to SNPs within the *cis* regions. In the case of *trans* eQTL, all SNPs were tested for association to each of the expression traits. Where SNP associations were identified to the same trait in high LD with each other, the SNP with the most significant p value was reported.

When comparing eSNPs identified in independent tissues from the same cohort, 72% of the cis eSNPs identified in liver, 79% of those found in omental adipose and 80.5% from subcutaneous adipose were also found in the other two tissues. Of the eSNPs detected, 2,189, 2,286 and 1,999 were specific eSNPs to liver, omental adipose and subcutaneous adipose, respectively. When compared to the set of liver eSNPs from the first cohort there was a 66% overlap in eSNPs indentified between the two studies. The set of eSNPs used in the paper is the combined set of eSNPs from the four sources, comprising 18,785 unique eSNPs in total.

### Statistical Analysis

#### Statistical methods for evaluating eSNP set enrichment

We used a matched random sampling strategy to assess whether a given set of eSNPs was more likely to associate with T2D than randomly selected sets of SNPs with equal size, similar location to human genes, similar MAF distributions, and similar LD structures. For *cis*-eSNPs, we first required that the random SNP pool be composed of SNPs located within 1MB of human gene regions and with MAF >5%. We then binned the random SNPs into five groups according to their MAF: 5–10%, 10–20%, 20–30%, 30–40% and 40–50%. For each *cis*-eSNP, we randomly selected a genotyped SNP from the same MAF group in each random sample. A similar procedure was employed for *trans*-eSNPs, except that we did not require the random SNP be within 1MB of a gene region. When there were multiple *e*SNPs located in one LD block, we randomly selected the same number of SNPs (matched according to MAF and position with respect to gene region) that were in the LD block corresponding to the matched eSNPs. This process was repeated 100,000 times. For each random SNP set, we counted the percentage of SNPs with GWAS p<0.05, 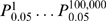, and from this constructed the null distribution. We then compared the observed percentage of eSNPs with GWAS p<0.05 to the null distribution in order to estimate the enrichment p value. An approximation method, which was used to increase the enrichment p value resolution, is detailed in Text S1.

#### Statistical methods for evaluating eSNP expanded set enrichment

We first used the matched random sampling strategy described above to get a random set of SNPs that matched the eSNP set. The eSNP set expanded by including all SNPs in strong LD with the eSNPs. A SNP was considered in strong LD with an eSNP if the correlation between the two SNPs was >0.89. The random SNP set was expanded in a similar fashion by including SNPs that were correlated with a given SNP (*R*>0.89). We required the final size of the expanded random set of SNPs to be within ±10% of the size of the expanded set of eSNPs. This requirement ensured that the expanded eSNP set would not be biased relative to the expanded random SNP set by including more SNPs with a richer LD structure, thereby having a greater probability of associating with T2D and expression traits, even in cases where the expression and T2D are completely genetically independent. By forcing the expanded set sizes to be equal we removed the potential confounding effect caused by LD structure. Therefore, the random sampling scheme produced sets of SNPs in which the LD, set size, and location with respect to protein coding genes, and MAF distributions matched those of the expanded eSNP sets. The process was again repeated 100,000 times. The enrichment p values were then derived as described above.

#### Statistical methods for evaluating an enrichment increase when comparing two sets of eSNPs

When assessing if one set of eSNPs (size = *M*) was more enriched for small P_T2D_ than a second eSNP set (size = *N*, *N*>*M*), we compared between the two classes directly. For each cis (or trans) eSNP in the second set, we randomly selected a cis (or trans) eSNP from the first set such that it fell in the same MAF group as the first. When there were multiple eSNPs located in one LD block in the second set, we randomly selected the same number of eSNPs from the first set from the corresponding LD block. For each random sample we counted the percentage of sampled eSNPs with P_T2D_<0.05. The proportion of times this percentage exceeded the observed percentage of the second eSNP set (with P_T2D_<0.05) was taken as the estimate of the p value under the null hypothesis that the two sets of eSNPs were similarly enriched (vs. the alternative hypothesis that the second eSNP set was more enriched than the first. Comparison of two expanded eSNP sets proceeded in a similar fashion, where again all SNPs that were significantly correlated (*R*>0.89) with any of the randomly identified eSNPs in each random sample were included.

Given the above procedures, when comparing if the adipose network eSNPs were more enriched for low P_T2D_ than all eSNPs, we compared the adipose network eSNPs and equal-sized groups of random eSNPs. When comparing if the T2D adipose causal subnetwork eSNPs were more enriched for low P_T2D_ than the adipose network eSNPs, we compared the subnetwork eSNPs and equal-sized groups of random adipose network eSNPs. This answered directly whether the SNP filtering process increased enrichment for small P_T2D_.

### Generation of the B6×BTBR Cross F_2_ Mice

554 F_2_ mice were generated in a cross between two inbred strains, both containing the *ob* mutation at the leptin locus: C57BL/6 *ob/ob* and BTBR *ob/ob* (referred to as the B6×BTBR cross) [27]. All F_2_ animals were maintained on a chow diet for ten weeks and were clinically characterized with respect to obesity- and diabetes-related traits at four, six, eight and ten week time points. Further details regarding the plasma glucose and insulin measurements, as well as islet isolation procedures, can be found in Keller et al. [29]. At the time of necropsy, gonadal white adipose tissue was collected from 497 mice. RNA was prepared using the same methods as described previously [29] and hybridized to an Agilent custom murine gene expression microarray.

### Reconstruction of the Adipose Coexpression Network

Of the 39,600 transcribed sequences represented on the microarray, the top 25 percent rank ordered by degree of differential expression in the adipose tissue were included in the reconstruction process [29]. These gene expression traits were used to construct weighted, co-expression subnetworks comprised of the most highly connected nodes from each tissue and sex using previously described methods [30] (Text S1). QTL were detected for each of the expression and metabolic traits using a forward stepwise regression procedure [55], [56]. QTL with pleiotropic effects on expression and metabolic traits were identified using a multivariate likelihood test [24], [57]. The QTL, expression, and metabolic trait data were then integrated to assess whether each expression trait was supported as having a causal relationship with each of the metabolic traits, with respect to QTL detected with pleiotropic effects on the expression and metabolic traits [24].

### Construction and Phenotypic Characterization of *Me1*−/− Mice

A naturally occurring mouse mutant deficient in Me1 enzymatic activity was first reported by Lee *et al.* in 1980 [58]. The detailed methods for breeding, genotyping, and characterization of the *Me1^−/−^* mice have been described previously [31], [59]. Littermate male *Me1^−/−^* and wild-type mice were challenged with a high fat diet (HFD) starting at 7–8 weeks of age for 19 weeks. An oral glucose tolerance test (OGTT) was performed at week 23–24 of age and terminal blood serum was collected at week 26–27 of age. For females, HFD was initiated at week 8–10 and continued for 19 weeks. OGTT was performed at week 26–28 of age and terminal serum samples were collected at week 27–28 of age. Mice were euthanized at the end of the HFD period. For OGTT, glucose was administered at 2g/kg of mouse mass via oral gavage, mice were fasted 18 hours Prior, and glucose levels were measured using a OneTouch Ultra glucometer (LifeScan, Inc, Milpitas, CA) at 0, 30, 60, 90, and 120 min. Serum was collected from blood using Becton Dickson (Franklin Lakes, NJ) Microtainer tubes with SST. Insulin and leptin were measured using Millipore's (Billerica, MA) Multiplexed Biomarker Immunoassay for Luminex xMap using a Bio-Rad's (Hercules, CA) Bio-Plex machine. The other serum parameters were measured using a colorimetric assay. Triglycerides were measured at OD 510 nm using reagents from Roche Diagnostics (Indianapolis, IN). Cholesterol was measured using reagents from Stanbio (Boerne, TX) at OD 510 nm as well.

### Identification of Adipose Expression Signature in Me1−/− Mice

The gonadal white adipose tissues were collected from 10 male *Me*1−/− mice and 10 male littermate wild-type (wt) control mice. The detailed methods have been described previously [31], [59]. The adipose tissues were homogenized and total RNA extracted using Trizol reagent (Invitrogen, CA). Three micrograms of total RNA was reverse transcribed and labeled with either Cy3 or Cy5 fluorochrome. Labeled complementary RNA (cRNA) from each animal was hybridized against a pool of labeled cRNAs constructed from equal aliquots of RNA from the control animals using Agilent arrays consisting of 39,556 non-control probes that represent 37,687 genes. Arrays were quantified on the basis of spot intensity relative to background, adjusted for experimental variation between arrays using average intensity over multiple channels, and fitted to a previously described error model to determine significance [60], [61]. Gene expression measures are reported as the ratio of the mean log10 intensity (mlratio). A Student's t-test was used to identify genes with significant differences between *Me1^−/−^* animals and the corresponding wt control mice. These genes were defined as “signature” genes, representing the perturbed gene expression signature as a result of single gene modification. The significance level was set to p<0.05. The false discovery rate, calculated using Q-value [62], at this significance level was 20%.

## Supporting Information

Figure S1 SNP Sets Average log (P_T2D_) from the three GWAS. The Y axis shows the average of −log (P_T2D_). P_T2D_ are from the DIAGRAM, WTCCC, and DGI studies, from left to right. In each GWAS cohort, from left to right, the first bar shows the average of −log (P_T2D_) of all studied SNPs; the second bar shows the average of −log (P_T2D_) of all eSNPs, the third bar shows the average of −log (P_T2D_) of adipose network eSNPs; the fourth bar shows the average of −log (P_T2D_) of T2d adipose causal subnetwork eSNPs. In DIAGRAM, the second bar is higher than the first (P = 1.37×10^−9^), the third is higher than the second P = 1.21×10^−4^), and the fourth is higher than the third (P = 1.22×10^−4^). In the WTCCC study, the second bar is higher than the first (P = 1.01×10^−3^), the third is not statistically distinguishable from the second (P = 0.07), and the fourth is marginally higher than the third (P = 0.04). In the DGI study, the second bar is higher than the first (P = 5.83×10^−3^), the third is higher than the second (P = 8.48×10^−4^), and the fourth is higher than the third (P = 1.17×10^−7^).
(0.03 MB DOC)

Figure S2 Enrichment of tissue-specific eSNP sets for SNPs associated with T2D in three GWAS. The Y axis shows the proportion of SNPs with P_T2D_< = 0.05. From left to right, the tissues liver tissue from liver-specific cohort, Massachusetts General Hospital (MGH) liver tissue, MGH omental adipose, and MGH subqutaneous adipose tissue. For each cluster of bars representing a specific tissue in a specific GWAS, the first bar shows the observed proportion of all studied SNPs, the second bar shows the proportion of all eSNPs, the third bar shows the proportion of adipose network eSNPs, and the fourth bar shows the proportion of T2D adipose causal subnetwork eSNPs with P_T2D_<0.05.
(0.19 MB DOC)

Figure S3 Regional plot of *ME1* gene association with T2D in the DIAGRAM GWAS. For *ME1* gene region on chromosome 6, genotyped and imputed SNPs are plotted with their meta-analysis P_T2D_ values (as −log10 values) as a function of genomic position (NCBI Build 35). SNPs associated with *ME1* adipose expression are shown as red triangles. The estimated recombination rates (taken from HapMap) are plotted to reflect the local LD structure around the associated SNPs and their correlated proxies (Y axis on the right).
(0.04 MB DOC)

Table S1 Tissue-specific eSNP discovery summary.
(0.03 MB DOC)

Table S2 The T2D causal adipose subnetwork (purple module) gene list, gene-trait correlations and causal genes.
(1.82 MB DOC)

Text S1 Supplementary methods and discussion.
(0.18 MB DOC)
