# Supplementary material for: Liver and Adipose Expression Associated SNPs Are Enriched for Association to Type 2 Diabetes
Source: PLoS Genet. 2010 May 6;6(5):e1000932. doi: 10.1371/journal.pgen.1000932 (PMC2865508; doi:10.1371/journal.pgen.1000932)
Supplement: Figure S1 — SNP Sets Average log (PT2D) from the three GWAS. The Y axis shows the average of −log (PT2D). PT2D are from the DIAGRAM, WTCCC, and DGI studies, from left to right. In each GWAS cohort, from left to right, the first bar shows the average of −log (PT2D) of all studied SNPs; the second bar shows the average of −log (PT2D) of all eSNPs, the third bar shows the average of −log (PT2D) of adipose network eSNPs; the fourth bar shows the average of −log (PT2D) of T2d adipose causal subnetwork eSNPs. In DIAGRAM, the second bar is higher than the first (P = 1.37×10^−9^), the third is higher than the second P = 1.21×10^−4^), and the fourth is higher than the third (P = 1.22×10^−4^). In the WTCCC study, the second bar is higher than the first (P = 1.01×10^−3^), the third is not statistically distinguishable from the second (P = 0.07), and the fourth is marginally higher than the third (P = 0.04). In the DGI study, the second bar is higher than the first (P = 5.83×10^−3^), the third is higher than the second (P = 8.48×10^−4^), and the fourth is higher than the third (P = 1.17×10^−7^). (0.03 MB DOC) [file pgen.1000932.s001.doc]

**Figure S1. SNP Sets Average log (P_T2D_) from the three GWAS**. The Y axis shows the average of –log (P_T2D_). P_T2D_ are from the DIAGRAM, WTCCC, and DGI studies, from left to right. In each GWAS cohort, from left to right, the first bar shows the average of –log (P_T2D_) of all studied SNPs; the second bar shows the average of –log (P_T2D_) of all eSNPs, the third bar shows the average of –log (P_T2D_) of adipose network eSNPs; the fourth bar shows the average of –log (P_T2D_) of T2d adipose causal subnetwork eSNPs. In DIAGRAM, the second bar is higher than the first (P=1.37 × 10^-9^), the third is higher than the second P=1.21 × 10^-4^), and the fourth is higher than the third (P=1.22 × 10^-4^). In the WTCCC study, the second bar is higher than the first (P= 1.01 × 10^-3^), the third is not statistically distinguishable from the second (P=0.07), and the fourth is marginally higher than the third (P=0.04). In the DGI study, the second bar is higher than the first (P= 5.83 × 10^-3^), the third is higher than the second (P= 8.48 × 10^-4^), and the fourth is higher than the third (P= 1.17 × 10^-7^).
