# Supplementary material for: Liver and Adipose Expression Associated SNPs Are Enriched for Association to Type 2 Diabetes
Source: PLoS Genet. 2010 May 6;6(5):e1000932. doi: 10.1371/journal.pgen.1000932 (PMC2865508; doi:10.1371/journal.pgen.1000932)
Supplement: Figure S2 — Enrichment of tissue-specific eSNP sets for SNPs associated with T2D in three GWAS. The Y axis shows the proportion of SNPs with PT2D< = 0.05. From left to right, the tissues liver tissue from liver-specific cohort, Massachusetts General Hospital (MGH) liver tissue, MGH omental adipose, and MGH subqutaneous adipose tissue. For each cluster of bars representing a specific tissue in a specific GWAS, the first bar shows the observed proportion of all studied SNPs, the second bar shows the proportion of all eSNPs, the third bar shows the proportion of adipose network eSNPs, and the fourth bar shows the proportion of T2D adipose causal subnetwork eSNPs with PT2D<0.05. (0.19 MB DOC) [file pgen.1000932.s002.doc]

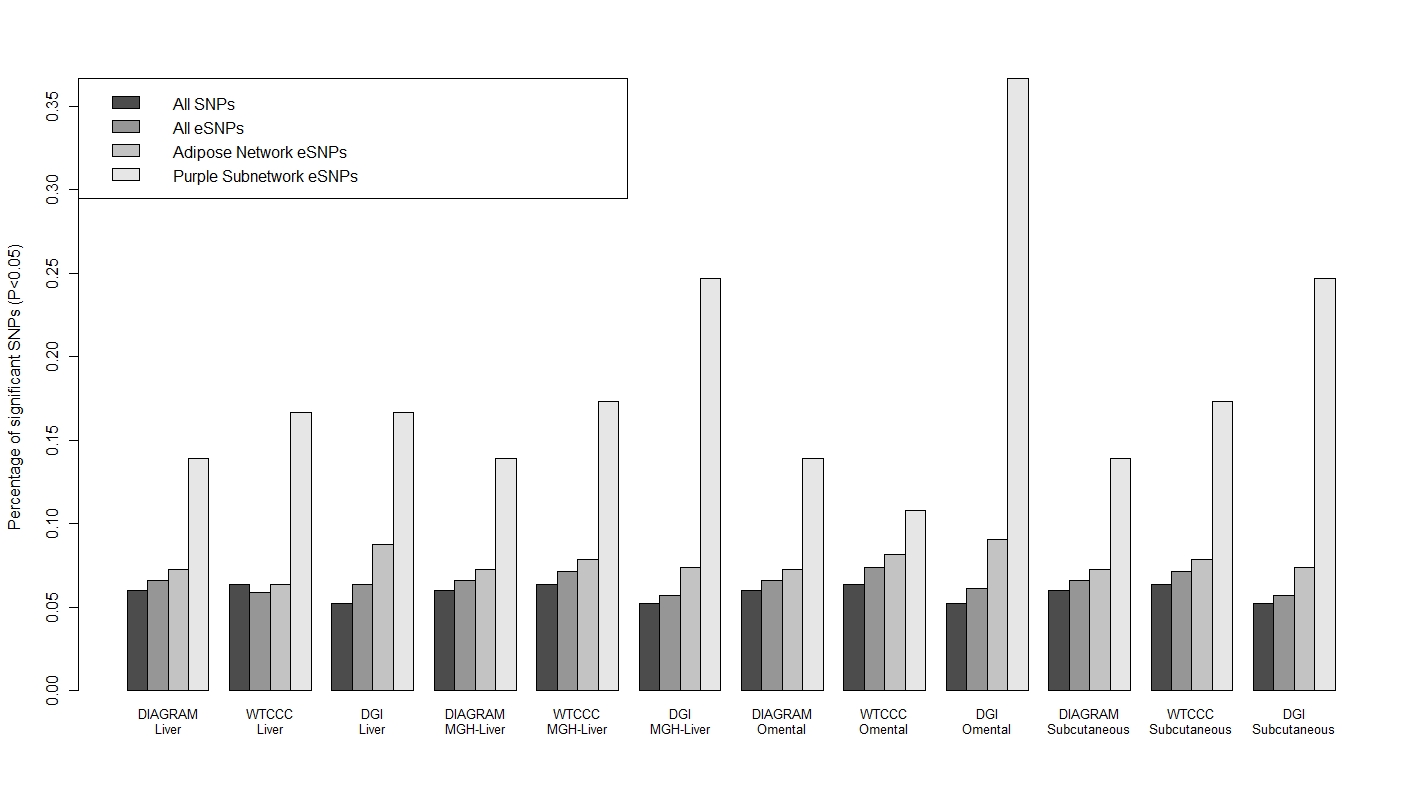


**Figure S2. Enrichment of tissue-specific eSNP sets for SNPs associated with T2D in three GWAS**. The Y axis shows the proportion of SNPs with P_T2D_ <= 0.05. From left to right, the tissues liver tissue from liver-specific cohort, Massachusat General Hospital (MGH) liver tissue, MGH omental adipose, and MGH subqutaneous adipose tissue. For each cluster of bars representing a specific tissue in a specific GWAS, the first bar shows the observed proportion of all studied SNPs, the second bar shows the proportion of all eSNPs, the third bar shows the proportion of adipose network eSNPs, and the fourth bar shows the proportion of T2D adipose causal subnetwork eSNPs with P_T2D_ < 0.05.
