# Supplementary material for: Liver and Adipose Expression Associated SNPs Are Enriched for Association to Type 2 Diabetes
Source: PLoS Genet. 2010 May 6;6(5):e1000932. doi: 10.1371/journal.pgen.1000932 (PMC2865508; doi:10.1371/journal.pgen.1000932)
Supplement: Figure S3 — Regional plot of ME1 gene association with T2D in the DIAGRAM GWAS. For ME1 gene region on chromosome 6, genotyped and imputed SNPs are plotted with their meta-analysis PT2D values (as −log10 values) as a function of genomic position (NCBI Build 35). SNPs associated with ME1 adipose expression are shown as red triangles. The estimated recombination rates (taken from HapMap) are plotted to reflect the local LD structure around the associated SNPs and their correlated proxies (Y axis on the right). (0.04 MB DOC) [file pgen.1000932.s003.doc]

**
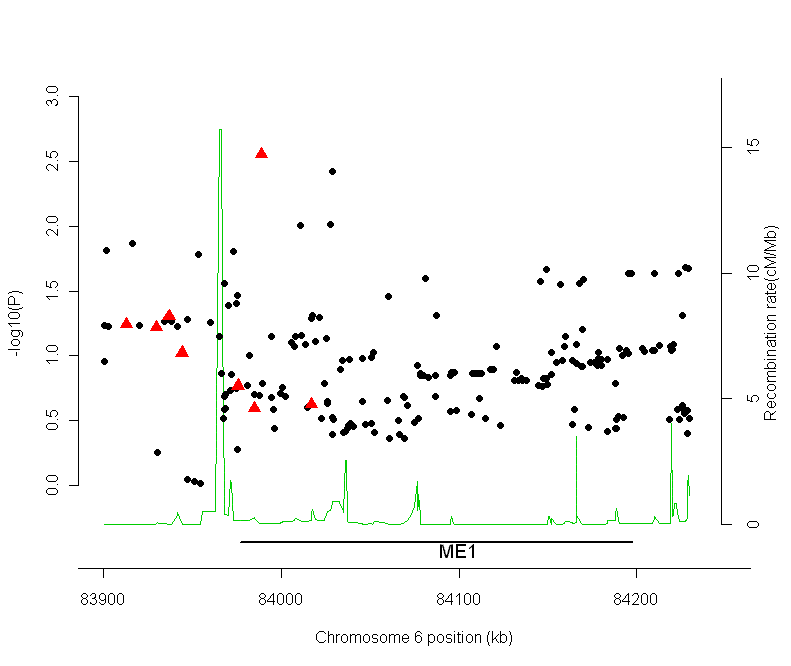
**

**Figure S3**. **Regional plot of *ME1* gene association with T2D in the DIAGRAM GWAS.** For *ME1* gene region on chromosome 6, genotyped and imputed SNPs are plotted with their meta-analysis PT2D values (as –log10 values) as a function of genomic position (NCBI Build 35). SNPs associated with *ME1* adipose expression are shown as red triangles. The estimated recombination rates (taken from HapMap) are plotted to reflect the local LD structure around the associated SNPs and their correlated proxies (Y axis on the right).
