# Supplementary material for: Liver and Adipose Expression Associated SNPs Are Enriched for Association to Type 2 Diabetes
Source: PLoS Genet. 2010 May 6;6(5):e1000932. doi: 10.1371/journal.pgen.1000932 (PMC2865508; doi:10.1371/journal.pgen.1000932)
Supplement: Table S1 — Tissue-specific eSNP discovery summary. (0.03 MB DOC) [file pgen.1000932.s004.doc]

**Table S1. Tissue-specific eSNP discovery summary.**

| *Cohort* | *Sample character* | *Sample Size* | *# eSNPs (FDR<0.1)* | *% cis-eSNP* |
| --- | --- | --- | --- | --- |
| Deliver | Caucasian | 427 | 3345 | 89.9% |
| MGH_Liver | Caucasian | 707 | 8693 | 90.9% |
| MGH_omental |  | 916 | 11742 | 91.4% |
| MGH_subcutaneous |  | 870 | 11392 | 89.9% |
