# Supplementary material for: Liver and Adipose Expression Associated SNPs Are Enriched for Association to Type 2 Diabetes
Source: PLoS Genet. 2010 May 6;6(5):e1000932. doi: 10.1371/journal.pgen.1000932 (PMC2865508; doi:10.1371/journal.pgen.1000932)
Supplement: Table S2 — The T2D causal adipose subnetwork (purple module) gene list, gene-trait correlations and causal genes. (1.82 MB DOC) [file pgen.1000932.s005.doc]

**Table S2. The T2d causal adipose subnetwork (purple module) gene list, gene-trait correlations and causal genes.**

| **Gene Symbol** | **Accession Code** | **Lipid, fatty acid and steroid metabolism** | **Plasma Insulin** | **Plasma Glucose** | **Number of Islets** | **Me1 -/- Signature** |
| --- | --- | --- | --- | --- | --- | --- |
| *1100001G20Rik* | AK003352 |  |  |  |  |  |
| *1500016O10Rik* | AK005271 |  |  |  |  | X |
| *2010003K11Rik* | AK008072 |  | X | X | X |  |
| *2010310D06Rik* | MMT00076568 |  |  |  |  | X |
| *2210022D18Rik* | AK008767 |  |  |  |  |  |
| *2310007D09Rik* | AK009199 |  |  |  |  |  |
| *2310046A06Rik* | AK009836 |  |  | X | X | X |
| *2310069G16Rik* | mCT186195 |  | X |  |  |  |
| *2810405K02Rik* | NM_025582 |  |  |  | X |  |
| *3010033K07Rik* | ri|3010033K07|ZX00083P10||952 |  |  |  |  |  |
| *4632404H22Rik* | NM_030167 |  | X |  |  |  |
| *4930502E18Rik* | MMT00063272 |  |  |  |  |  |
| *4930569K13Rik* | AK019795 |  |  |  |  |  |
| *5730508B09Rik* | AK017758 |  | X | X | X |  |
| *5730521E12Rik* | NM_025684 |  |  |  |  |  |
| *5830404H04Rik* | AI255484 |  |  |  |  |  |
| *6530401D17Rik* | MMT00029200 |  |  |  |  |  |
| *9130204L05Rik* | MMT00016733 |  |  | X |  |  |
| *9130213B05Rik* | MMT00054709 |  |  |  |  |  |
| *A030003A19Rik* | MMT00033772 |  |  |  |  | X |
| *A330094K24Rik* | AK020726 |  |  |  |  | X |
| *A830018L16Rik* | MMT00016482 |  |  | X |  |  |
| *Abca8a* | BC026496 | X |  |  |  |  |
| *Acly* | BC005533 | X |  |  |  |  |
| *Acoxl* | AK004775 | X | X | X |  |  |
| *Acss2* | NM_019811 | X | X |  | X | X |
| *Agpat2* | AK010891 | X |  |  | X | X |
| *AI844685* | MMS010234574 |  |  |  |  | X |
| *AK052811* | AK052811 |  |  |  | X |  |
| *Aldh1a7* | NM_011921 |  | X | X | X |  |
| *Alg14* | MMT00009902 |  |  |  |  | X |
| *Angptl1* | ENSMUST00000027885 |  |  |  |  |  |
| *Anxa2* | M14044 |  | X |  | X |  |
| *Asb5* | MMT00057142 |  |  |  |  | X |
| *Atp1a2* | MMT00010085 |  |  |  |  |  |
| *Atp1a4* | AF164349 |  |  | X | X |  |
| *Atp6v1c2* | BC003810 |  |  | X |  | X |
| *B230317C12Rik* | NM_019833 |  |  |  |  |  |
| *BB332301* | BB332301 |  | X |  |  |  |
| *BC031593* | BC019155 |  | X | X | X |  |
| *Bcar3* | NM_013867 |  |  |  |  |  |
| *Bcl2l10* | NM_013479 |  | X | X | X |  |
| *Brp44* | AK011889 |  |  | X |  | X |
| *C6* | NM_016704 |  | X |  |  |  |
| *C6* | AK041057 |  |  |  |  |  |
| *C730029A08Rik* | MMT00037777 |  | X |  | X |  |
| *Car12* | MMT00012431 |  |  |  |  |  |
| *Ccdc3* | MMT00018797 |  | X |  |  |  |
| *Cdkn1c* | U22399 |  |  |  | X | X |
| *Cdkn2b* | AF059567 |  | X | X | X |  |
| *Chrdl1* | NM_031258 |  | X | X | X | X |
| *Clstn3* | MMT00024150 |  | X | X | X |  |
| *Colec12* | MMT00032533 |  |  |  |  |  |
| *Cox8b* | NM_007751 |  | X |  | X |  |
| *Crlf1* | NM_018827 |  |  |  |  |  |
| *Cspg3* | NM_007789 |  | X | X | X |  |
| *Cspg3* | S65035 |  | X | X | X |  |
| *Cyb5r1* | MMT00017001 | X |  |  |  |  |
| *Cyb5r2* | MMT00009920 | X |  |  |  |  |
| *D10Ertd761e* | MMT00016807 |  |  |  |  |  |
| *D3Bwg0562e* | BE948923 | X |  |  |  | X |
| *Dbi* | AK020692 | X |  |  |  | X |
| *Dera* | NM_172733 |  | X | X | X | X |
| *Dfna5h* | AK016561 |  | X | X | X |  |
| *Dhcr7* | NM_007856 | X | X |  | X |  |
| *Dio2* | NM_010050 |  |  |  |  | X |
| *Dnmt3l* | MMT00041779 |  | X | X | X |  |
| *Dusp1* | NM_013642 |  |  |  |  |  |
| *Dusp10* | MMT00072790 |  |  |  |  |  |
| *E430004N04Rik* | MMT00030585 |  | X | X | X |  |
| *Edg7* | NM_022983 |  |  |  |  |  |
| *Elovl6* | NM_130450 | X | X |  |  |  |
| *Eya1* | MMT00008589 |  |  |  |  | X |
| *F830021D11Rik* | AK003868 |  |  | X |  |  |
| *Fabp5* | NM_010634 | X | X |  |  |  |
| *Fasn* | X13135 | X | X | X | X | X |
| *Fcna* | NM_007995 |  |  |  |  |  |
| *Fdps* | MMT00074306 | X |  |  |  |  |
| *Fxyd2* | NM_052824 |  | X | X | X |  |
| *Gdf8* | NM_010834 |  | X | X |  | X |
| *Gpd1* | AK018733 | X |  |  |  |  |
| *Gpr50* | NM_010340 |  | X |  |  |  |
| *Grb14* | NM_016719 |  | X |  |  |  |
| *Gys2* | MMT00019204 |  |  |  |  |  |
| *Hdac9* | MMT00081925 |  | X |  |  |  |
| *Hfe* | Y12650 |  |  |  |  | X |
| *Hist1h2bc* | AK005191 |  | X |  | X |  |
| *Hist2h3c2* | M32461 |  | X |  | X |  |
| *Hmgcs1* | MMT00075013 | X |  |  |  |  |
| *Hp* | NM_017370 |  |  |  |  |  |
| *Hsd17b12* | AK012103 | X | X |  |  | X |
| *Idi1* | BC004801 |  | X |  |  |  |
| *Igfals* | MMT00040765 |  |  |  |  |  |
| *Ighg* | ENSMUST00000004000 |  | X | X |  | X |
| *Il13ra2* | NM_008356 |  |  |  | X | X |
| *Insig1* | AK007471 | X |  |  |  | X |
| *Itga7* | NM_008398 |  |  |  |  |  |
| *Itgad* | MMT00055752 |  |  |  |  | X |
| *Kcne1l* | AK005246 |  |  | X |  |  |
| *Kcnj14* | ENSMUST00000059209 |  |  |  | X |  |
| *Kcnj14* | BC022700 |  |  |  |  |  |
| *Klhl25* | NM_182782 |  |  |  |  |  |
| *Lbp* | NM_008489 | X |  |  |  |  |
| *Lipf* | AK009923 | X |  | X | X |  |
| *Lrg1* | AK004940 |  |  |  |  |  |
| *Lss* | AK014742 |  | X |  | X |  |
| *Mass1* | MMT00043155 |  |  |  |  |  |
| *mCT160544* | mCT160544 |  |  |  |  |  |
| *mCT184940* | mCT184940 |  |  | X | X |  |
| *mCT185090* | mCT185090 |  |  |  |  | X |
| *Mel13* | ri|0610008K22|R000001P20||743 |  |  |  |  |  |
| *Mgst2* | MMT00012971 |  |  |  |  |  |
| *MMT00014809* | MMT00014809 |  |  |  |  |  |
| *MMT00018252* | MMT00018252 |  |  |  |  |  |
| *MMT00024444* | MMT00024444 |  | X |  |  |  |
| *MMT00040716* | MMT00040716 |  |  |  |  |  |
| *MMT00060103* | MMT00060103 |  | X |  |  | X |
| *MMT00060232* | MMT00060232 |  | X | X | X |  |
| *MMT00066571* | MMT00066571 |  |  |  |  |  |
| *MMT00073283* | MMT00073283 |  | X | X | X |  |
| *Mod1* | NM_008615 |  | X |  |  | X |
| *Mogat2* | MMT00010478 | X |  |  |  | X |
| *Mrap* | AK003912 |  | X | X | X |  |
| *Mvd* | AJ309922 | X |  |  |  |  |
| *Nfil3* | NM_017373 |  |  |  |  | X |
| *Nudt7* | AK004924 |  | X | X | X |  |
| *Otop1* | MMT00043089 |  | X | X | X |  |
| *P2ry5* | AK011967 |  |  |  |  |  |
| *Paqr9* | MMT00017267 | X | X | X | X | X |
| *Pcolce2* | AK010249 |  |  |  |  |  |
| *Peg3* | MMT00012853 |  | X |  | X | X |
| *Pkp1* | NM_019645 |  |  |  |  |  |
| *Pla2g2e* | NM_012044 | X | X | X | X |  |
| *Pla2g5* | AF162713 | X |  |  |  |  |
| *Ppp1r1a* | MMT00081566 |  |  |  |  |  |
| *Ppp1r3d* | AK011539 |  | X |  | X |  |
| *Prps1* | NM_021463 |  | X |  |  |  |
| *Ptgfr* | MMT00071244 |  |  |  |  |  |
| *Rab38* | NM_028238 |  |  |  |  |  |
| *Rbm28* | MMT00065722 |  |  | X |  |  |
| *S100a8* | MMT00078706 |  | X | X |  |  |
| *S100a8* | NM_013650 |  |  | X |  |  |
| *Scd2* | NM_009128 | X | X | X | X |  |
| *Serpinh1* | BC025470 |  |  |  |  |  |
| *Serpinh1* | NM_009825 |  |  |  |  |  |
| *Sfxn5* | MMT00009258 |  |  |  |  |  |
| *Slc13a4* | MMT00009669 |  |  |  |  |  |
| *Slc22a4* | NM_019687 |  | X | X |  |  |
| *Slc25a1* | AK005070 |  | X | X |  |  |
| *Slc2a3* | CL458941 |  |  | X |  |  |
| *Slc36a2* | NM_153170 |  |  |  | X |  |
| *Slc7a13* | MMT00026272 |  |  |  |  |  |
| *Sod2* | X04972 |  | X | X | X |  |
| *Taldo1* | MMT00054661 |  |  | X |  |  |
| *Tectb* | NM_009348 |  |  |  |  |  |
| *Thbs2* | NM_011581 |  |  |  |  |  |
| *Tnfaip8l1* | NM_025566 |  |  |  |  |  |
| *Tph2* | MMT00060338 |  | X |  | X | X |
| *Tsc22d1* | X62940 |  |  |  |  |  |
| *Tuba1* | M13445 |  |  |  |  | X |
| *Ubd* | NM_023137 |  | X | X | X |  |
| *V00827* | V00827 |  | X |  | X |  |
| *Vsig4* | MMT00004473 |  |  |  |  |  |
| *XM_131311* | XM_131311 |  |  |  |  |  |

**Table S6. *Me1* single gene perturbation signature in adipose tissue**

| **Accession Code** | **Gene Symbol** | **Accession Code** | **Gene Symbol** |
| --- | --- | --- | --- |
| BC002144 | 2-Mar | NM_008560 | Mc2r |
| AK019418 | 3-Sep | NM_023061 | Mcam |
| NM_011889 | 3-Sep | MMT00074117 | Mcart1 |
| AK002465 | 0610010F05Rik | NM_023644 | Mccc1 |
| ri|0910001K18|R000005H21||1507 | 0610031J06Rik | MMT00043943 | Mccc2 |
| NM_025806 | 1100001H23Rik | MMT00028849 | Mcf2l |
| AK020722 | 1110003F05Rik | MMS010278511 | Mdfi |
| AK003388 | 1110003O08Rik | NM_010783 | Mdfi |
| AK003497 | 1110006G14Rik | NM_008615 | Me1 |
| AK003598 | 1110008P14Rik | MMT00028364 | Me2 |
| MMT00007208 | 1110012M11Rik | AK002317 | Mecr |
| AK004537 | 1110018N20Rik | AK018323 | Med12l |
| AK003902 | 1110021L09Rik | AK007739 | Med30 |
| AK003943 | 1110028F11Rik | AK004354 | Med7 |
| MMT00022028 | 1110036O03Rik | AK013855 | Megf10 |
| AK004150 | 1110038B12Rik | MMT00003101 | Megf8 |
| AK012023 | 1110054O05Rik | BC002076 | Meox2 |
| AK004285 | 1110057K04Rik | AK031718 | Mest |
| MMT00015899 | 1110059G02Rik | AK050959 | Mett10d |
| AI462559 | 1110065F06Rik | AK002682 | Mett5d1 |
| NM_025427 | 1190002H23Rik | ENSMUST00000060447 | Mettl5 |
| AK004457 | 1190002N15Rik | AK012557 | Mex3a |
| AK004494 | 1190005I06Rik | MMT00068568 | Mex3a |
| AK004674 | 1200009I06Rik | AK003537 | Mfap4 |
| BC004837 | 1200013P24Rik | MMT00062867 | Mfn2 |
| AK004841 | 1300001I01Rik | MMT00028763 | Mfng |
| AK004929 | 1300007F04Rik | AK007898 | Mfsd11 |
| MMT00046293 | 1300010F03Rik | AK013144 | Mfsd8 |
| AK005003 | 1300014I06Rik | MMT00049771 | Mgl2 |
| AK005014 | 1300015D01Rik | MMT00078055 | Mgll |
| mCT184095 | 1500002F19Rik | MMT00022295 | Mgst3 |
| ri|1500003O03|ZX00042C11||2574 | 1500003O03Rik | NM_029392 | Mgst3 |
| AK005154 | 1500005A01Rik | AK019197 | Mgst3 |
| AK005164 | 1500005K14Rik | BC043122 | Mical3 |
| AK005271 | 1500016O10Rik | NM_011845 | Mid2 |
| AK005350 | 1500032O14Rik | NM_010798 | Mif |
| MMT00073436 | 1600014C10Rik | NM_018810 | Mkrn1 |
| MMT00082321 | 1600021P15Rik | AF494488 | Mkrn1-ps1 |
| AK005515 | 1600021P15Rik | AK020928 | Mlana |
| mCT185090 | 1600025M17Rik | BC009120 | Mll2 |
| AK005564 | 1600029O15Rik | MMT00031637 | Mllt3 |
| NM_029312 | 1700010H22Rik | AK011386 | Mllt3 |
| AK005901 | 1700012E03Rik | ENSMUST00000054707 | Mllt4 |
| AK006166 | 1700020I14Rik | BC004764 | Mlycd |
| AK006572 | 1700030P01Rik | MMT00048035 | Mmaa |
| NM_026091 | 1700037H04Rik | AK020286 | Mmab |
| NM_175196 | 1700041G16Rik | MMT00068143 | Mmachc |
| mCT185966 | 1700044C05Rik | NM_026178 | Mmd |
| AK006704 | 1700047E10Rik | MMT00025993 | Mmp15 |
| AK006707 | 1700047G03Rik | NM_008611 | Mmp8 |
| AK006709 | 1700047G07Rik | NM_013599 | Mmp9 |
| AK006713 | 1700047I17Rik1 | MMT00044287 | Mmrn2 |
| AK006950 | 1700074L02Rik | MMT00048866 | Mobkl2b |
| BF458552 | 1700087I21Rik | AK018460 | Mobkl2b |
| AK007103 | 1700100M05Rik | MMT00006001 | Mogat1 |
| MMT00033355 | 1700101E01Rik | MMT00010478 | Mogat2 |
| AK007275 | 1700125C05Rik | NM_198162 | Morc2a |
| AK007284 | 1700125M20Rik | ri|1600017G11|ZX00042F05||1278 | Morc4 |
| ENSMUST00000040372 | 1810006K21Rik | AK014340 | Moxd1 |
| AK007358 | 1810007C17Rik | MMT00077242 | Mphosph6 |
| ri|1110017P05|R000016K20||1216 | 1810013L24Rik | NM_008621 | Mpp1 |
| NM_025459 | 1810015C04Rik | AB059357 | Mpp4 |
| MMT00019316 | 1810022C23Rik | MMT00042356 | Mr1 |
| AK007675 | 1810032O08Rik | AK004053 | Mrpl20 |
| AK007899 | 1810058N05Rik | MMT00078507 | Mrpl3 |
| AK007952 | 1810064F22Rik | NM_010922 | Mrpl40 |
| AF313412 | 2010002N04Rik | AK004194 | Mrpl52 |
| AK008061 | 2010003D24Rik | MMT00021486 | Mrpl55 |
| MMT00072633 | 2010016I18Rik | MMT00047360 | Mrps23 |
| MMT00077412 | 2010107E04Rik | NM_025578 | Mrps25 |
| AK008369 | 2010109N14Rik | MMT00002291 | Ms4a8a |
| MMT00081391 | 2010305C02Rik | MMT00017788 | Msln |
| BC004058 | 2210010N04Rik | NM_018857 | Msln |
| AK008706 | 2210011G09Rik | MMT00032026 | Msrb3 |
| MMT00041061 | 2210016H18Rik | NM_009074 | Mst1r |
| MMT00037476 | 2210408F21Rik | NM_010834 | Mstn |
| AK009071 | 2310001K24Rik | NM_008633 | Mtap4 |
| AK009156 | 2310004N24Rik | AK018193 | Mtap6 |
| AK009157 | 2310004O12Rik | MMT00070404 | Mtg1 |
| AK009219 | 2310008B10Rik | NM_008638 | Mthfd2 |
| AK009333 | 2310014D11Rik | NM_026849 | Mtmr14 |
| ENSMUST00000056121 | 2310014G06Rik | MMT00025613 | Muc16 |
| AK009378 | 2310016C16Rik | AJ277212 | Mustn1 |
| AK009532 | 2310029O18Rik | NM_008650 | Mut |
| AK009643 | 2310035P21Rik | NM_207215 | Mycbp2 |
| NM_026509 | 2310039E09Rik | MMT00031092 | Mycbp2 |
| AK009710 | 2310040A07Rik | MMT00076562 | Myct1 |
| MMT00058021 | 2310040A07Rik | BC005591 | Myd88 |
| AK009724 | 2310040G24Rik | MMT00046028 | Myh14 |
| AK009829 | 2310045N01Rik | NM_022879 | Myl7 |
| AK009836 | 2310046A06Rik | MMT00024785 | Myo10 |
| MMT00007620 | 2310046K01Rik | MMS010098291 | Myo1b |
| MMT00039589 | 2310047A01Rik | NM_010863 | Myo1b |
| AK009989 | 2310061A09Rik | AK004743 | Myo1c |
| AK010063 | 2310066E14Rik | MMT00046204 | Myo1d |
| AK010086 | 2310067E19Rik | BC034907 | Myo3b |
| AK010184 | 2310075M01Rik | NM_017479 | Myst4 |
| AK019104 | 2400009B08Rik | MMT00040163 | N4bp2l1 |
| AK019107 | 2410006F04Rik | NM_026366 | N6amt1 |
| MMT00073752 | 2410006H16Rik | AK008776 | Naaa |
| AK010455 | 2410008K03Rik | mCT17467 | Naaladl1 |
| NM_023633 | 2410016O06Rik | MMT00000283 | Naaladl2 |
| AK010587 | 2410024N18Rik | NM_008668 | Nab2 |
| MMT00062457 | 2410137M14Rik | NM_013608 | Naca |
| AK019137 | 2500002B13Rik | BC019163 | Nae1 |
| AK010982 | 2510019K15Rik | NM_026086 | Nanp |
| BC056393 | 2600009P04Rik | AK008261 | Nap1l1 |
| AK011178 | 2600010E01Rik | NM_021432 | Nap1l5 |
| AK014682 | 2600010E01Rik | MMT00002313 | Napepld |
| AK011250 | 2610001A08Rik | AK004167 | Nat8l |
| mCT23149 | 2610002J02Rik | MMT00040930 | Nat8l |
| mCT174614 | 2610016A17Rik | MMT00023261 | Nav3 |
| MMT00005625 | 2610019F03Rik | MMT00039801 | Nbeal1 |
| AK011524 | 2610024B07Rik | NM_010877 | Ncf2 |
| MMT00076005 | 2610030H06Rik | BE630442 | Ncl |
| AK011855 | 2610201A13Rik | NM_010881 | Ncoa1 |
| AK052622 | 2610208M17Rik | AK004086 | Ncoa4 |
| AK012147 | 2610524H06Rik | NM_011308 | Ncor1 |
| BE990725 | 2610524H06Rik | MMT00050730 | ND5 |
| AK019193 | 2610524J22Rik | XM_358137 | Ndfip2 |
| AK012304 | 2700029M09Rik | AK005078 | Ndn |
| XM_358763 | 2700045P11Rik | NM_013864 | Ndrg2 |
| AK012625 | 2810001A02Rik | BC006595 | Ndrg4 |
| AK007927 | 2810001G20Rik | MMT00022033 | Ndufaf1 |
| MMT00082740 | 2810003C17Rik | MMT00082767 | Ndufb8 |
| MMT00077048 | 2810004N23Rik | AK005021 | Ndufv1 |
| AK020712 | 2810022L02Rik | MMT00037414 | Nedd4 |
| MMT00070341 | 2810032G03Rik | NM_031881 | Nedd4l |
| AK013066 | 2810410D24Rik | MMT00062950 | Negr1 |
| AK013171 | 2810427C15Rik | NM_021605 | Nek7 |
| AK019273 | 2810433H14Rik | MMT00023947 | Nek9 |
| AK013351 | 2810455B08Rik | MMT00031128 | Neurog3 |
| AK013380 | 2810465G24Rik | MMT00050479 | Nfasc |
| AK013449 | 2810488G03Rik | D85612 | Nfatc3 |
| AK013488 | 2900006B11Rik | NM_008686 | Nfe2l1 |
| AK013498 | 2900008C10Rik | AK004196 | Nfia |
| AK013553 | 2900019A20Rik | NM_010905 | Nfia |
| AK013579 | 2900022P04Rik | MMT00031241 | Nfia |
| ri|8430408O14|PX00024O20||1484 | 2900026A02Rik | ri|6720429L07|PX00059D17||1714 | Nfib |
| AK013604 | 2900027M19Rik | NM_008687 | Nfib |
| AK013617 | 2900035J10Rik | AK005387 | Nfic |
| AK013628 | 2900040J22Rik | NM_008688 | Nfic |
| AK013640 | 2900042K05Rik | NM_017373 | Nfil3 |
| AK013658 | 2900046L07Rik | BC003766 | Nfix |
| AK013682 | 2900054C01Rik | NM_009750 | Ngfrap1 |
| AK013539 | 2900060N18Rik | L10905 | Ngfrap1 |
| AK013740 | 2900062L11Rik | NM_021504 | Ngly1 |
| AK013743 | 2900063K03Rik | MMT00064408 | Nhedc2 |
| AK013747 | 2900064B18Rik | AK004564 | Nhlrc2 |
| AK019356 | 2900079J23Rik | XM_196564 | Nhp2l1 |
| AK013819 | 2900084I15Rik | NM_010917 | Nid1 |
| NM_175404 | 3010003L21Rik | NM_008695 | Nid2 |
| AK019402 | 3010031K01Rik | AK016861 | Nipbl |
| AK019403 | 3010033E01Rik | MMT00002818 | Nkain2 |
| AK013984 | 3110003A17Rik | MMT00011085 | Nkiras1 |
| AK014000 | 3110005L21Rik | NM_008701 | Nkx2-9 |
| AK014125 | 3110035G12Rik | NM_198862 | Nlgn2 |
| MMT00039800 | 3110037I16Rik | NM_018757 | Nme6 |
| AK014176 | 3110045A19Rik | NM_010923 | Nnat |
| AK014252 | 3110073H01Rik | AK046462 | Nnat |
| AK014270 | 3110082D06Rik | NM_010924 | Nnmt |
| MMT00062784 | 3110082I17Rik | MMS010087789 | Nog |
| MMT00045222 | 3200002M19Rik | MMT00064486 | Nol3 |
| AK013025 | 3321401G04Rik | MMT00082164 | Nol6 |
| AK014379 | 3321401G04Rik | MMT00027437 | Nomo1 |
| NM_026142 | 3632451O06Rik | AK020142 | Nope |
| MMT00021191 | 4631405J19Rik | MMT00080503 | Nope |
| ri|4631423B10|PX00011N12||3239 | 4631423B10Rik | MMT00074291 | Nos3 |
| AK014565 | 4632411B12Rik | MMT00074886 | Notch4 |
| AK019501 | 4632418H02Rik | X96585 | Nov |
| NM_199009 | 4632419K20Rik | MMT00012229 | Npc1l1 |
| AK014609 | 4633401B06Rik | AK008603 | Npc2 |
| AK028644 | 4732423E21Rik | NM_008721 | Npdc1 |
| MMT00051488 | 4732474O15Rik | MMT00054036 | Npepl1 |
| MMT00030448 | 4833409A17Rik | MMT00009992 | Nphp3 |
| AK014684 | 4833412C15Rik | NM_028749 | Npl |
| MMT00055034 | 4833414E09Rik | NM_008722 | Npm1 |
| AK014707 | 4833416E15Rik | NM_008728 | Npr3 |
| AK014710 | 4833416J08Rik | AI606967 | Npr3 |
| AK017802 | 4833420G17Rik | MMT00031406 | Nptx1 |
| AK014774 | 4833426J09Rik | BC008989 | Nr1d1 |
| AK014787 | 4833428M15Rik | AK012573 | Nr2f2 |
| AI451221 | 4921509B22Rik | NM_010444 | Nr4a1 |
| AK014853 | 4921509J17Rik | AK012426 | Nrarp |
| AK014857 | 4921509O09Rik | MMS010192763 | Nrarp |
| MMT00032259 | 4930402E16Rik | BC027677 | Nrg4 |
| NM_029432 | 4930402H24Rik | MMT00081728 | Nt5c2 |
| mCT184734 | 4930429D17Rik | ENSMUST00000037103 | Nt5dc2 |
| MMT00029858 | 4930431H11Rik | NM_008743 | Nthl1 |
| AK015287 | 4930432H08Rik | MMT00000347 | Ntn2l |
| AK029002 | 4930442M18Rik | MMT00006628 | Ntrk2 |
| mCT184117 | 4930447F24Rik | MMT00026735 | Nuak1 |
| AK015453 | 4930453J04Rik | NM_016773 | Nucb2 |
| AK015477 | 4930456J16Rik | MMT00030006 | Nudt14 |
| AK076593 | 4930467E23Rik | NM_029385 | Nudt16 |
| AK015596 | 4930480C01Rik | NM_153136 | Nudt18 |
| AK015629 | 4930486A15Rik | NM_016918 | Nudt5 |
| AK015635 | 4930486L24Rik | BB043522 | Nudt6 |
| AK019720 | 4930535G08Rik | AK011422 | Nup43 |
| ri|4931406P16|PX00016I11||5808 | 4931406P16Rik | ENSMUST00000058130 | Nutf2 |
| AK016444 | 4931408D14Rik | MMT00044471 | Nxnl1 |
| NM_028905 | 4932438H23Rik | MMT00013549 | Obfc2a |
| AK016764 | 4933411B09Rik | AK007617 | Ociad2 |
| AK016774 | 4933411K05Rik | D87034 | Odz4 |
| ENSMUST00000061046 | 4933412A08Rik | NM_010957 | Ogg1 |
| MMT00054676 | 4933417O08Rik | NM_019498 | Olfm1 |
| AK016911 | 4933425E08Rik | AW743226 | Olfml1 |
| NM_021493 | 4933428G20Rik | MMT00078816 | Olfml3 |
| AK017144 | 5031434O11Rik | NM_010999 | Olfr56 |
| mCT49517 | 5033404E19Rik | NM_147059 | Olfr668 |
| MMT00035856 | 5033406O09Rik | NM_130866 | Olfr78 |
| AK017178 | 5033418A18Rik | ENSMUST00000064769 | Olfr885 |
| AK017228 | 5230400M06Rik | NM_146612 | Olfr968 |
| AK017233 | 5330403D14Rik | AW319146 | Ophn1 |
| AK019897 | 5330417P21Rik | NM_010098 | Opn3 |
| AK030568 | 5330432J10Rik | AK002718 | Oprs1 |
| AK017280 | 5430406J06Rik | NM_008768 | Orm1 |
| MMT00001125 | 5430416N02Rik | AI132306 | Osbpl11 |
| MMT00058752 | 5430416O09Rik | NM_020573 | Osbpl1a |
| AK019946 | 5430417C01Rik | AJ278263 | Osbpl5 |
| AK017364 | 5430430B14Rik | AK004353 | Osbpl6 |
| BB463553 | 5430433E21Rik | MMT00069369 | Osbpl6 |
| MMT00035485 | 5430433G21Rik | MMT00079275 | Osgepl1 |
| AK017390 | 5430434G16Rik | MMT00057094 | Osgin2 |
| AK017428 | 5530400N10Rik | MMT00055633 | OTTMUSG00000000421 |
| AK017539 | 5730409N24Rik | MMT00062278 | OTTMUSG00000003387 |
| BE943724 | 5730410E15Rik | XM_112237 | OTTMUSG00000010671 |
| AK017641 | 5730446C15Rik | MMT00038070 | OTTMUSG00000011664 |
| AK017688 | 5730469M10Rik | MMT00043474 | OTTMUSG00000015946 |
| AK017720 | 5730492I20Rik | MMT00077664 | OTTMUSG00000022462 |
| ri|5730536A07|PX00006A05||1241 | 5730536A07Rik | ENSMUST00000070970 | Otud7b |
| AK019973 | 5730564C23Rik | AK011354 | Oxct1 |
| MMT00080469 | 5730596K20Rik | MMT00013489 | P2ry13 |
| MMS010194976 | 5730596K20Rik | NM_008776 | Pafah1b3 |
| mCT22451 | 5830405M20Rik | MMT00016775 | Pag1 |
| AK020000 | 5830407P18Rik | ri|5930415H02|PX00055I09||1619 | Pak1ip1 |
| AK017401 | 5830407P18Rik | MMT00017361 | Palld |
| AK017935 | 5830416P10Rik | NM_023128 | Palm |
| AK017967 | 5830433I10Rik | MMT00022974 | Palmd |
| AK018026 | 5830461L22Rik | MMT00011559 | Pank3 |
| AK018053 | 5830474E16Rik | AK010469 | Paox |
| AK031220 | 5930430O07Rik | AK007539 | Papss2 |
| AK020044 | 5930436O19Rik | NM_011864 | Papss2 |
| NM_183121 | 6030426L16Rik | MMT00038091 | Paqr7 |
| AK077906 | 6030432P03Rik | MMT00017267 | Paqr9 |
| MMT00007857 | 6030443J06Rik | AK008055 | Pard3b |
| AK031515 | 6030446J10Rik | MMT00004254 | Parp8 |
| AK080477 | 6030451C04Rik | MMT00055429 | Pbld |
| AK020075 | 6030458E02Rik | NM_016768 | Pbx3 |
| AK018090 | 6230416C02Rik | AI256222 | Pcca |
| ri|9130410H17|PX00026J04||1633 | 6230424C14Rik | NM_053145 | Pcdhb20 |
| AK018107 | 6330403L08Rik | MMT00081557 | Pcdhb7 |
| AK018133 | 6330407A03Rik | MMT00016429 | Pcdhb9 |
| MMS010115112 | 6330417G02Rik | MMT00014831 | Pcgf5 |
| ri|6330417G04|PX00008H08||992 | 6330417G04Rik | NM_011044 | Pck1 |
| MMT00047954 | 6330419J24Rik | NM_011995 | Pclo |
| MMT00021979 | 6330442E10Rik | NM_018814 | Pcnx |
| AK031957 | 6330509M05Rik | MMT00019181 | Pcnxl2 |
| AK020094 | 6330522J23Rik | NM_008796 | Pctp |
| ENSMUST00000059190 | 6330549D23Rik | NM_008797 | Pcx |
| ri|6330577E15|PX00044A08||1584 | 6330577E15Rik | ri|4930503H15|PX00032L20||1858 | Pcyt1a |
| MMT00045331 | 6330578E17Rik | MMT00058493 | Pde3b |
| AK018259 | 6330582A15Rik | AJ132271 | Pde3b |
| AK078202 | 6430502G17Rik | AK036412 | Pde3b |
| NM_176932 | 6430510M02Rik | X95521 | Pde3b |
| AK030418 | 6430537K16Rik | NM_008802 | Pde7a |
| AK032427 | 6430543K15Rik | NM_008804 | Pde9a |
| MMT00012063 | 6430571L13Rik | NM_019971 | Pdgfc |
| AK032649 | 6530402L11Rik | NM_011058 | Pdgfra |
| AK018351 | 6620401M08Rik | AK004179 | Pdgfrl |
| AK020106 | 6720407P12Rik | AK011810 | Pdhb |
| AK020112 | 6720420G18Rik | MMT00008463 | Pdia5 |
| AK032738 | 6720422M22Rik | NM_172665 | Pdk1 |
| AK020116 | 6720427H10Rik | NM_019417 | Pdlim4 |
| AK020136 | 6720460K10Rik | MMT00052655 | Pdzd2 |
| AK032850 | 6720462K09Rik | AK021207 | Pdzd2 |
| MMT00075734 | 6720464F23Rik | NM_026018 | Pdzk1ip1 |
| MMT00047741 | 6720469N11Rik | NM_018884 | Pdzrn3 |
| AK020162 | 6720477C19Rik | X86694 | Pea15a |
| AK020175 | 6720484G13Rik | NM_008816 | Pecam1 |
| NM_175252 | 6720489N17Rik | MMT00012853 | Peg3 |
| AK033153 | 8030447M02Rik | ri|4833429C02|PX00028L15||1931 | Peg3 |
| AK020211 | 8030459D09Rik | AK020060 | Peli3 |
| AK033243 | 8030474H12Rik | AK012936 | Per3 |
| AK020220 | 8030487O14Rik | BC005709 | Pet112l |
| AK018397 | 8430408J07Rik | NM_011068 | Pex11a |
| AK018429 | 8430420F16Rik | MMT00002159 | Pex12 |
| AK018474 | 8430437O03Rik | AK012785 | Pex19 |
| MMT00014252 | 9030224M15Rik | AF294617 | Pfkfb3 |
| AK018518 | 9030418K01Rik | NM_019410 | Pfn2 |
| mCT55108 | 9030418K01Rik | AK014543 | Pgrmc2 |
| AK018527 | 9030425E11Rik | MMT00019679 | Phactr2 |
| MMT00016393 | 9030617O03Rik | AK021317 | Phf17 |
| AK018589 | 9130004J05Rik | NM_011079 | Phkg1 |
| AK018604 | 9130009M17Rik | BC025856 | Phldb1 |
| AK013327 | 9130017K11Rik | BC004581 | Phlpp |
| AK020313 | 9230104K21Rik | NM_008835 | Phxr4 |
| AK034149 | 9330159M07Rik | NM_010726 | Phyh |
| AK048946 | 9330161A08Rik | AK005293 | Phyhd1 |
| AK034385 | 9330185G11Rik | NM_026078 | Pigc |
| AK034463 | 9330197I21Rik | NM_027388 | Pigw |
| BC004010 | 9430015G10Rik | AI467556 | Pik3r4 |
| AK004276 | 9430020K01Rik | MMT00063661 | Pik3r6 |
| AK050253 | 9430037G07Rik | BC017621 | Pim3 |
| AK034849 | 9430047G12Rik | MMT00032717 | Pink1 |
| AK020505 | 9430085L16Rik | BC003763 | Pip5k1a |
| AK035104 | 9430088L24Rik | NM_011256 | Pitpnm2 |
| AK020532 | 9530001D17Rik | MMT00055986 | Pitpnm3 |
| AK079170 | 9530001J02Rik | MMT00035350 | Piwil4 |
| AK020546 | 9530006C21Rik | BC004742 | Pja2 |
| AK035329 | 9530018D06Rik | MMT00033513 | Pkhd1l1 |
| AK020585 | 9530034D02Rik | NM_008863 | Pkib |
| AK079217 | 9530037G02Rik | MMT00077748 | Pkn2 |
| AK020592 | 9530039L23Rik | MMT00050523 | Pkn3 |
| AK020648 | 9530077C14Rik | NM_016670 | Pknox1 |
| AK020657 | 9530086O07Rik | AK004650 | Pkp2 |
| MMT00080703 | 9530096D07Rik | AK005210 | Pla2g7 |
| NM_176994 | 9630013D21Rik | AF263458 | Plac8 |
| AK051384 | 9630019E01Rik | MMS010148211 | Plcb1 |
| AK084616 | 9630023C09Rik | NM_013829 | Plcb4 |
| MMT00049873 | 9630025H16Rik | NM_019676 | Plcd1 |
| MMT00004023 | 9630050E16Rik | AF233885 | Plce1 |
| MMT00012735 | 9630058J23Rik | MMT00065283 | Plcxd1 |
| AK034818 | 9830001H06Rik | MMT00056362 | Plekha1 |
| mCT183998 | 9930035N15Rik | MMT00026117 | Plekha4 |
| AK037343 | A1300008O04Rik | MMT00055698 | Plekha6 |
| AK037349 | A130009I22Rik | NM_198604 | Plekhg6 |
| AK038168 | A130040G06Rik | MMT00080527 | Plin |
| AK037862 | A130057A22Rik | MMT00067468 | Plin |
| AK041335 | A130090K04Rik | AK002477 | Pllp |
| MMT00024865 | A230050P20Rik | D86949 | Plxna2 |
| AK038768 | A230061C15Rik | MMT00059475 | Pm20d1 |
| AK054440 | A230067G21Rik | AF007267 | Pmm1 |
| AK079558 | A230071A22Rik | AK012683 | Pmm2 |
| AK039653 | A330078L11Rik | MMT00070342 | Pnldc1 |
| AK020726 | A330094K24Rik | AK002826 | Pnpla2 |
| AK039872 | A430023P06Rik | AF290209 | Podxl |
| AK033768 | A430105I19Rik | NM_008893 | Pola2 |
| AI265501 | A430107O13Rik | MMT00056001 | Pole2 |
| MMT00064014 | A530013C23Rik | MMT00053650 | Polg |
| MMT00005160 | A530016L24Rik | MMT00052284 | Polr2g |
| AK040933 | A530047J11Rik | NM_011293 | Polr2j |
| AK080119 | A530064N14Rik | NM_026190 | Polr3g |
| AK041809 | A630039O03Rik | AK008069 | Polr3gl |
| mCT183953 | A630077J23Rik | MMT00048069 | Pon3 |
| AK042355 | A630084N20Rik | NM_022318 | Popdc2 |
| AK042670 | A730014G21Rik | NM_008898 | Por |
| ENSMUST00000066106 | A730061H03Rik | NM_011141 | Pou3f1 |
| MMT00082667 | A830007P12Rik | NM_008900 | Pou3f3 |
| AK043918 | A830051L15Rik | NM_008902 | Pp11r |
| BF460485 | A930001M12Rik | NM_011144 | Ppara |
| MMT00029591 | A930007A09Rik | MMT00080979 | Ppara |
| AK020824 | A930007D18Rik | AK030241 | Ppara |
| AK020830 | A930008G19Rik | U01841 | Pparg |
| AK020860 | A930015D03Rik | U01841 | Pparg |
| AK020900 | A930028N01Rik | U01841 | Pparg |
| NM_177232 | A930033H14Rik | NM_133249 | Ppargc1b |
| MMT00023895 | A930034L06Rik | NM_011149 | Ppib |
| AK080763 | A930104B17Rik | ri|2410007M08|ZX00033A20||2354 | Ppm1a |
| MMS010121774 | AA407107 | AJ271836 | Ppm1b |
| MMS010227553 | AA408213 | NM_176833 | Ppm1f |
| MMS010194696 | AA409368 | MMT00054276 | Ppm1h |
| AK079655 | AA589532 | AK021038 | Ppm1h |
| MMT00050881 | Aacs | MMT00009828 | Ppm1k |
| ri|5033417E09|PX00037J06||1727 | Aadac | AK013741 | Ppm1k |
| MMT00062240 | Abat | MMT00049229 | Ppm1l |
| MMT00022469 | Abca6 | MMT00015563 | Ppm1l |
| AF213393 | Abca8b | AI467229 | Ppp1r3a |
| AK008292 | Abcb1a | MMT00009279 | Ppp1r3e |
| NM_011076 | Abcb1a | MMT00014693 | Ppp1r9b |
| AK006128 | Abcc3 | MMT00062023 | Ppp2r2c |
| NM_021043 | Abcc9 | BC059026 | Ppp2r5a |
| ENSMUST00000066484 | Abcd2 | AK012478 | Ppp2r5c |
| NM_011994 | Abcd2 | NM_009358 | Ppp2r5d |
| AK018283 | Abcg4 | X80432 | Ppp2r5d |
| BB228083 | Abhd11 | mCT3155 | Prdm13 |
| MMT00066627 | Abhd14b | AK020180 | Prelp |
| MMT00032364 | Abhd3 | ENSMUST00000051186 | Prkaa1 |
| ri|2010005F17|ZX00043H02||1460 | Abhd6 | MMT00082781 | Prkag2 |
| mCT15012 | Abhd9 | MMT00042807 | Prkag3 |
| MMT00063289 | Ablim2 | ri|2700080N03|ZX00064K15||2063 | Prkar2a |
| MMT00072845 | Ablim3 | AK011647 | Prkar2b |
| MMT00033948 | Abr | MMT00024287 | Prkca |
| MMT00073060 | Acaa1b | AK079091 | Prkca |
| MMT00067953 | Acacb | NM_028444 | Prkcdbp |
| MMT00061203 | Acad10 | AK017901 | Prkce |
| ENSMUST00000031412 | Acad10 | MMT00027873 | Prkce |
| MMT00009358 | Acad11 | MMT00010484 | Prkrir |
| AK004889 | Acadsb | NM_178921 | Prlr |
| AK078636 | Acadsb | AI448514 | Prmt2 |
| AK008243 | Acbd4 | NM_021381 | Prokr1 |
| AK011867 | Accs | MMT00004816 | Prp2 |
| BC004645 | Aco2 | MMT00080284 | Prr16 |
| AF006688 | Acox1 | U03873 | Prrx1 |
| AF006688 | Acox1 | NM_008939 | Prss12 |
| AF006688 | Acox1 | AK021185 | Prss36 |
| AK011915 | Acp5 | MMT00014842 | Prtn3 |
| NM_207668 | Acpp | MMT00063816 | Pscdbp |
| MMT00027539 | Acsf3 | BC030357 | Psg16 |
| NM_016870 | Acsm3 | NM_007676 | Psg16 |
| NM_019811 | Acss2 | NM_011967 | Psma5 |
| MMT00081813 | Acvr1c | MMT00006659 | Psmb5 |
| BB222972 | Acvr1c | U87814 | Pstpip1 |
| NM_007397 | Acvr2b | NM_008960 | Pten |
| AK002247 | Acy3 | D10204 | Ptger3 |
| NM_009614 | Adam15 | MMT00053446 | Ptges |
| AK036428 | Adamts5 | NM_175168 | Ptk7 |
| NM_011782 | Adamts5 | NM_011200 | Ptp4a1 |
| MMT00081767 | Adamtsl4 | MMT00031624 | Ptp4a3 |
| MMT00036144 | Adarb1 | MMT00063688 | Ptpla |
| MMT00064210 | Adcy4 | MMT00081985 | Ptpmt1 |
| XM_358801 | Adcy5 | AK047658 | Ptpn14 |
| MMT00008499 | Adcy5 | MMT00080789 | Ptpn23 |
| NM_007405 | Adcy6 | MMT00002842 | Ptpn3 |
| AI506561 | Adh6b | AF157628 | Ptprb |
| MMT00074138 | Adhfe1 | D13903 | Ptprd |
| NM_009605 | Adipoq | AK003303 | Ptprd |
| AK003228 | Adipor2 | MMT00008025 | Ptprh |
| MMT00047494 | Ado | AK010169 | Ptrf |
| MMT00029862 | Adora1 | AK009381 | Ptrf |
| NM_009633 | Adra2b | AK004821 | Pvrl4 |
| AK018378 | Adrb1 | AK010185 | Pxdn |
| NM_013462 | Adrb3 | AK019381 | Pxmp4 |
| MMT00064534 | Adrbk2 | MMT00061953 | Pycard |
| NM_009634 | Adsl | AI846739 | Pygb |
| MMS010035085 | AF313042 | ENSMUST00000038489 | Pygo1 |
| MMT00071976 | Afap1l1 | AK010602 | Qprt |
| AK014232 | Afg3l1 | AK004880 | Qsox1 |
| MMT00021044 | Aftph | ENSMUST00000050569 | R3hcc1 |
| ri|2310079F20|ZX00060G03||1208 | Aga | AK017590 | Rab19 |
| AK010891 | Agpat2 | MMT00072741 | Rab20 |
| ri|4933407I02|PX00019L22||1832 | Agpat9 | NM_023635 | Rab27a |
| MMT00044702 | Ahcyl2 | NM_033475 | Rab34 |
| AK003448 | Ahnak | MMT00021087 | Rab35 |
| MMT00034763 | AI036347 | AK012165 | Rab3b |
| MMT00019684 | AI195381 | NM_031874 | Rab3d |
| MMT00014960 | AI314604 | D86563 | Rab4a |
| MMS010066384 | AI415730 | AJ245569 | Rab6ip1 |
| MMT00038613 | AI426330 | MMT00052136 | Rab9b |
| MMT00080861 | AI464131 | AK020007 | Rabgap1l |
| AK089957 | AI464196 | MMT00010125 | Rac3 |
| MMT00017374 | AI480653 | NM_009012 | Rad50 |
| MMT00053733 | AI661453 | NM_011235 | Rad51l3 |
| AI747448 | AI747448 | NM_009016 | Raet1a |
| MMT00016728 | AI790298 | NM_009017 | Raet1b |
| MMS010234574 | AI844685 | NM_009018 | Raet1c |
| MMT00023286 | AI852064 | NM_020030 | Raet1d |
| AK010936 | Ak2 | NM_022327 | Ralb |
| MMT00007141 | Ak3 | NM_019444 | Ramp2 |
| MMT00047323 | Akap5 | MMT00031898 | Ramp3 |
| NM_018747 | Akap7 | MMT00015400 | Rap1gap |
| MMT00056045 | Akr1c14 | AK018008 | Rapgef2 |
| NM_007434 | Akt2 | AK021094 | Rapgef6 |
| MMT00023600 | AL022681 | MMT00006983 | Rarg |
| AK002642 | Alas2 | MMT00034860 | Rasa2 |
| NM_019698 | Aldh18a1 | NM_009025 | Rasa3 |
| MMT00012599 | Aldh1a2 | BF659595 | Rasal2 |
| MMT00043677 | Aldh1l1 | MMT00037564 | Rasgef1b |
| AV258846 | Aldh3b2 | Y12339 | Rasgrp2 |
| NM_183142 | Alg11 | MMT00031751 | Rasip1 |
| MMT00009902 | Alg14 | NM_009029 | Rb1 |
| MMT00053459 | Alpk2 | NM_019705 | Rbck1 |
| MMT00070963 | Als2cl | MMT00070121 | Rbed1 |
| MMT00075933 | Amhr2 | AK020937 | Rbm16 |
| MMT00050524 | Amigo2 | AK002739 | Rbm43 |
| AK009268 | Amotl1 | MMT00044670 | Rbm9 |
| AW490056 | Amph | MMT00078489 | Rbms2 |
| NM_007446 | Amy1 | NM_019711 | Rbms2 |
| NM_009669 | Amy2 | NM_011254 | Rbp1 |
| ri|2510006G12|ZX00082G04||2434 | Anapc5 | AK004839 | Rbp4 |
| AK011976 | Angptl2 | AK010301 | Rbpms2 |
| MMT00067132 | Ankrd28 | ri|5730557L09|PX00006H03||2031 | Rc3h1 |
| AI428512 | Ankrd29 | MMT00017157 | Rcbtb2 |
| MMT00000051 | Ankrd32 | AK009709 | Rcl1 |
| AK020869 | Ankrd37 | AK017494 | Rcn1 |
| MMT00077997 | Ankrd40 | MMT00072793 | Rcor1 |
| MMT00046013 | Ankrd46 | AK030934 | Rcsd1 |
| MMT00078802 | Ankrd57 | AK014244 | Rdh10 |
| BC010633 | Anks1 | NM_153133 | Rdh9 |
| NM_009673 | Anxa5 | MMT00042720 | Reep1 |
| AK010342 | Anxa6 | U28168 | Reep5 |
| MMT00016047 | Aoah | MMT00050150 | Reps2 |
| NM_009675 | Aoc3 | MMT00042794 | Rerg |
| NM_009676 | Aox1 | MMT00078803 | Rergl |
| MMT00033154 | Ap1gbp1 | NM_022984 | Retn |
| AK005223 | Ap1s2 | NM_020509 | Retnla |
| NM_026193 | Ap4b1 | MMT00007494 | Retnlg |
| NM_021392 | Ap4m1 | AK002851 | Retsat |
| MMT00053308 | Apbb3 | AK045402 | Rexo4 |
| AB023957 | Apcdd1 | MMT00065798 | Rfc3 |
| MMT00078870 | Apln | AK011489 | Rfc5 |
| NM_009691 | Aplp2 | MMT00003516 | Rgma |
| NM_007385 | Apoc4 | BC003882 | Rgs4 |
| NM_177820 | Apol10b | NM_009063 | Rgs5 |
| AK010208 | Apol6 | AF011360 | Rgs7 |
| NM_007472 | Aqp1 | MMT00004546 | Rgs7bp |
| AK010267 | Arbp | MMT00000361 | Rgs7bp |
| mCT159326 | Arf6 | NM_007483 | Rhob |
| NM_023854 | Arfgap2 | AK014194 | Rhobtb1 |
| ENSMUST00000065496 | Arhgap20 | MMT00007674 | Rhobtb1 |
| MMT00038075 | Arhgap21 | MMT00071141 | Rhobtb2 |
| MMT00030465 | Arhgap24 | MMT00073956 | Rhou |
| MMT00039812 | Arhgef10 | MMT00016498 | Rims4 |
| AK009331 | Arhgef12 | MMT00081972 | Ripply3 |
| MMT00023383 | Arhgef15 | AJ237586 | Rmnd5a |
| MMT00027493 | Arid1b | NM_026616 | Rnaseh2c |
| MMT00010430 | Arl15 | ENSMUST00000022802 | Rnasen |
| MMT00004466 | Arl2bp | AK014754 | Rnaset2a |
| MMT00048204 | Arl4c | NM_028810 | Rnd3 |
| MMT00058570 | Armcx3 | MMT00042967 | Rnf144a |
| AI845493 | Armcx6 | MMT00062358 | Rnf152 |
| MMT00047708 | Arnt2 | MMT00044486 | Rnf157 |
| NM_007489 | Arntl | ENSMUST00000062286 | Rnf180 |
| AK003485 | Arpc3 | NM_021313 | Rnf25 |
| AK016257 | Art3 | NM_181405 | Rnpepl1 |
| NM_018830 | Asah2 | AK004723 | Robo4 |
| MMT00057142 | Asb5 | AK004265 | Rock1 |
| BC005552 | Asns | AK088934 | Rock2 |
| AK002859 | Aspa | NM_199201 | RP23-331L12.8 |
| NM_023066 | Asph | MMT00023569 | RP24-87L14.2 |
| NM_025711 | Aspn | AK005285 | Rpa3 |
| MMT00010709 | Atf4 | MMT00029657 | Rpap2 |
| AK003630 | Atf7 | NM_028003 | Rpap3 |
| MMT00034842 | Atg2a | NM_025683 | Rpe |
| MMT00057570 | Athl1 | ENSMUST00000061200 | Rpl10a |
| NM_007881 | Atn1 | MMT00023400 | Rpl13 |
| AK002432 | Atox1 | NM_025974 | Rpl14 |
| AI464465 | Atp10d | ENSMUST00000000012 | Rpl18 |
| AK009883 | Atp5g1 | ENSMUST00000070489 | Rpl19 |
| BC003810 | Atp6v1c2 | XM_143700 | Rpl21 |
| MMT00082394 | Atp6v1d | NM_022891 | Rpl23 |
| AK003446 | Atp6v1d | NM_011289 | Rpl27 |
| MMT00014440 | Atp6v1g1 | ENSMUST00000052054 | Rpl27a |
| XM_359372 | Atp6v1g2 | ENSMUST00000066762 | Rpl3 |
| MMS010258105 | Atp8a1 | AK003177 | Rpl34 |
| MMT00013986 | Atpaf2 | ENSMUST00000067519 | Rpl35a |
| MMT00013573 | Atpif1 | XM_358916 | Rpl35a |
| NM_009124 | Atxn1 | ri|2810431L15|ZX00083C16||462 | Rpl35a |
| NM_028139 | Atxn7l1 | MMT00011829 | Rpl36al |
| MMS010167043 | AU015536 | NM_011291 | Rpl7 |
| MMS010035139 | AU015601 | XM_357823 | Rpl7a |
| MMS010228876 | AU019559 | NM_012053 | Rpl8 |
| MMT00033694 | AU019823 | MMT00016709 | Rpn1 |
| MMS010231070 | AU021001 | NM_009085 | Rpo1-1 |
| MMT00008535 | AU021025 | NM_009088 | Rpo1-4 |
| MMS010114870 | AU021760 | AK010465 | Rprm |
| BC055790 | AU022252 | AK018703 | Rps13 |
| MMT00008824 | AU042671 | AK028135 | Rps15a |
| MMT00006949 | AW060763 | NM_011296 | Rps18 |
| MMT00051300 | AW121567 | AK010610 | Rps21 |
| MMS010211827 | AW552393 | MMT00016974 | Rps24 |
| MMS010028393 | AW556253 | AK002986 | Rps24 |
| MMS010238423 | AW556556 | MMT00019208 | Rps3 |
| AK038096 | AW742696 | ENSMUST00000064933 | Rps3a |
| MMT00043882 | B230337E12Rik | AK002235 | Rps5 |
| MMT00007478 | B230399E16Rik | MMT00040351 | Rpusd3 |
| AK018190 | B3galt1 | NM_009101 | Rras |
| AK046555 | B430007K19Rik | NM_025846 | Rras2 |
| mCT184336 | B430010I23Rik | NM_009103 | Rrm1 |
| AK046594 | B430119L08Rik | AK009288 | Rsu1 |
| AK080903 | B430203I24Rik | NM_013648 | Rtn2 |
| NM_177188 | B430209F14Rik | NM_022982 | Rtn4r |
| MMT00079947 | B430212C06Rik | MMT00080573 | Rtn4rl1 |
| MMT00046935 | B430316J06Rik | MMT00035704 | Rufy4 |
| NM_008080 | B4galnt1 | D14636 | Runx2 |
| MMT00010923 | B830045N13Rik | AK014130 | Rwdd3 |
| AK041916 | B930012P20Rik | NM_009112 | S100a10 |
| BC050212 | B930053N05Rik | AK002755 | S100a13 |
| AK020668 | B930094E09Rik | NM_023637 | Sars2 |
| AK047590 | B930095M22Rik | AK002531 | Sat1 |
| AK041049 | Bace1 | AK003459 | Sbsn |
| AK010765 | Bag4 | MMT00014959 | Sbsn |
| MMT00009668 | Baiap2 | AB016248 | Sc5d |
| AK011545 | Basp1 | NM_009127 | Scd1 |
| MMS010209062 | Basp1 | MMT00023269 | Scml4 |
| BB018844 | BB018844 | L42336 | Scn2a1 |
| MMT00036408 | BB114814 | AK003240 | Scn3b |
| MMS010154725 | BB124184 | MMT00010120 | Scn5a |
| AK083592 | BB217526 | AK004093 | Scn7a |
| BC004728 | BC004728 | AW491478 | Scn8a |
| MMT00055566 | BC013529 | ENSMUST00000005770 | Scnm1 |
| MMT00077611 | BC017647 | MMT00041518 | Sctr |
| MMT00051004 | BC020535 | NM_007926 | Scye1 |
| MMT00078550 | BC021395 | NM_009143 | Sdf2 |
| MMT00013388 | BC021614 | MMT00064340 | Sdhc |
| MMT00011758 | BC022687 | MMT00081436 | Sdpr |
| MMT00033582 | BC025076 | AK014487 | Sdsl |
| MMT00056571 | BC026585 | NM_019951 | Sec11a |
| mCT172500 | BC029722 | AK007641 | Sec11c |
| AK081366 | BC031353 | AK017615 | Sec61a2 |
| MMT00005265 | BC031353 | AF063095 | Sel1l |
| ri|9030204A07|PX00060F20||1343 | BC033915 | M32032 | Selenbp1 |
| MMT00003982 | BC034069 | NM_019414 | Selenbp2 |
| MMT00063747 | BC034902 | AK014535 | Sema3d |
| MMT00062408 | BC035537 | mCT175269 | Sema3e |
| MMT00042103 | BC037034 | AK004390 | Sema6d |
| MMT00020685 | BC042782 | BE334059 | Senp6 |
| MMT00008367 | BC046331 | NM_175400 | Sephs1 |
| NM_198861 | BC046404 | MMT00066228 | Sepsecs |
| MMT00042097 | BC055004 | MMT00062126 | Sepw1 |
| MMT00004227 | BC055107 | MMT00064849 | Sepx1 |
| MMT00078435 | BC057079 | MMT00061602 | Serinc5 |
| BC062258 | BC062258 | MMT00033772 | Serpina3b |
| BC051485 | BC064078 | NM_008458 | Serpina3c |
| NM_020486 | Bcam | MMT00033061 | Serpina3h |
| ri|1810008O06|R000022G09||1804 | Bcat2 | D00725 | Serpina3k |
| MMT00076328 | Bche | NM_009253 | Serpina3m |
| L47335 | Bckdha | AK003220 | Serpinb3b |
| L16992 | Bckdhb | MMT00077724 | Sertad4 |
| MMT00058264 | Bcl2 | AK005322 | Sestd1 |
| MMT00071840 | Bcl2l13 | MMT00065759 | Sestd1 |
| NM_009744 | Bcl6 | MMT00045695 | Setbp1 |
| NM_007528 | Bcl6b | MMT00072934 | Setd3 |
| NM_030256 | Bcl9l | MMT00012970 | Setd7 |
| AK017213 | Bcr | NM_021286 | Sez6 |
| ri|0610030E24|R000004A08||1499 | Bet1 | AK012009 | Sf3b2 |
| NM_021560 | Bhlhb5 | AK019095 | Sfi1 |
| AK003456 | Bicd2 | AK008943 | Sfrp1 |
| NM_007566 | Birc6 | MMT00046045 | Sfrp5 |
| NM_016916 | Blcap | AK017471 | Sft2d1 |
| MMT00078825 | Bloc1s2 | AK012650 | Sfxn1 |
| NM_007553 | Bmp2 | AK046372 | Sfxn1 |
| MMT00001308 | Bmp3 | NM_053198 | Sfxn4 |
| NM_007554 | Bmp4 | NM_011891 | Sgcd |
| NM_007556 | Bmp6 | MMT00009951 | Sgce |
| U88064 | Bnc1 | AK014022 | Sgip1 |
| MMT00062155 | Bnc2 | NM_030750 | Sgpp1 |
| AF041054 | Bnip3 | NM_024499 | Sgta |
| AK019037 | Bola3 | NM_018825 | Sh2b2 |
| NM_025631 | Bpil1 | NM_008507 | Sh2b3 |
| MMT00027519 | Bptf | NM_013781 | Sh2d3c |
| AK014475 | Brd8 | MMT00040030 | Sh2d4a |
| AK011889 | Brp44 | ri|5430401P15|PX00022K17||1812 | Sh3bgrl2 |
| AK014435 | Brp44l | MMT00007995 | Sh3bp4 |
| MMT00032102 | Brpf3 | NM_019464 | Sh3glb1 |
| AF314173 | Brunol4 | BC024477 | Sh3glb2 |
| AK004634 | Bscl2 | MMT00040892 | Sh3tc2 |
| AK002942 | Btbd14a | NM_021423 | Shank3 |
| NM_007568 | Btc | MMT00016883 | Shroom4 |
| NM_007570 | Btg2 | D11374 | Sipa1 |
| MMT00035542 | Btnl9 | AK012129 | Sipa1l3 |
| AW146087 | Btnl9 | AK002609 | Sirt5 |
| MMT00035285 | Btrc | MMT00075427 | Sirt5 |
| MMT00003620 | C030006K11Rik | D83146 | Six5 |
| AK048212 | C130040J23Rik | NM_011386 | Skil |
| AK048273 | C130045F17Rik | AK018608 | Skil |
| AK048402 | C130057M05Rik | MMT00063500 | Slain2 |
| MMT00017549 | C130074G19Rik | NM_030683 | Slc14a2 |
| XM_139575 | C1ql4 | NM_009197 | Slc16a2 |
| MMT00042470 | C1qtnf1 | AK014522 | Slc17a5 |
| MMT00058544 | C1qtnf5 | AF330257 | Slc1a3 |
| NM_145613 | C1qtnf5 | AK050752 | Slc1a3 |
| AK076300 | C230007H23Rik | NM_009201 | Slc1a5 |
| AK082309 | C230036F13Rik | MMT00028007 | Slc20a1 |
| AK082631 | C230071I02Rik | MMT00022494 | Slc22a18 |
| AK049070 | C230096K16Rik | MMT00016181 | Slc22a23 |
| AK049648 | C530014P21Rik | NM_011395 | Slc22a3 |
| MMT00009796 | C530044N13Rik | BC005742 | Slc24a3 |
| MMT00027045 | C630004H02Rik | AK008038 | Slc25a10 |
| MMS010263616 | C76434 | NM_015829 | Slc25a13 |
| MMS010033677 | C76450 | ri|2900089E13|ZX00070K11||2269 | Slc25a19 |
| AK040847 | C76686 | MMT00026271 | Slc25a23 |
| MMS010262321 | C77135 | AK017037 | Slc25a26 |
| MMS010265898 | C77368 | MMT00076944 | Slc25a35 |
| AK082577 | C77714 | NM_026165 | Slc25a46 |
| MMS010037160 | C80012 | NM_011867 | Slc26a4 |
| AK040036 | C80446 | NM_011977 | Slc27a1 |
| AK080316 | C81268 | ri|2610030J19|ZX00034A12||1991 | Slc27a3 |
| AK078747 | C85445 | NM_178934 | Slc2a12 |
| AK085124 | C86807 | NM_009204 | Slc2a4 |
| AI465472 | C920006O11Rik | NM_019488 | Slc2a8 |
| NM_022021 | Cables1 | BC006076 | Slc2a9 |
| AK010631 | Cabp4 | MMT00071411 | Slc30a2 |
| NM_009781 | Cacna1c | ri|1810059J10|ZX00051C02||2022 | Slc30a7 |
| AK018426 | Cacna1d | NM_011402 | Slc34a2 |
| NM_009784 | Cacna2d1 | AK003377 | Slc35b2 |
| AK014145 | Cacnb4 | MMT00035782 | Slc39a6 |
| NM_012061 | Cadps | MMT00029681 | Slc41a3 |
| AK014755 | Cald1 | AK011417 | Slc43a1 |
| NM_007595 | Camk2b | MMT00062173 | Slc44a3 |
| AK013788 | Camk2n2 | MMT00016253 | Slc45a3 |
| MMT00034809 | Camta1 | MMT00046642 | Slc4a8 |
| AJ289241 | Capn12 | ri|6720408E06|PX00059I21||1255 | Slc6a13 |
| MMT00053553 | Capn5 | MMT00056236 | Slc7a1 |
| NM_009799 | Car1 | NM_017394 | Slc7a10 |
| AK003671 | Car3 | NM_023055 | Slc9a3r2 |
| AK040686 | Car3 | AK028623 | Slco2b1 |
| MMT00042726 | Car7 | MMT00034802 | Slco3a1 |
| MMT00065318 | Card10 | MMT00027658 | Slco5a1 |
| NM_025821 | Carhsp1 | AF144628 | Slit2 |
| MMT00023915 | Casc4 | NM_011414 | Slpi |
| NM_007611 | Casp7 | MMT00030306 | Smarca2 |
| MMT00041835 | Casz1 | NM_009211 | Smarcc1 |
| NM_009804 | Cat | NM_198160 | Smarcc2 |
| AB029930 | Cav1 | NM_022316 | Smoc1 |
| AK004663 | Cav2 | NM_021491 | Smpd3 |
| MMT00071071 | Cbfa2t3 | MMT00007093 | Smtnl2 |
| NM_009824 | Cbfa2t3 | MMT00049660 | Smyd2 |
| NM_007625 | Cbx4 | MMT00065715 | Snca |
| ri|5730509K17|PX00103A13||1674 | Cc2d2a | NM_026408 | Sncaip |
| MMT00077881 | Ccbl2 | MMT00033656 | Snf1lk2 |
| NM_021609 | Ccbp2 | AK013035 | Snn |
| MMT00072928 | Ccdc102a | MMT00026438 | Snn |
| MMT00010855 | Ccdc112 | MMT00034001 | Snrk |
| AK008760 | Ccdc69 | ENSMUST00000063243 | Snrp70 |
| AK011256 | Ccdc80 | AK019462 | Snrpe |
| BC002299 | Ccdc88c | ENSMUST00000053575 | Snrpg |
| AK018089 | Ccdc90a | MMT00005397 | Snrpg |
| NM_027796 | Ccdc98 | MMT00037827 | Snrpn |
| MMT00067646 | Ccl24 | S62288 | Snrpn |
| NM_021443 | Ccl8 | NM_009228 | Snta1 |
| NM_009829 | Ccnd2 | BC003748 | Sntb1 |
| U43844 | Ccnd3 | NM_016667 | Sntb1 |
| U82832 | Ccnd3-ps | MMT00077727 | Snurf |
| Z37110 | Ccng1 | NM_019727 | Snx1 |
| ri|6330408H09|PX00008G23||1979 | Ccnh | AK017516 | Snx21 |
| AK013634 | Ccnt2 | BC002242 | Snx5 |
| AF274883 | Cd163 | MMT00013676 | Soat1 |
| AK002582 | Cd1d1 | U38261 | Sod3 |
| NM_013486 | Cd2 | AK010043 | Sorbs1 |
| AF004023 | Cd200 | U58883 | Sorbs1 |
| MMT00029261 | Cd209a | U58883 | Sorbs1 |
| NM_130903 | Cd209c | AK013519 | Sorl1 |
| MMT00067592 | Cd209c | D49473 | Sox17 |
| MMT00070727 | Cd209d | BC006612 | Sox18 |
| NM_026956 | Cd209f | AK054386 | Sox4 |
| MMT00028860 | Cd209g | MMT00017123 | Sox6 |
| MMT00043862 | Cd226 | NM_011446 | Sox7 |
| AK017904 | Cd247 | AF062567 | Sp3 |
| NM_019421 | Cd320 | MMT00024462 | Spag9 |
| ri|A930023P06|PX00066B22||1477 | Cd59a | MMT00040204 | Spata2 |
| NM_007653 | Cd63 | AK007336 | Spcs1 |
| NM_007656 | Cd82 | MMT00049815 | Spna2 |
| MMT00038444 | Cd9 | MMS010206249 | Spna2 |
| MMT00002201 | Cd99l2 | NM_009260 | Spnb2 |
| MMT00018455 | Cdadc1 | MMT00015514 | Spon1 |
| AK007896 | Cdc42ep1 | NM_025287 | Spop |
| MMT00029168 | Cdc42ep2 | NM_011467 | Spr |
| AK008154 | Cdc42ep5 | MMT00051722 | Spred3 |
| MMT00051624 | Cdc42se2 | AW319546 | Sprr2a |
| MMT00067952 | Cdh11 | MMT00063725 | Spryd3 |
| MMT00062044 | Cdh11 | MMT00055332 | Srd5a1 |
| AK011871 | Cdk4 | NM_011157 | Srgn |
| U22399 | Cdkn1c | NM_011481 | Srms |
| U19596 | Cdkn2c | NM_009275 | Srprb |
| NM_007672 | Cdr2 | MMT00042352 | Ssbp3 |
| MMT00057904 | Cdsn | MMT00036522 | Ssbp4 |
| AK048941 | Cdv3 | NM_198109 | Ssh1 |
| AK015452 | Cdyl2 | MMT00062061 | Ssh2 |
| MMT00000475 | Cdyl2 | NM_010656 | Sspn |
| AK048664 | Cecr2 | AK003545 | St13 |
| AK013061 | Cecr2 | NM_009176 | St3gal3 |
| NM_207223 | Centb5 | AK008543 | St3gal4 |
| MMT00059656 | Cep63 | NM_011375 | St3gal5 |
| NM_028815 | Cep97 | NM_009175 | St6gal1 |
| MMT00031586 | Ces2 | NM_016853 | Stac |
| NM_053200 | Ces3 | AK020229 | Stambpl1 |
| NM_013459 | Cfd | MMT00077271 | Stard13 |
| MMT00009666 | Chchd6 | AK087617 | Stard9 |
| AK011183 | Chd3 | NM_025303 | Stau2 |
| ENSMUST00000039267 | Chd7 | AK010437 | Steap1 |
| MMT00074856 | Chd7 | AK003905 | Steap4 |
| mCT153553 | Chd9 | MMT00049964 | Stim1 |
| AK083699 | Chml | NM_011492 | Stk11 |
| AK008205 | Chmp4b | MMS010043618 | Stt3b |
| mCT120360 | Chmp6 | NM_172675 | Stx16 |
| ri|0710001E19|R000005K11||2055 | Chn1 | MMT00060118 | Stx17 |
| AK007964 | Chpt1 | NM_011505 | Stxbp4 |
| NM_031258 | Chrdl1 | NM_177119 | Stxbp4 |
| AF204689 | Chrna5 | MMT00035951 | Stxbp6 |
| NM_009601 | Chrnb1 | NM_019637 | Styx |
| NM_023850 | Chst1 | NM_032400 | Sucnr1 |
| NM_021528 | Chst12 | AK008108 | Sulf2 |
| MMT00076583 | Chsy1 | L02331 | Sult1a1 |
| MMT00022637 | Cib2 | MMT00054344 | Sult4a1 |
| MMT00071047 | Cisd2 | NM_020564 | Sult5a1 |
| NM_009895 | Cish | mCT13653 | Sumf2 |
| Y15163 | Cited2 | MMT00029781 | Supv3l1 |
| MMT00012015 | Clasp1 | MMS010123914 | Susd2 |
| mCT167855 | Clasp2 | NM_025932 | Syap1 |
| MMT00020687 | Cldn22 | ENSMUST00000040580 | Syde1 |
| NM_009131 | Clec11a | XM_359260 | Syde1 |
| MMT00030377 | Clint1 | ENSMUST00000023882 | Sympk |
| MMT00031345 | Clip4 | NM_013680 | Syn1 |
| MMT00046744 | Clip4 | MMT00061063 | Syne2 |
| NM_007715 | Clock | MMT00062010 | Syne2 |
| NM_022319 | Clstn2 | NM_025292 | Synj2bp |
| AK009844 | Cltb | MMT00072592 | Synpo2 |
| AK002741 | Clybl | NM_009305 | Syp |
| AK009374 | Cmbl | ri|B230112P13|PX00068I12||1176 | Syt7 |
| AF188504 | Cmtm7 | AV294988 | Tacc2 |
| NM_023149 | Cndp2 | NM_021314 | Tacc2 |
| MMT00074806 | Cnksr3 | AK082703 | Tacc2 |
| BC026992 | Cnot4 | BC016522 | Taok1 |
| MMT00017496 | Cobll1 | MMT00001741 | Taok2 |
| NM_029746 | Cog2 | MMT00020745 | Tarsl2 |
| MMT00015518 | Col8a2 | MMT00016998 | Tbc1d14 |
| NM_021538 | Cope | MMT00071059 | Tbc1d17 |
| NM_019877 | Copz2 | NM_144517 | Tbc1d19 |
| AK020647 | Coq10b | NM_018775 | Tbc1d8 |
| ri|5930427M12|PX00055H22||1521 | Coq6 | AK014817 | Tbc1d8b |
| MMT00079185 | Cox18 | NM_027758 | Tbc1d9 |
| NM_007754 | Cpd | ri|E130107N23|PX00091H11||1437 | Tbcel |
| MMT00046700 | Cpeb3 | NM_198100 | Tbkbp1 |
| ri|3322401K10|PX00010E04||2295 | Cpne2 | MMT00017542 | Tcea3 |
| MMT00006816 | Cpne5 | NM_025703 | Tceal8 |
| NM_007760 | Crat | NM_013736 | Tceb3 |
| ENSMUST00000050372 | Crb2 | MMT00004938 | Tcf15 |
| BE943969 | Creb5 | AK021120 | Tcf4 |
| MMT00038931 | Creld1 | AK018098 | Tcf7l2 |
| MMT00026635 | Crim1 | MMT00052791 | Tcrb-V8.3 |
| AK012068 | Crip1 | NM_133986 | Tcta |
| ri|2310058B21|ZX00055C15||1846 | Crls1 | NM_026486 | Tctn2 |
| AK012016 | Crtc3 | AK009518 | Tef |
| BC006077 | Cry2 | NM_013690 | Tek |
| NM_009964 | Cryab | MMT00035903 | Tenc1 |
| MMT00064112 | Csde1 | BC003808 | Tes |
| AI413667 | Csgalnact1 | MMT00026938 | Tex2 |
| XM_358797 | Csta | BB283790 | Tfdp2 |
| AK013115 | Ctdspl | NM_009365 | Tgfb1i1 |
| AK003674 | Cthrc1 | NM_009370 | Tgfbr1 |
| AK004623 | Ctnna1 | MMT00005229 | Tgif1 |
| AK008288 | Ctnna1 | AK008975 | Thap3 |
| NM_018737 | Ctps2 | AK084295 | Thap6 |
| NM_007801 | Ctsh | NM_009378 | Thbd |
| NM_010160 | Cugbp2 | NM_025416 | Them5 |
| NM_009142 | Cx3cl1 | NM_001001297 | Thnsl1 |
| NM_013655 | Cxcl12 | BE995557 | Thrb |
| NM_007722 | Cxcr7 | AI987838 | Thrb |
| MMT00031186 | Cxx1a | NM_009381 | Thrsp |
| AK003646 | Cxx1b | MMT00071943 | Thsd7b |
| MMT00072795 | Cxx1c | NM_009384 | Tiam1 |
| BC056184 | Cxxc4 | XM_358883 | Tiam1 |
| MMT00026453 | Cyb5r3 | MMT00035358 | Tigd5 |
| ri|2310004G04|ZX00038H19||1243 | Cybasc3 | NM_011595 | Timp3 |
| U03283 | Cyp1b1 | AF282730 | Timp4 |
| U03283 | Cyp1b1 | MMT00035506 | Tinag |
| MMT00015687 | Cyp27a1 | NM_009386 | Tjp1 |
| AF047725 | Cyp2c38 | MMT00037403 | Tjp3 |
| NM_019823 | Cyp2d22 | ENSMUST00000002025 | Tktl2 |
| NM_021282 | Cyp2e1 | BC005702 | Tlcd1 |
| MMT00022093 | Cyp2j11 | NM_009390 | Tll1 |
| NM_022434 | Cyp4f14 | BB269798 | Tln2 |
| NM_024444 | Cyp4f18 | MMS010240346 | Tln2 |
| AK004724 | Cyp4v3 | NM_016928 | Tlr5 |
| NM_007825 | Cyp7b1 | MMT00026255 | Tm4sf1 |
| MMT00050222 | Cytl1 | BC004752 | Tmbim1 |
| MMS010230086 | D030011O10Rik | AK006207 | Tmbim4 |
| AK013560 | D0Kist2 | NM_026211 | Tmed9 |
| AK017596 | D10Bwg1070e | MMT00011091 | Tmeff1 |
| MMS010115007 | D10Ertd638e | MMT00008388 | Tmem109 |
| MMT00012826 | D10Ertd641e | MMT00076568 | Tmem120a |
| MMS010114485 | D11Ertd726e | NM_025480 | Tmem128 |
| MMS010239906 | D12Ertd123e | AK009263 | Tmem134 |
| AK003665 | D12Ertd647e | AK013269 | Tmem135 |
| AK079388 | D130012G24Rik | AI450380 | Tmem140 |
| MMT00029645 | D130017N08Rik | AK004380 | Tmem141 |
| MMS010210343 | D13Ertd787e | MMT00051893 | Tmem143 |
| MMT00003646 | D14Ertd500e | AK004282 | Tmem144 |
| MMS010000502 | D15Wsu126e | MMT00040004 | Tmem14a |
| MMS010089000 | D17Ertd165e | MMT00073923 | Tmem164 |
| AK079772 | D18Ertd232e | MMT00070074 | Tmem16k |
| MMT00044963 | D18Ertd734e | ri|5730411F04|PX00002B15||1057 | Tmem176a |
| AK084413 | D230040J21Rik | MMT00039789 | Tmem182 |
| AK052079 | D230044B12Rik | AB041601 | Tmem191c |
| AK052185 | D330004O07Rik | NM_178577 | Tmem205 |
| MMT00050788 | D330017J20Rik | MMT00043620 | Tmem22 |
| MMT00063388 | D330027G24Rik | MMT00081812 | Tmem30b |
| MMT00018145 | D330045A20Rik | MMT00059484 | Tmem41a |
| BE948923 | D3Bwg0562e | MMT00026397 | Tmem43 |
| MMS010099678 | D3Ertd452e | MMT00058639 | Tmem45b |
| MMT00019364 | D430015B01Rik | MMT00015821 | Tmem56 |
| MMT00005428 | D430019H16Rik | NM_027415 | Tmem70 |
| MMT00017163 | D4Wsu53e | NM_021793 | Tmem8 |
| AK021303 | D530031A16Rik | AK013215 | Tmem87b |
| MMS010176323 | D5Ertd255e | AK018332 | Tmem98 |
| AK077449 | D5Ertd505e | MMT00027275 | Tmlhe |
| MMT00045978 | D5Ertd579e | AY033513 | Tmlhe |
| AK014530 | D5Wsu178e | NM_021883 | Tmod1 |
| AK085463 | D630030B08Rik | NM_030709 | Tmprss5 |
| AI482473 | D730040F13Rik | NM_013749 | Tnfrsf12a |
| NM_176907 | D830016O14Rik | AI117633 | Tnik |
| mCT184066 | D830026I12Rik | NM_021327 | Tnip1 |
| MMS010056239 | D8Ertd158e | MMT00027041 | Tnip3 |
| AK086137 | D930007M16Rik | NM_011618 | Tnnt1 |
| MMT00047801 | D930026N18Rik | MMT00049628 | Tnrc6c |
| AK086649 | D930043N17Rik | MMT00055301 | Tns1 |
| MMT00076560 | D9Ertd402e | AK003780 | Tns1 |
| AK083548 | D9Wsu74e | NM_027884 | Tns1 |
| AK038263 | D9Wsu90e | NM_009427 | Tob1 |
| AK009291 | Dab2ip | AJ297743 | Tor1b |
| NM_021532 | Dact1 | MMT00019069 | Tox2 |
| AB018002 | Dapk2 | AK005456 | Tpd52 |
| NM_172644 | Dars2 | MMT00060338 | Tph2 |
| AK020692 | Dbi | NM_013861 | Tpk1 |
| MMT00027796 | Dbndd2 | MMT00019410 | Tppp |
| MMT00006929 | Dbp | NM_026481 | Tppp3 |
| NM_016974 | Dbp | AK004777 | Tprgl |
| NM_010022 | Dbt | AK005187 | Tra2a |
| NM_010023 | Dci | MMT00009300 | Traf6 |
| NM_019978 | Dclk1 | MMT00063341 | Trak1 |
| MMT00019833 | Dclk3 | AY029764 | Tram1 |
| AK004384 | Dcp1a | NM_026508 | Trap1 |
| AK020737 | Dcst1 | AK015839 | Trappc6a |
| MMT00010922 | Dcst2 | AK020026 | Trappc6b |
| BG093780 | Ddef1 | NM_133977 | Trf |
| U92478 | Ddef1 | MMT00032248 | Trhde |
| AK017926 | Ddit4 | MMT00020695 | Trib3 |
| MMT00055725 | Ddost | ri|9130006M08|PX00026C19||1741 | Trim16 |
| L57509 | Ddr1 | NM_030706 | Trim2 |
| MMT00047600 | Ddx24 | MMT00064791 | Trim24 |
| MMT00057734 | Ddx27 | AK019165 | Trim3 |
| MMS010098433 | Defb12 | MMT00010101 | Trim46 |
| mCT51966 | Defb23 | MMT00073577 | Trim62 |
| AJ575425 | Defb39 | NM_013835 | Trove2 |
| MMT00015041 | Degs1 | MMT00000664 | Trp53rk |
| MMT00021997 | Depdc2 | NM_019510 | Trpc3 |
| MMT00012322 | Depdc6 | MMT00030936 | Trpv1 |
| NM_172733 | Dera | MMT00069850 | Trub2 |
| AF078752 | Dgat1 | NM_011647 | Tsc2 |
| NM_026384 | Dgat2 | AK007760 | Tsc22d2 |
| MMT00046414 | Dgkh | AK014519 | Tshr |
| MMT00027978 | Dhdh | NM_021367 | Tslp |
| NM_030686 | Dhrs4 | MMT00011453 | Tspan12 |
| Y15910 | Diap2 | NM_025359 | Tspan13 |
| ENSMUST00000037764 | Dido1 | MMT00005654 | Tspan17 |
| ENSMUST00000029089 | Dido1 | MMT00059781 | Tspan2 |
| NM_010050 | Dio2 | AK017989 | Tspan32 |
| MMT00017999 | Dirc2 | AK004954 | Tspan33 |
| AK004853 | Dkk3 | NM_019571 | Tspan5 |
| MMT00058364 | Dlc1 | MMT00075556 | Tspan7 |
| ri|2610028K08|ZX00034E09||2223 | Dld | NM_009775 | Tspo |
| AK033078 | Dleu2 | NM_024477 | Ttc28 |
| NM_019454 | Dll4 | NM_025736 | Ttc35 |
| U79738 | Dlx3 | AK004882 | Ttpa |
| Z38015 | Dmpk | MMT00003624 | Ttyh1 |
| MMT00023412 | Dmrt2 | AK019322 | Ttyh3 |
| NM_010058 | Dmwd | M13445 | Tuba1a |
| MMT00010359 | Dnajb11 | MMT00077987 | Tuba1b |
| NM_013888 | Dnajc12 | U89398 | Tuba1b |
| NM_007870 | Dnase1l3 | M19413 | Tuba-rs1 |
| AK017170 | Dock11 | AK010786 | Tubb2b |
| MMT00054006 | Dock11 | NM_023716 | Tubb2b |
| ENSMUST00000039135 | Dock5 | NM_134024 | Tubg1 |
| NM_172811 | Dock5 | NM_011656 | Tuft1 |
| ENSMUST00000034728 | Dock6 | AK009482 | Tulp3 |
| BC009134 | Dock9 | mCT185987 | Tulp4 |
| MMT00016717 | Dok1 | AK011861 | Twist2 |
| NM_029761 | Dok5 | AK013150 | Txndc1 |
| NM_020329 | Dolpp1 | NM_029572 | Txndc4 |
| MMT00054562 | Dpy19l4 | MMT00043714 | Txnl4b |
| MMT00021230 | Dpy30 | ri|2210410N19|ZX00054E04||869 | U2af1 |
| MMT00054055 | Dst | MMT00043516 | Uap1 |
| AK019477 | Dtna | NM_026872 | Ubap2 |
| AK012137 | Dtx2 | AK011954 | Ube2b |
| MMT00034939 | Dtx4 | XM_356995 | Ube2d2 |
| ri|5031436O03|PX00037E04||1830 | Dusp3 | NM_009459 | Ube2h |
| NM_026268 | Dusp6 | MMT00066235 | Ube2i |
| NM_008748 | Dusp8 | MMT00056281 | Ube3b |
| BC004738 | Dusp9 | ENSMUST00000065023 | Ubxd6 |
| AK018993 | E030003E18Rik | BC004016 | Uck2 |
| mCT185086 | E030003E18Rik | NM_009463 | Ucp1 |
| NM_183160 | E030010A14Rik | AY227196 | Ugt1a5 |
| mCT134405 | E030010N08Rik | AB019577 | Ulk2 |
| AK086966 | E030016H06Rik | AI591459 | Ulk2 |
| AK018512 | E030024N20Rik | NM_011676 | Unc119 |
| AK087092 | E030026E10Rik | MMT00057878 | Unc119b |
| AW494428 | E130014H10Rik | MMT00081171 | Unc45b |
| AK021363 | E130101A01Rik | MMT00069942 | Unc84a |
| AK053684 | E130202H07Rik | NM_011677 | Ung |
| MMS010238581 | E130317F20Rik | MMT00019068 | Uprt |
| AK078540 | E230020D15Rik | MMT00034710 | Uqcc |
| AF032131 | E2f6 | MMT00060700 | Use1 |
| MMT00011873 | E330021D16Rik | MMT00082121 | Ushbp1 |
| MMT00000335 | E330033B04Rik | NM_198421 | Usp49 |
| AK088440 | E430016L07Rik | ENSMUST00000019966 | Utrn |
| NM_007894 | Ear1 | NM_011682 | Utrn |
| NM_053112 | Ear10 | Y12229 | Utrn |
| MMT00048836 | Ear11 | NM_030736 | V1rd14 |
| MMT00052170 | Ear2 | MMT00072484 | V1rd14 |
| NM_007895 | Ear2 | MMT00057805 | V1rg6 |
| U72031 | Ear2 | NM_175137 | Vars2 |
| NM_017388 | Ear3 | MMT00026101 | Vash1 |
| MMT00060727 | Ear6 | MMT00017787 | Vegfa |
| AK036716 | Ebf1 | NM_011697 | Vegfb |
| NM_007897 | Ebf1 | NM_009506 | Vegfc |
| AK014058 | Ebf3 | MMT00065511 | Vezf1 |
| MMT00000642 | Ebf3 | MMT00069193 | Vipr2 |
| ENSMUST00000065252 | Ech1 | MMT00067875 | Vit |
| AF030343 | Ech1 | mCT181184 | Vmn2r17 |
| BC002178 | Echs1 | NM_011704 | Vnn1 |
| MMT00022737 | Eda2r | NM_011979 | Vnn3 |
| AB042828 | Edem1 | MMT00057849 | Vps13c |
| MMT00044340 | Eef2k | AK080288 | Vps13d |
| NM_026189 | Eepd1 | NM_020518 | Vsig2 |
| MMT00048340 | Efemp1 | NM_021387 | Vstm2b |
| MMS010170879 | Efna5 | AK003692 | Vti1a |
| NM_010109 | Efna5 | MMT00008034 | Vwa1 |
| MMT00048514 | EG225058 | NM_031877 | Wasf1 |
| MMT00053900 | EG225609 | AK017717 | Wbp5 |
| XM_136255 | EG226472 | ri|2310012I10|ZX00039K15||1396 | Wdr18 |
| XM_124146 | EG229746 | MMT00010891 | Wdr42a |
| XM_145468 | EG233075 | MMT00075877 | Wdr67 |
| XM_146296 | EG234159 | AK011714 | Wdr68 |
| mCT156508 | EG236893 | NM_198413 | Wdr70 |
| XM_140042 | EG240110 | MMT00040358 | Wfdc12 |
| XM_136732 | EG241053 | AK086344 | Whsc1 |
| XM_144599 | EG243302 | BB524893 | Whsc1 |
| XM_141707 | EG245391 | MMS010267436 | Wiz |
| MMT00072391 | EG245651 | NM_009519 | Wnt11 |
| mCT123088 | EG268795 | NM_009525 | Wnt5b |
| mCT14991 | EG268809 | BC006733 | Wwc1 |
| XM_194054 | EG329521 | MMT00046993 | Wwc2 |
| XM_290030 | EG333830 | AK009983 | Wwtr1 |
| mCT52265 | EG382371 | NM_013753 | X99384 |
| MMT00077018 | EG383080 | AK012549 | Xlr |
| XM_357274 | EG383815 | MMT00058326 | Xrcc6bp1 |
| XM_357471 | EG384179 | AK012787 | Yaf2 |
| XM_357690 | EG384525 | AK009405 | Yipf7 |
| XM_354686 | EG432681 | AK007486 | Ykt6 |
| ENSMUST00000058471 | EG432768 | AV312153 | Yme1l1 |
| MMT00081043 | EG432838 | AK010201 | Ypel5 |
| XM_131957 | EG433865 | NM_018871 | Ywhag |
| XM_356174 | EG434428 | AK012540 | Ywhaq |
| XM_125295 | EG434616 | AK007522 | Zadh1 |
| ENSMUST00000061472 | EG436332 | AK009517 | Zbtb4 |
| XM_359166 | EG545172 | AK020335 | Zbtb4 |
| XM_193374 | EG546052 | MMT00056689 | Zbtb46 |
| MMS010038901 | EG622304 | ri|9430083K24|PX00110H03||1557 | Zbtb5 |
| XM_122139 | EG622589 | NM_009565 | Zbtb7b |
| AK032775 | EG623172 | NM_026083 | Zc3h13 |
| XM_356686 | EG623174 | MMT00032711 | Zcchc18 |
| XM_356883 | EG623519 | MMT00022386 | Zcchc9 |
| MMT00081955 | EG625219 | AK017566 | Zdhhc2 |
| XM_356892 | EG625835 | NM_027476 | Zdhhc24 |
| XM_112472 | EG627798 | MMT00005394 | Zeb1 |
| MMT00078234 | EG627967 | NM_148926 | Zfand3 |
| MMT00053176 | EG629643 | NM_009551 | Zfand5 |
| MMT00079522 | EG629959 | AF060246 | Zfp106 |
| XM_358227 | EG633570 | MMT00069382 | Zfp14 |
| MMT00020356 | EG634650 | NM_145547 | Zfp189 |
| MMT00034132 | EG636544 | MMT00070314 | Zfp354b |
| MMT00047478 | EG638532 | MMT00019687 | Zfp367 |
| mCT9620 | EG640268 | NM_013866 | Zfp385a |
| XM_356977 | EG666111 | AF188609 | Zfp423 |
| AK087483 | EG666892 | AK011173 | Zfp553 |
| MMT00067322 | EG667148 | AK012367 | Zfp566 |
| XM_124905 | EG668668 | AK012894 | Zfp618 |
| AV329975 | Egfl7 | BC059079 | Zfp618 |
| BC006903 | Egln1 | MMT00053689 | Zfp664 |
| BC022961 | Egln3 | MMT00041957 | Zfp697 |
| BC009093 | Egr2 | MMT00040002 | Zfp703 |
| MMT00045371 | Ehd1 | MMT00057434 | Zfp820 |
| BC027084 | Ehd2 | BE945249 | Zfyve9 |
| MMT00001636 | Ehd2 | BC046341 | Zfyve9 |
| AK004867 | Ehhadh | AK007902 | Zhx3 |
| MMT00037652 | Eif2s2 | NM_009517 | Zmat3 |
| AK004664 | Eif3k | AK004445 | Znrf2 |
| MMT00050531 | Eif4b | MMT00058328 | Znrf2 |
| AK013033 | Eif4ebp1 | AK018382 | Zzef1 |
| MMS010247088 | Eif4ebp1 | AF061179 |  |
| NM_010124 | Eif4ebp2 | AK009685 |  |
| AK012709 | Eif4ebp2 | AK052554 |  |
| NM_015779 | Ela2 | AK086553 |  |
| NM_023479 | Elac2 | BC059074 |  |
| AF277093 | Elovl4 | BE988569 |  |
| AK004319 | Elovl5 | MMS010030254 |  |
| ri|1110033N21|R000017L15||1636 | Eltd1 | MMS010123503 |  |
| MMT00023589 | Emilin2 | MMS010146276 |  |
| AK019611 | Eml4 | MMS010153520 |  |
| AF346627 | Emp2 | MMS010179677 |  |
| NM_007932 | Eng | MMS010267066 |  |
| NM_007934 | Enpep | MMT00000825 |  |
| NM_008813 | Enpp1 | MMT00002151 |  |
| ENSMUST00000067817 | ENSMUSG00000054651 | MMT00003764 |  |
| MMT00012862 | ENSMUSG00000071036 | MMT00011281 |  |
| MMT00036027 | ENSMUSG00000073738 | MMT00020512 |  |
| MMT00059699 | ENSMUSG00000074408 | MMT00023482 |  |
| BE952823 | ENSMUSG00000074461 | MMT00026128 |  |
| MMT00055617 | ENSMUSG00000075545 | MMT00026698 |  |
| AK033787 | Entpd7 | MMT00036295 |  |
| AK010038 | Epb4.1 | MMT00039922 |  |
| AK047254 | Epb4.1 | MMT00041810 |  |
| MMT00074488 | Epb4.1l2 | MMT00042505 |  |
| NM_013813 | Epb4.1l3 | MMT00058026 |  |
| MMT00036214 | Epb4.1l5 | MMT00065890 |  |
| NM_013514 | Epb4.9 | MMT00079618 |  |
| NM_010139 | Epha2 | MMT00082897 |  |
| AI326877 | Ephb1 | NM_177792 |  |
| NM_010144 | Ephb4 | ri|D530018E20|PX00089G07||1058 |  |
| U89491 | Ephx1 | XM_124621 |  |
| AF057286 | Epn2 | XM_128982 |  |
| NM_007943 | Eps15 | MMT00038383 |  |
| MMT00066214 | Erc1 | MMT00004439 |  |
| MMT00061554 | Erg | MMT00018063 |  |
| NM_026170 | Ergic1 | MMT00012327 |  |
| NM_007951 | Erh | mCT155803 |  |
| U01140 | Erh | MMT00009560 |  |
| MMT00025651 | Erlin1 | MMT00003880 |  |
| MMT00040982 | Ermp1 | mCT51711 |  |
| NM_026184 | Ero1lb | mCT49287 |  |
| AK004851 | Errfi1 | MMS010002259 |  |
| MMT00032840 | Esam1 | AK014334 |  |
| NM_011934 | Esrrb | BE634458 |  |
| AK014602 | Ets2 | XM_359042 |  |
| NM_007960 | Etv1 | MMT00077828 |  |
| NM_012051 | Etv3 | MMS010183492 |  |
| AK018368 | Etv5 | MMS010172569 |  |
| AK003753 | Evc2 | NM_012022 |  |
| MMT00049184 | Evi1 | MMT00038313 |  |
| MMT00076501 | Exdl1 | MMT00016184 |  |
| MMT00063827 | Exoc6 | XM_286414 |  |
| NM_198103 | Exoc8 | AK083072 |  |
| NM_018788 | Extl3 | AK084064 |  |
| MMT00008589 | Eya1 | MMS010230978 |  |
| NM_007970 | Ezh1 | mCT186072 |  |
| NM_028784 | F13a1 | AK045876 |  |
| MMT00032175 | Fa2h | MMT00055874 |  |
| MMT00051511 | Fabp4 | MMT00039914 |  |
| M20497 | Fabp4 | XM_109346 |  |
| NM_011598 | Fabp9 | AK083345 |  |
| NM_021890 | Fads3 | MMT00077107 |  |
| NM_007983 | Faf1 | AK011092 |  |
| NM_010176 | Fah | AK018238 |  |
| X13135 | Fasn | AK018410 |  |
| AK009264 | Fastkd3 | AK019217 |  |
| MMT00058244 | Fat4 | AK029936 |  |
| AK002355 | Fau | AK030675 |  |
| NM_176959 | Fbxl7 | AK036907 |  |
| AK051849 | Fbxl7 | AK049667 |  |
| MMT00036789 | Fbxo18 | AK051929 |  |
| AK013157 | Fbxo21 | AK052943 |  |
| AK002745 | Fbxo22 | AK081316 |  |
| AK004544 | Fbxo3 | AK085773 |  |
| MMT00059226 | Fbxo31 | AK087708 |  |
| AI480454 | Fbxo4 | AV235941 |  |
| AJ300659 | Fbxo4 | AV270875 |  |
| AK011438 | Fbxo45 | AV378554 |  |
| MMT00074844 | Fbxo45 | BB076186 |  |
| NM_023605 | Fbxo9 | BB209038 |  |
| ENSMUST00000066901 | Fbxw14 | BB556507 |  |
| MMT00038411 | Fcho2 | BC028660 |  |
| NM_030707 | Fcrls | BE988431 |  |
| MMT00058866 | Fermt2 | BE995312 |  |
| AK009310 | Fetub | ENSMUST00000030126 |  |
| MMT00025127 | Fgfr1 | ENSMUST00000050624 |  |
| NM_054071 | Fgfrl1 | mCT146825 |  |
| MMT00022190 | Fggy | mCT14765 |  |
| MMT00073564 | Fh1 | mCT148556 |  |
| BC006048 | Fh1 | mCT163746 |  |
| NM_177699 | Fhod1 | mCT184017 |  |
| AK003331 | Fkbp11 | mCT185037 |  |
| MMT00009325 | Flad1 | mCT50110 |  |
| NM_008027 | Flot1 | mCT50719 |  |
| NM_008028 | Flot2 | mCT7664 |  |
| NM_010228 | Flt1 | MMS010031258 |  |
| MMT00043896 | Flt3 | MMS010083472 |  |
| MMT00058798 | Flywch1 | MMS010204057 |  |
| MMT00044504 | Flywch2 | MMS010208198 |  |
| NM_010231 | Fmo1 | MMS010251316 |  |
| NM_018881 | Fmo2 | MMT00000172 |  |
| NM_008030 | Fmo3 | MMT00000983 |  |
| AK017179 | Fmo5 | MMT00004171 |  |
| NM_008031 | Fmr1 | MMT00004656 |  |
| MMT00020926 | Fndc3a | MMT00005496 |  |
| AK005500 | Fndc3b | MMT00005901 |  |
| AK017346 | Foxn3 | MMT00006746 |  |
| AJ252157 | Foxo1 | MMT00007070 |  |
| MMT00043850 | Foxo4 | MMT00007996 |  |
| NM_054039 | Foxp3 | MMT00010950 |  |
| MMT00036098 | Foxred1 | MMT00012680 |  |
| MMT00040719 | Fras1 | MMT00013996 |  |
| NM_010237 | Frk | MMT00014239 |  |
| MMT00076484 | Fry | MMT00017591 |  |
| NM_178781 | Fry | MMT00022500 |  |
| NM_177136 | Fryl | MMT00023282 |  |
| AK009138 | Fryl | MMT00025735 |  |
| XM_357268 | Frzb | MMT00027411 |  |
| MMT00008735 | Fsd2 | MMT00030667 |  |
| BC003235 | Fuca1 | MMT00033071 |  |
| AK080516 | Fus | MMT00033309 |  |
| BE136454 | Fut4 | MMT00033384 |  |
| NM_019503 | Fxyd1 | MMT00033789 |  |
| AK003888 | Fxyd6 | MMT00033923 |  |
| NM_008055 | Fzd4 | MMT00034261 |  |
| MMT00030488 | Gabra3 | MMT00034521 |  |
| MMT00059802 | Gabrr2 | MMT00034780 |  |
| mCT17487 | Gal3st3 | MMT00036668 |  |
| BF147815 | Gal3st4 | MMT00039380 |  |
| MMT00025317 | Galm | MMT00041145 |  |
| MMT00069484 | Galnt12 | MMT00041380 |  |
| MMT00030035 | Galnt7 | MMT00043042 |  |
| ENSMUST00000021558 | Galntl1 | MMT00044519 |  |
| MMT00067927 | Galntl2 | MMT00044927 |  |
| MMT00057432 | Ganc | MMT00046015 |  |
| AK017994 | Ganc | MMT00046046 |  |
| AK012758 | Gars | MMT00046147 |  |
| AK009556 | Gatad1 | MMT00048362 |  |
| MMT00047493 | Gatad2b | MMT00048675 |  |
| AK013689 | Gcap14 | MMT00049235 |  |
| AK017462 | Gcnt1 | MMT00052665 |  |
| NM_020619 | Gcs1 | MMT00054214 |  |
| Y17854 | Gdap5 | MMT00054302 |  |
| MMT00062374 | Gdf1 | MMT00056077 |  |
| NM_010276 | Gem | MMT00058347 |  |
| NM_177331 | Gen1 | MMT00060103 |  |
| MMT00075569 | Gfod1 | MMT00061351 |  |
| AK017684 | Gfod2 | MMT00064683 |  |
| MMT00070051 | Gfra2 | MMT00069008 |  |
| ri|1010001P14|R000005B18||2470 | Ghitm | MMT00070435 |  |
| NM_010284 | Ghr | MMT00078406 |  |
| ri|4833419H03|PX00028G07||1566 | Gimap6 | V00716 |  |
| AK016667 | Gins4 | XM_122526 |  |
| MMT00047355 | Gja4 | XM_124427 |  |
| NM_008122 | Gjc1 | XM_124826 |  |
| NM_008132 | Glrp1 | XM_129965 |  |
| ENSMUST00000020485 | Glt8d2 | XM_135428 |  |
| MMT00047083 | Glyctk | XM_148086 |  |
| mCT8791 | Gm1019 | XM_193155 |  |
| MMT00024998 | Gm1967 | XM_193289 |  |
| MMT00066705 | Gm266 | XM_193900 |  |
| U09816 | Gm2a | XM_354567 |  |
| MMT00081669 | Gm525 | XM_354736 |  |
| XM_358997 | Gm608 | XM_354819 |  |
| XM_205232 | Gm715 | XM_355609 |  |
| MMT00026895 | Gm98 | XM_355808 |  |
| MMT00081360 | Gmeb1 | XM_355975 |  |
| MMT00025256 | Gmpr | XM_356455 |  |
| MMT00080984 | Gnai1 | XM_356574 |  |
| BE990491 | Gnal | XM_357054 |  |
| AK011372 | Gnal | XM_357610 |  |
| MMS010076068 | Gnao1 | XM_358291 |  |
| BC044739 | Gnb2l1 | AK011060 |  |
| MMT00078482 | Gnb2l1 | AK016900 |  |
| MMT00050167 | Gng3 | AK032250 |  |
| MMT00069352 | Gng7 | AK035284 |  |
| AK004771 | Gnpat | AK036153 |  |
| MMT00057928 | Gnptab | AK038233 |  |
| NM_008149 | Gpam | AK044374 |  |
| MMT00043939 | Gpihbp1 | AK077892 |  |
| AK003422 | Gpm6a | AK082418 |  |
| AF254875 | Gpm6b | AK089448 |  |
| NM_133756 | Gpn1 | AV312707 |  |
| MMT00064215 | Gpr116 | AV359277 |  |
| MMT00012897 | Gpr116 | BE995259 |  |
| MMT00080930 | Gpr120 | BZ894129 |  |
| MMT00041083 | Gpr124 | ENSMUST00000069788 |  |
| NM_030258 | Gpr146 | mCT120254 |  |
| MMT00063768 | Gpr27 | mCT131108 |  |
| ENSMUST00000066741 | Gpr62 | mCT146380 |  |
| MMT00033867 | Gpr81 | mCT147199 |  |
| MMT00063004 | Gprasp2 | mCT151492 |  |
| AB030197 | Gprc5b | mCT153549 |  |
| AI647917 | Gprc5c | mCT154416 |  |
| BC005757 | Gprc5c | mCT157409 |  |
| AK004932 | Gpt | mCT184486 |  |
| MMT00074983 | Gpx4 | mCT2278 |  |
| MMT00026543 | Grap | mCT49441 |  |
| AK017862 | Grb10 | mCT51034 |  |
| NM_023168 | Grina | mCT52201 |  |
| MMT00023629 | Grip1 | mCT65921 |  |
| NM_010356 | Gsta3 | MMS010005499 |  |
| J04632 | Gstm1 | MMS010170903 |  |
| AK015265 | Gstm6 | MMS010225599 |  |
| NM_010363 | Gstz1 | MMS010235951 |  |
| MMT00023488 | Gtf2h5 | MMS010260530 |  |
| MMT00050691 | Gtl3 | MMT00000323 |  |
| MMT00056372 | Gucy1a3 | MMT00000406 |  |
| NM_008194 | Gyk | MMT00003599 |  |
| NM_030678 | Gys1 | MMT00005726 |  |
| NM_008195 | Gys1 | MMT00006959 |  |
| MMT00049794 | Gzf1 | MMT00007908 |  |
| AK018355 | H2afy2 | MMT00009590 |  |
| BC051559 | H2-K1 | MMT00010545 |  |
| MMT00078032 | H6pd | MMT00011633 |  |
| NM_019975 | Hacl1 | MMT00012324 |  |
| AK008993 | Hamp2 | MMT00013076 |  |
| NM_008217 | Has3 | MMT00013473 |  |
| MMT00034621 | Hat1 | MMT00015001 |  |
| NM_008218 | Hba-a1 | MMT00018835 |  |
| MMT00030290 | Hba-a1 | MMT00022456 |  |
| V00722 | Hbb-b1 | MMT00023029 |  |
| AK011062 | Hbb-b2 | MMT00023936 |  |
| NM_010406 | Hc | MMT00024189 |  |
| NM_008230 | Hdc | MMT00024952 |  |
| MMT00056074 | Hdhd2 | MMT00025998 |  |
| ENSMUST00000001002 | Heatr6 | MMT00029182 |  |
| MMT00073816 | Hebp2 | MMT00033125 |  |
| AK129325 | Hecw2 | MMT00033380 |  |
| AK017435 | Heg1 | MMT00033801 |  |
| AK020573 | Heg1 | MMT00035514 |  |
| NM_172934 | Heg1 | MMT00037028 |  |
| MMT00015713 | Helz | MMT00037210 |  |
| AK020667 | Helz | MMT00037360 |  |
| MMT00078485 | Hemk1 | MMT00037611 |  |
| NM_026101 | Herc4 | MMT00037681 |  |
| NM_010422 | Hexb | MMT00038895 |  |
| NM_010423 | Hey1 | MMT00040053 |  |
| NM_013905 | Heyl | MMT00040108 |  |
| Y12650 | Hfe | MMT00040267 |  |
| ri|2610509I15|ZX00045F23||1702 | Hibch | MMT00041188 |  |
| AF071070 | Hipk1 | MMT00041497 |  |
| AK003718 | Hipk2 | MMT00043422 |  |
| MMT00069823 | Hipk2 | MMT00043723 |  |
| NM_010433 | Hipk2 | MMT00044598 |  |
| MMT00072884 | Hipk3 | MMT00046201 |  |
| X92590 | Hira | MMT00046711 |  |
| NM_178204 | Hist1h3d | MMT00047239 |  |
| NM_178205 | Hist1h3e | MMT00047735 |  |
| NM_178193 | Hist1h4b | MMT00048286 |  |
| AV054097 | Hist1h4h | MMT00048556 |  |
| MMT00011655 | Hist1h4j | MMT00049680 |  |
| MMT00021525 | Hist1h4k | MMT00050471 |  |
| NM_175657 | Hist1h4m | MMT00050488 |  |
| NM_175666 | Hist2h2bb | MMT00051556 |  |
| NM_178215 | Hist2h3b | MMT00051849 |  |
| NM_175652 | Hist4h4 | MMT00052654 |  |
| MMT00059826 | Hlcs | MMT00052916 |  |
| MMT00066449 | Hmgcr | MMT00053154 |  |
| NM_008251 | Hmgn1 | MMT00053509 |  |
| BC005693 | Hmgn3 | MMT00054707 |  |
| MMT00055837 | Hnmt | MMT00055071 |  |
| AK010481 | Hnrnpa3 | MMT00056548 |  |
| MMT00056235 | Hnrnpab | MMT00059805 |  |
| mCT12041 | Hnrnph3 | MMT00060809 |  |
| MMT00070661 | Hnrnpul2 | MMT00061530 |  |
| AB017136 | Homer2 | MMT00062059 |  |
| BC002226 | Hook2 | MMT00065353 |  |
| NM_010449 | Hoxa1 | MMT00069486 |  |
| NM_175730 | Hoxc5 | MMT00071048 |  |
| MMT00054796 | Hoxc6 | MMT00073093 |  |
| X07439 | Hoxc8 | MMT00075917 |  |
| MMT00049264 | Hps5 | MMT00076151 |  |
| MMT00072197 | Hr | MMT00077363 |  |
| MMT00050877 | Hrasls3 | MMT00079369 |  |
| MMT00064495 | Hscb | MMT00080308 |  |
| NM_008288 | Hsd11b1 | MMT00080880 |  |
| AK012103 | Hsd17b12 | MMT00081007 |  |
| X89998 | Hsd17b4 | MMT00081342 |  |
| X89998 | Hsd17b4 | MMT00081719 |  |
| MMT00072818 | Hspa12a | NM_019643 |  |
| NM_028306 | Hspa12b | NM_020568 |  |
| NM_010481 | Hspa9 | ri|2310043N10|ZX00040G07||2099 |  |
| BB232455 | Hspb6 | XM_122214 |  |
| MMT00070846 | Hspb6 | XM_123814 |  |
| NM_030704 | Hspb8 | XM_124376 |  |
| NM_178637 | Htatip | XM_125109 |  |
| NM_010483 | Htr5b | XM_126058 |  |
| AK008764 | Htra3 | XM_130020 |  |
| MMT00047727 | Htra4 | XM_130714 |  |
| ENSMUST00000046642 | Huwe1 | XM_142323 |  |
| MMT00054048 | Hyal1 | XM_193332 |  |
| AK012313 | Hyi | XM_283518 |  |
| AK020099 | Hyi | XM_287286 |  |
| M31885 | Id1 | XM_289922 |  |
| NM_008321 | Id3 | XM_355862 |  |
| NM_025409 | Ier3ip1 | XM_356820 |  |
| MMT00055270 | Ifitm6 | AI552429 |  |
| MMT00077139 | Ifngr2 | AI852066 |  |
| NM_010514 | Igf2 | AK034726 |  |
| ri|0610008B05|R000001B10||2069 | Igfbp4 | AK040326 |  |
| L12447 | Igfbp5 | AK086771 |  |
| MMS010257092 | Igh-2 | AV094422 |  |
| ENSMUST00000004000 | Igh-6 | BB283961 |  |
| mCT156879 | Igh-V3609N | BC006049 |  |
| MMT00027505 | Igh-VJ558 | BC019586 |  |
| U38854 | Igh-VJ558 | BE950450 |  |
| AK019524 | Igsf3 | BE995972 |  |
| MMT00059199 | Il13ra1 | ENSMUST00000057415 |  |
| NM_008356 | Il13ra2 | mCT118834 |  |
| MMT00009197 | Il15ra | mCT155118 |  |
| BC004759 | Il17rc | mCT184674 |  |
| MMT00006020 | Il17rd | mCT49384 |  |
| AK012666 | Il17rd | mCT49516 |  |
| MMT00006432 | Il17re | MMS010090934 |  |
| MMT00053778 | Il20rb | MMT00000194 |  |
| MMT00037070 | Il23r | MMT00000549 |  |
| AK052920 | Immt | MMT00002494 |  |
| ri|2610002K09|ZX00060D02||2140 | Impa1 | MMT00002637 |  |
| AK004978 | Ing3 | MMT00003753 |  |
| NM_008380 | Inhba | MMT00004295 |  |
| AK002281 | Inmt | MMT00005788 |  |
| MMT00072923 | Inpp1 | MMT00007609 |  |
| BC023968 | Inpp5a | MMT00007741 |  |
| NM_010566 | Inpp5d | MMT00009510 |  |
| AK007471 | Insig1 | MMT00011302 |  |
| AK013679 | Insig2 | MMT00012377 |  |
| MMT00008900 | Insr | MMT00014243 |  |
| AK015941 | Ints12 | MMT00014425 |  |
| AK020350 | Intu | MMT00015399 |  |
| NM_182784 | Iqsec1 | MMT00017829 |  |
| NM_008391 | Irf2 | MMT00017875 |  |
| NM_013674 | Irf4 | MMT00017964 |  |
| MMT00070165 | Irs1 | MMT00019644 |  |
| MMT00006763 | Irs2 | MMT00020721 |  |
| MMT00059600 | Irs3 | MMT00021596 |  |
| AK009421 | Iscu | MMT00021660 |  |
| NM_177193 | Islr2 | MMT00023238 |  |
| MMT00051620 | Itga1 | MMT00028928 |  |
| BC014765 | Itga1 | MMT00030495 |  |
| NM_010575 | Itga2b | MMT00031107 |  |
| MMT00055752 | Itgad | MMT00032659 |  |
| MMT00054996 | Itgam | MMT00036444 |  |
| AK014514 | Itih5 | MMT00038309 |  |
| NM_008409 | Itm2a | MMT00038343 |  |
| NM_022417 | Itm2c | MMT00039978 |  |
| NM_010585 | Itpr1 | MMT00040260 |  |
| MMT00007259 | Ivd | MMT00040937 |  |
| AW490567 | Jag1 | MMT00042142 |  |
| MMT00006097 | Jam2 | MMT00042303 |  |
| MMT00061892 | Jam3 | MMT00046256 |  |
| BC010717 | Jarid1a | MMT00048617 |  |
| MMT00082107 | Jazf1 | MMT00048939 |  |
| MMT00003975 | Jhdm1d | MMT00050022 |  |
| MMT00050081 | Jmjd3 | MMT00050724 |  |
| MMT00004756 | Jup | MMT00051519 |  |
| NM_007969 | Kal1 | MMT00052895 |  |
| MMT00076251 | Kank2 | MMT00056634 |  |
| MMT00061373 | Kank3 | MMT00058919 |  |
| NM_010594 | Kap | MMT00061825 |  |
| NM_008419 | Kcna5 | MMT00068363 |  |
| NM_008420 | Kcnb1 | MMT00070775 |  |
| AK004144 | Kcnd1 | MMT00071128 |  |
| NM_010601 | Kcnh3 | MMT00076184 |  |
| MMT00006503 | Kcnq4 | MMT00078098 |  |
| MMT00047339 | Kcns3 | MMT00080853 |  |
| MMT00012352 | Kctd10 | MMT00081103 |  |
| MMT00074808 | Kctd15 | MMT00081195 |  |
| MMT00051702 | Kctd17 | MMT00082224 |  |
| NM_010612 | Kdr | NM_023627 |  |
| MMT00013487 | Kera | NM_176969 |  |
| NM_010158 | Khdrbs3 | X16496 |  |
| MMT00008815 | Kif13a | XM_110983 |  |
| MMT00057801 | Kif1b | XM_125110 |  |
| AK082050 | Kif1b | XM_140734 |  |
| MMT00049280 | Kif3c | XM_146216 |  |
| AK013621 | Kirrel3 | XM_193846 |  |
| NM_178260 | Kiss1 | XM_194250 |  |
| BC016531 | Kiss1r | XM_195378 |  |
| NM_021099 | Kit | XM_284099 |  |
| NM_013598 | Kitl | XM_284704 |  |
| NM_031180 | Klb | XM_354622 |  |
| MMT00044644 | Klf12 | XM_355149 |  |
| MMT00043167 | Klf14 | XM_355207 |  |
| AK007959 | Klf3 | XM_356224 |  |
| AF338369 | Klf7 | XM_356760 |  |
| MMT00048096 | Klhdc7a | XM_356875 |  |
| NM_026167 | Klhl13 | XM_357496 |  |
| AK020050 | Klhl2 | XM_359043 |  |
| BB347906 | Klhl24 | AI504897 |  |
| MMT00030083 | Klhl4 | AK052334 |  |
| MMT00015438 | Klhl5 | AK076309 |  |
| ENSMUST00000031089 | Klhl5 | AK084150 |  |
| MMT00007626 | Klhl7 | BF682209 |  |
| NM_174865 | Klk15 | ENSMUST00000036218 |  |
| NM_008459 | Klra10 | ENSMUST00000041714 |  |
| MMT00034127 | Klra19 | ENSMUST00000064071 |  |
| NM_010648 | Klra3 | mCT10101 |  |
| NM_010649 | Klra4 | mCT154190 |  |
| NM_010737 | Klrb1a | mCT160133 |  |
| NM_008526 | Klrb1b | mCT180896 |  |
| MMT00015928 | Klrb1b | mCT48903 |  |
| mCT133896 | Klrb1-ps1 | mCT49853 |  |
| NM_010653 | Klrc2 | mCT54780 |  |
| MMT00031284 | Kmo | MMS010208241 |  |
| AK009746 | Kptn | MMT00001194 |  |
| BC005704 | Krcc1 | MMT00009535 |  |
| AK014360 | Krt10 | MMT00011144 |  |
| NM_010660 | Krt10 | MMT00011590 |  |
| MMT00018540 | Krt20 | MMT00015231 |  |
| NM_028770 | Krt80 | MMT00015428 |  |
| M92088 | Krt86 | MMT00020268 |  |
| MMT00019380 | Krtap5-4 | MMT00021170 |  |
| MMT00031531 | Lace1 | MMT00025975 |  |
| BC004045 | Lactb2 | MMT00030143 |  |
| MMT00071495 | Lag3 | MMT00032904 |  |
| NM_008485 | Lamc2 | MMT00034010 |  |
| MMT00010915 | Lamp2 | MMT00035426 |  |
| ri|4733401J09|PX00013I01||2080 | Lass4 | MMT00035807 |  |
| MMT00073479 | Lass6 | MMT00038910 |  |
| NM_133815 | Lbr | MMT00039962 |  |
| MMT00068415 | Lcn2 | MMT00041363 |  |
| MMT00051426 | Ldb2 | MMT00042063 |  |
| MMT00059054 | Lgals1 | MMT00049245 |  |
| NM_019516 | Lgals12 | MMT00049534 |  |
| MMT00047650 | Lhfpl4 | MMT00052332 |  |
| NM_024471 | Lias | MMT00054521 |  |
| NM_010715 | Lig1 | MMT00057778 |  |
| MMT00075754 | Limch1 | MMT00059421 |  |
| MMT00081290 | Lims2 | MMT00060943 |  |
| NM_008280 | Lipc | MMT00064206 |  |
| NM_010719 | Lipe | MMT00065667 |  |
| MMT00045185 | Lipt1 | MMT00065763 |  |
| MMT00034396 | Lix1 | MMT00071470 |  |
| MMT00054330 | Lman1 | MMT00072683 |  |
| AV143193 | Lmo1 | MMT00073650 |  |
| MMT00025454 | Lmo1 | MMT00075644 |  |
| MMT00068851 | Lmtk2 | MMT00079701 |  |
| NM_027133 | Lnp | MMT00082206 |  |
| AK008544 | Lnpep | MMT00082297 |  |
| NM_025827 | Lonp2 | MMT00082629 |  |
| BC058671 | Lonrf3 | ri|0610039K23|R000004P19||1590 |  |
| MMT00068519 | Lpcat2 | X82357 |  |
| MMT00030178 | Lpgat1 | XM_122017 |  |
| AK013763 | Lphn1 | XM_122093 |  |
| NM_015763 | Lpin1 | XM_122578 |  |
| BC005613 | Lpp | XM_128314 |  |
| AK082089 | Lrch1 | XM_136685 |  |
| MMT00039475 | Lrfn4 | XM_138781 |  |
| MMT00049532 | Lrig2 | XM_140612 |  |
| MMT00074754 | Lrp11 | XM_142485 |  |
| MMT00022757 | Lrrc20 | XM_143363 |  |
| NM_145152 | Lrrc3 | XM_144987 |  |
| ri|9430028I06|PX00108K24||1318 | Lrrc39 | XM_193145 |  |
| BB559889 | Lrrc4c | XM_193290 |  |
| AK007464 | Lrrc58 | XM_205095 |  |
| ri|2210408K08|ZX00054O17||1834 | Lrrc8b | XM_283482 |  |
| MMT00007792 | Lrrn2 | XM_283789 |  |
| MMT00031965 | Lsm10 | XM_354772 |  |
| NM_177727 | Lsm14b | XM_355183 |  |
| MMT00068215 | Lsm7 | XM_355509 |  |
| XM_359348 | Lsm7 | XM_356689 |  |
| MMT00001318 | Lsm8 | XM_356920 |  |
| AK014149 | Lt1 | XM_357307 |  |
| AK009837 | Ltbp4 | AV140144 |  |
| AK084533 | Luc7l2 | BC032044 |  |
| MMT00072309 | Ly6g6c | BE950594 |  |
| NM_033478 | Ly6g6d | BE989079 |  |
| AK002674 | Lypla1 | ENSMUST00000065221 |  |
| AK004726 | Lyve1 | mCT163646 |  |
| NM_178369 | Madd | MMT00000595 |  |
| NM_010757 | Mafk | MMT00001962 |  |
| MMT00041376 | Mageb18 | MMT00002955 |  |
| NM_030700 | Maged2 | MMT00011814 |  |
| MMT00012179 | Magi1 | MMT00014048 |  |
| AK020134 | Malat1 | MMT00016208 |  |
| MMT00007690 | Mall | MMT00016884 |  |
| NM_173776 | Maml2 | MMT00021463 |  |
| BB383081 | Maml3 | MMT00022831 |  |
| AK078047 | Man1a2 | MMT00039793 |  |
| MMT00042759 | Man2a2 | MMT00047828 |  |
| MMT00026894 | Manba | MMT00050545 |  |
| AK053830 | Map3k10 | MMT00054567 |  |
| AK004850 | Map3k13 | MMT00058413 |  |
| NM_008580 | Map3k5 | MMT00058564 |  |
| NM_008696 | Map4k4 | MMT00061589 |  |
| NM_027115 | Mapk1ip1 | MMT00062934 |  |
| AK011505 | Mapk6 | MMT00067071 |  |
| AI593086 | Mapk6 | MMT00070942 |  |
| MMT00054835 | Mapkap1 | MMT00072213 |  |
| MMT00075542 | Mapkapk5 | MMT00074240 |  |
| NM_010807 | Marcksl1 | NM_011222 |  |
| NM_019945 | Mast1 | XM_124310 |  |
| NM_008641 | Mast2 | XM_125403 |  |
| MMT00054724 | Mast4 | XM_193969 |  |
| NM_010770 | Matn3 | XM_194428 |  |
| NM_008558 | Max | XM_194613 |  |
| AF098634 | Mbc2 | XM_203984 |  |
| D11441 | Mbl1 | XM_355809 |  |
| MMT00066220 | Mbnl2 | XM_358474 |  |
