# Supplementary material for: Liver and Adipose Expression Associated SNPs Are Enriched for Association to Type 2 Diabetes
Source: PLoS Genet. 2010 May 6;6(5):e1000932. doi: 10.1371/journal.pgen.1000932 (PMC2865508; doi:10.1371/journal.pgen.1000932)
Supplement: Text S1 — Supplementary methods and discussion. (0.18 MB DOC) [file pgen.1000932.s006.doc]

**Text S1**

**eSNP Processing for the Human Liver Cohort**

The first human cohort providing liver tissue samples is comprised of 427 Caucasian subjects and was previously described [1]. DNA and RNA were isolated from all liver tissue samples collected form all subjects. Each RNA sample was profiled on a custom Agilent array with 39,280 oligonucleotide probes targeting transcripts representing 34,266 known and predicted genes, including high-confidence noncoding RNA sequences. Each DNA sample was genotyped on the Affymetrix 500K SNP and Illumina 650Y SNP genotyping arrays. Analysis was restricted to those SNPs that had a genotyping call rate greater than 75%, a minor allele frequency greater than 4%, and that did not deviate significantly from Hardy-Weinberg equilibrium in this cohort, as previously described [1]. A total of 310,744 and 557,240 SNPs met these criteria from the Affymetrix and Illumina sets, respectively, resulting in a set of 782,476 unique SNPs (85,508 SNPs were in the intersection). All expression data have been deposited into the Gene Expression Omnibus database under accession number (GSE9588).

**eSNP Analysis for the Multi Tissue Cohort**

The multi tissue cohort was comprised of patients who underwent RXY gastric bypass surgery. Liver, subcutaneous adipose and omental adipose tissues were collected from each patient at the time of surgery at Massachusetts General Hospital. Genomic DNA was extracted from liver tissues, and total RNA was extracted from liver, subcutaneous adipose and omental adipose tissues. Each RNA sample was profiled on the custom 44K Agilent array described above. Each DNA sample was genotyped on the Illumina 650Y BeadChip array. 950 samples were successfully genotyped.

Total RNA was isolated from each tissue and converted to fluorescently labeled cRNA that was hybridized to DNA oligonucleotide microarrays as described previously [2]. Briefly, 4 µg of total RNA from each treated sample was used to synthesize dsDNA through reverse transcription. cRNA was produced by *in vitro* transcription and labeled post-synthetically with Cy3 or Cy5. cRNA derived from growth factor or AKT inhibitor treated cells (experimental sample) was hybridized against cRNA derived from an equal mixture of all the sample samples (reference sample). Two hybridizations were done with each cRNA sample pair probes on the microarrays were synthesized *in situ* with inkjet technology (Agilent Technologies, Palo Alto, CA; [2]). After hybridization, arrays were scanned and fluorescence intensities for each probe were recorded. Ratios of transcript abundance (experimental to control) were obtained following normalization and correction of the array intensity data. Gene expression data was analyzed using Rosetta Resolver gene expression analysis software (version 7.0, Rosetta Biosoftware, Seattle, WA) and MATLAB (The MathWorks, Natick, MA).

IBS analysis was performed to identify related individuals. Eighteen parent-offspring, 6 sibling and 8 second degree relatives were identified, 4 of these were related trios. 28 individuals were removed to eliminate IBS in the dataset, leaving 922 samples for use in the analysis. Successful gene expression profiling results were collected from 707 liver samples, 916 omental adipose samples and 870 subcutaneous adipose samples.

Demographic information including age, race, gender, height, type of surgery and year of surgery were collected for each patient. All surgeries occurred between 2000 and 2007, with 59% of the surgeries performed laproscopically and the rest performed open. Weight and blood levels of leptin were collected for each individual at varying times from 1 year pre-op to 7 years post-op. The post-op data available on individuals varies depending on when the surgery was performed. BMI was calculated as WT(kg)/HT(M)^2. Excess weight loss (EWL) was calculated as (BMI initial-BMI final)/(BMI initial-BMI ideal). Previous analyses have shown the effect demographic information may have on gene expression and genetic data [3]. The independent contribution of each of the covariates age, race, gender, surgery year, surgery type and BMI were analyzed. The distribution of p-values obtained from an ANOVA model for each covariate strongly suggests that correcting for age, race, gender and surgery year is necessary. By removing the contribution from surgery year, the surgery type became unimportant suggesting a strong correlation between these two covariates***.*** During the course of sample collection the surgery type was switched from being performed as an open surgery to a laparoscopic surgery. This change likely explains the correlation between these two traits. BMI was shown to have a normal distribution. As a result, all gene expression traits were adjusted for these covariates. Additional covariates used in the analysis included the EIGENSTRAT first principle component, diabetes status, insulin use, metformin use and statin use. The gene expression adjustment residuals were used to detect for association with SNPs.

All expression traits (residuals) were tested for association with each of the genotyped SNPs meeting the filtering criteria. *Cis* and *trans* acting expression quantitative trait loci (eQTLs) were identified using a method similar to that previously described [4]. The *cis* eQTL for a given expression trait was defined as the SNP located within 1 megabase (Mb) of the transcription start or stop of the corresponding structural gene. All other associations were considered *trans*. SNP associations were identified using the Kruskal-Wallis test. The association *p*-values were adjusted to control for testing of multiple SNPs and expression traits using an empirically determined false discovery rate (FDR) constrained to be < 10%. For *cis* eQTL, we only test for associations to SNPs that are within 1 Mb of the annotated start or stop site of the corresponding structural gene. The permutations were restricted to SNPs within the *cis* regions. In the case of *trans* eQTL, all SNPs were tested for association to each of the expression traits. Where SNP associations were identified to the same trait in high LD with each other, the SNP with the most significant p-value was reported.

**Normal Approximation to Derive the Permutation Pvalues:** For each random SNP set, we counted the percentage of SNPs with GWAS p < 0.05, $P_{0.05}^{1}\ldots P_{0.05}^{100,000}$, and constructed the null distribution based on these counts. We then compared the observed percentage of eSNPs with GWAS p < 0.05 to the null distribution and obtained the enrichment P value of the eSNP set. An approximation method, which is used to increase the enrichment P value resolution, was also used. By the central limit theorem, the null distribution should approximately follow a normal distribution. This was confirmed by direct observation. We then compared the observed percentage of eSNPs with GWAS p < 0.05 in the eSNP set, $P_{0.05}^{obs}$, to the null distribution. We defined the Z statistic as, and the p value for enrichment at the 0.05 level was thus calculated as $\Phi(Z)$, where $\Phi(\cdot)$ is the standard normal cumulative probability. Here we used the p value derived from the normal distribution rather than defining it as the percentage exceeding the observed P in the eSNP set from random samples ($P=\frac{\sum_{1}^{B} 1(P_{0.05}^{i}>=P_{0.05}^{obs})}{B}$). We chose to report the theoretical p values to increase the resolution of our results, given over most of the testing there were very few p values from the 100,000 random samples that were smaller than those for the observed eSNP sets. In cases where empirical p values were greater than zero, they were consistent with the theoretical p values.

**Generation of B6×BTBR cross F_2_ Mice**

554 F_2_ mice were generated in a cross of two inbred strains, both containing the *ob* mutation at the leptin locus: C57BL/6 *ob/ob* and BTBR *ob/ob* (referred to as the B6×BTBR cross) [5]. All F_2_ animals were maintained on a chow diet for ten weeks and were clinically characterized with respect to obesity- and diabetes-related traits at the timepoints of four, six, eight and ten weeks. Further details regarding the plasma glucose and insulin measurements, as well as islet isolation procedures, can be found in Keller et al. [6]. At the time of necropsy, gonadal white adipose tissue was collected from 497 mice. RNA was prepared using the same methods as described previously [6] and hybridized to Agilent custom murine gene expression microarrays for profiling.

**Reconstruction of the Murine Adipose Network**

Gonadal white adipose tissues were extracted from F_2_ animals in a B6xBTBR cross and profiled on an Agilent custom murine gene expression microarray. Of the 39,600 transcribed sequences represented on the microarray, the top 25 percent of genes rank ordered by degree of differential expression in the adipose tissue were included in the reconstruction. A previously described weighted gene coexpression network reconstruction algorithm was employed to reconstruct the adipose and islet coexpression networks [7]. The weighted network reconstruction algorithm involved first constructing a matrix of Pearson correlations between all gene expression pairs. The correlation matrix was then transformed into an adjacency matrix using the power function $f\left(x\right)=x^{\beta}$. The adjacency matrix defines the weighted coexpression network. The parameter *β* of the power function was determined such that the resulting adjacency matrix was approximately scale-free. To measure how well a network satisfies the scale-free topology property, we used a model fitting index [7]. This index is defined as the coefficient of determination (i.e., *R^2^* ) of the linear model constructed by regressing $\log\left(p\left(k\right)\right)$ onto $\log\left(k\right)$, where *k* represents the degree of a given node (i.e., the number of edges connecting to the given node), and *p(k)* is the frequency distribution of the degree *k* in the coexpression network. The model fitting index of a perfect scale-free network is 1. The exponent of the power function, $\beta$, was chosen to be the smallest value such that the coexpression network exhibited the scale free property . The degree distribution of the coexpression network approximates a power law with a model fitting index > 0.8. In this way, $\beta$ was set to 4 for the adipose network. To identify modules of highly co-regulated genes, we used average linkage hierarchical clustering to group genes based on the topological overlap of their connectivity, followed by a dynamic cut-tree algorithm to dynamically cut clustering dendrogram branches into gene modules [8]. We identified 19 modules in the adipose network.

**Construction and phenotypic characterization of *Me1*-/- mice**

A naturally occurring mouse mutant deficient in Me1 enzymatic activity was first reported by Lee *et al.* in 1980 [9]. The detailed methods for breeding, genotyping, and characterization of the *Me1^-/-^* mice have been described previously [10,11]. Littermate male *Me1-/-* and wild-type mice were challenged with a high fat diet (HFD) RDI D12492 (New Brunswick, NJ, 44.9% kcal from fat; 35.1% kcal from carbohydrate; 20.0% kcal from protein; 4.73 kcal/g) starting at 7-8 weeks of age for 19 weeks. Oral glucose tolerance test (OGTT) was performed at week 23-24 of age and terminal blood serum was collected at week 26-27 of age. For females, HFD was initiated at week 8-10 and continued for 19 weeks. OGTT was performed at week 26-28 of age and terminal serum samples were collected at week 27-28 of age. Mice were euthanized at the end of HFD period. For OGTT, glucose was administered at 2g/kg of mouse mass via oral gavage, mice were fasted 18 hrs. prior and glucose levels were measured using a OneTouch Ultra glucometer (LifeScan, Inc, Milpitas, CA ) at 0, 30, 60, 90, and 120 min. Serum was collected from blood using Becton Dickson (Franklin Lakes, NJ ) Microtainer tubes with SST. Insulin and leptin were measured using Millipore's (Billerica, MA) Multiplexed Biomarker Immunoassay for Luminex xMap using a Bio-Rad's (Hercules, CA) Bio-Plex machine. The other serum parameters were measured using a colorimetric assay. Triglycerides was measured at OD 510 nm using reagents from Roche Diagnostics (Indianapolis, IN). Cholesterol was measured using reagents from Stanbio (Boerne, TX) at OD 510 nm as well.

**Identification and pathway analysis of the *Me1*-/- knockout gene expression signature**

The gonadal white adipose tissues were collected from 10 male *Me*1-/- mice and 10 male littermate wild-type (wt) control mice. The adipose tissues were homogenized and total RNA extracted using Trizol reagent (Invitrogen, CA) according to the manufacturer’s instructions. Three micrograms of total RNA was reverse transcribed and labeled with either Cy3 or Cy5 fluorochrome. Labeled complementary RNA (cRNA) from each animal was hybridized against a pool of labeled cRNAs constructed from equal aliquots of RNA from the control animals using Agilent arrays consisting of 39,556 non-control probes that represent 37,687 genes. All hybridizations were performed in fluor reversal for 24 hours in a hybridization chamber, washed, and scanned using a confocal laser scanner. Arrays were quantified on the basis of spot intensity relative to background, adjusted for experimental variation between arrays using average intensity over multiple channels, and fitted to a previously described error model to determine significance [12,13]. Gene expression measures are reported as the ratio of the mean log10 intensity (mlratio).

We selected the “most transcriptionally active genes” using the Resolver program, where these genes represent those with expression levels that vary the most across samples and, thus, are likely more biologically relevant [12-14]. The active genes were defined as those with *P* < 0.05 (as determined by a previously described error model [12-14]) in at least 10% of the animals. A Student’s t-test was used to identify genes with significant differences between *Me1^-/-^* animals and the corresponding wt control mice. These genes were defined as “signature” genes, representing the perturbed gene expression signature as a result of single gene modification. The significance level was set to *P* < 0.05. The false discovery rate at this significance level was calculated using Q-value as reported [15]. The signature gene set identified above was classified using Gene Ontology (GO) [16] and Panther pathway [17] database assignments.

**Discussion of the Role of Malic Enzyme in Diabetes**

*Me1* encodes a cytosolic NADP(+)-dependent enzyme involved in the formation of pyruvate from malate, producing NADPH to supply reducing equivalents for lipogenesis, thus siphoning the reducing equivalents originally derived from glycolysis as NADH to NADPH for fatty acid synthesis [18]. *Me1* is co-regulated together with fatty acid synthetic enzymes by *Chrebp* and *Srebp-1c* and is therefore described as a lipogenic enzyme. Altered levels of Me1 enzyme activity has been associated with obesity in mouse and rat models [19,20], and *Me1*  was identified as a primary candidate gene underlying a porcine QTL associated with backfat thickness [21]. Further, we recently provided direct experimental support for the involvement of *Me1* in obesity-related phenotypic characteristics and in gene networks associated with obesity using a *Me1* knockout (*Me1*-/-) mouse model [22]. Both male and female *Me1*-/- mice on a medium high fat diet and male *Me1*-/- on a high sucrose diet demonstrated decreased body weight, while *Me1*-/- male mice on a high fat diet showed a significant difference in food intake, relative to littermate controls.

As reported, we found *Me1* to be a critical player in this subnetwork, possibly one of the direct genetic drivers of the diabetes phenotypes in the B6×BTBR cross. *Me1* expression levels were found to be very highly regulated in mouse adipose when animals were exposed to either short or long term dietary restrictions [20,23], emphasizing the critical role of *Me1* in energy sensing and metabolism.

Coleman studied the relationship between malic enzyme activity and diabetes susceptibility in leptin receptor-deficient B6 mice vs. the diabetes-susceptible strain C57B6/Ks [24]. He found that the allele of the malic enzyme regulator segregated with plasma glucose in an obese F2 population derived from the B6 and B6/Ks strains. Resistance to diabetes correlated with a higher enzymatic activity of malic enzyme in the liver. Studies in several pairs of mouse strains have suggested that increased lipogenesis in the liver, although a risk factor for hepatic steatosis, correlates with diabetes resistance [25,26]. Obese mice show an increase in the expression of lipogenic enzymes in the liver, although this difference is more dramatic in B6 compared to BTBR. In the B6×BTBR F2 population, both adipose and liver expression of *Me1* was positively correlated with plasma insulin and negatively correlated with plasma glucose, with the high expression allele at the chromosome 9 *cis*-eQTL coming from BTBR (i.e. it is a transgressive locus). These results suggest that high expression of *Me1* in adipose tissue and/or liver might be pro-diabetic. Since *cis*-eQTLs can be highly correlated across tissues, the tissue where its activity is crucial may not be the one in which the eQTL was initially identified. For example, in contrast to its role in lipogenesis in liver and adipose tissue, malic enzyme has been proposed to play a role in glucose-stimulated insulin secretion in beta cells, although this remains controversial [27]. Another possibility is that beta cell proliferation may be modulated by levels of *Me1*. Indeed, Ronnebaum et al. recently reported that siRNA knockdown of *Me1* in 832/13 insulinoma cells resulted in a 26% decrease in ^3^H-thymidine incorporation into DNA.

The fact that loss of *Me1* seems anti-diabetic in lean mice whereas increased Me1 activity seems protective in the obese F2 models is similar to recent studies of stearoyl-Coenzyme A desaturase 1*(Scd1*). Loss of *Scd1* leads to increased insulin sensitivity in lean mice, but promotes diabetes in obese mice, due to loss of beta cell mass. This is likely due to lipotoxicity from excess palmitate [28]. Thus, it is possible that *Me1* plays a role related to insulin sensitivity in adipose tissue and/or liver and a role related to insulin secretion or preservation of beta cell mass in pancreatic beta cells.

**Text S1 References**

**1. Chen Y, Zhu J, Lum PY, Yang X, Pinto S, et al. (2008) Variations in DNA elucidate molecular networks that cause disease. Nature 452: 429-435.**

**2. Hughes TR, Mao M, Jones AR, Burchard J, Marton MJ, et al. (2001) Expression profiling using microarrays fabricated by an ink-jet oligonucleotide synthesizer. Nat Biotechnol 19: 342-347.**

**3. Lasky-Su J, Lyon HN, Emilsson V, Heid IM, Molony C, et al. (2008) On the replication of genetic associations: timing can be everything! Am J Hum Genet 82: 849-858.**

**4. Schadt EE, Molony C, Chudin E, Hao K, Yang X, et al. (2008) Mapping the genetic architecture of gene expression in human liver. PLoS Biol 6: e107.**

**5. Stoehr JP, Nadler ST, Schueler KL, Rabaglia ME, Yandell BS, et al. (2000) Genetic obesity unmasks nonlinear interactions between murine type 2 diabetes susceptibility loci. Diabetes 49: 1946-1954.**

**6. Keller MP, Choi Y, Wang P, Davis DB, Rabaglia ME, et al. (2008) A gene expression network model of type 2 diabetes links cell cycle regulation in islets with diabetes susceptibility. Genome Res 18: 706-716.**

**7. Frazer KA, Ballinger DG, Cox DR, Hinds DA, Stuve LL, et al. (2007) A second generation human haplotype map of over 3.1 million SNPs. Nature 449: 851-U853.**

**8. Ravasz E, Somera AL, Mongru DA, Oltvai ZN, Barabasi AL (2002) Hierarchical organization of modularity in metabolic networks. Science 297: 1551-1555.**

**9. Lee CY, Lee SM, Lewis S, Johnson FM (1980) Identification and biochemical analysis of mouse mutants deficient in cytoplasmic malic enzyme. Biochemistry 19: 5098-5103.**

**10. Chen L, Zhang L, Zhao Y, Xu L, Shang Y, et al. (2009) Prioritizing risk pathways: a novel association approach to searching for disease pathways fusing SNPs and pathways. Bioinformatics 25: 237-242.**

**11. Cai XJ, Yang J, Yu H, Liang X, Wang YF, et al. (2009) [Laparoscopic radical resection for gallbladder carcinoma]. Zhonghua Yi Xue Za Zhi 89: 1278-1280.**

**12. Weng L, Dai H, Zhan Y, He Y, Stepaniants SB, et al. (2006) Rosetta error model for gene expression analysis. Bioinformatics 22: 1111-1121.**

**13. Bouton CM, Pevsner J (2003) DRAGON and DRAGON view: information annotation and visualization tools for large-scale expression data. Curr Protoc Bioinformatics Chapter 7: Unit 7 4.**

**14. He YD, Dai H, Schadt EE, Cavet G, Edwards SW, et al. (2003) Microarray standard data set and figures of merit for comparing data processing methods and experiment designs. Bioinformatics 19: 956-965.**

**15. Storey JD, Tibshirani R (2003) Statistical significance for genomewide studies. Proc Natl Acad Sci U S A 100: 9440-9445.**

**16. Ashburner M, Ball CA, Blake JA, Botstein D, Butler H, et al. (2000) Gene ontology: tool for the unification of biology. The Gene Ontology Consortium. Nat Genet 25: 25-29.**

**17. Altermann E, Klaenhammer TR (2005) PathwayVoyager: pathway mapping using the Kyoto Encyclopedia of Genes and Genomes (KEGG) database. BMC Genomics 6: 60.**

**18. MacDonald MJ (1995) Feasibility of a mitochondrial pyruvate malate shuttle in pancreatic islets. Further implication of cytosolic NADPH in insulin secretion. J Biol Chem 270: 20051-20058.**

**19. Qian S, Mumick S, Nizner P, Tota MR, Menetski J, et al. (2008) Deficiency in cytosolic malic enzyme does not increase acetaminophen-induced hepato-toxicity. Basic Clin Pharmacol Toxicol 103: 36-42.**

**20. van Schothorst EM, Keijer J, Pennings JL, Opperhuizen A, van den Brom CE, et al. (2006) Adipose gene expression response of lean and obese mice to short-term dietary restriction. Obesity (Silver Spring) 14: 974-979.**

**21. Vidal O, Varona L, Oliver MA, Noguera JL, Sanchez A, et al. (2006) Malic enzyme 1 genotype is associated with backfat thickness and meat quality traits in pigs. Anim Genet 37: 28-32.**

**22. Yang X, Deignan JL, Qi H, Zhu J, Qian S, et al. (2009) Validation of candidate causal genes for obesity that affect shared metabolic pathways and networks. Nat Genet.**

**23. Higami Y, Pugh TD, Page GP, Allison DB, Prolla TA, et al. (2004) Adipose tissue energy metabolism: altered gene expression profile of mice subjected to long-term caloric restriction. Faseb J 18: 415-417.**

**24. Coleman DL (1992) The influence of genetic background on the expression of mutations at the diabetes (db) locus in the mouse. VI: Hepatic malic enzyme activity is associated with diabetes severity. Metabolism 41: 1134-1136.**

**25. Colombo C, Haluzik M, Cutson JJ, Dietz KR, Marcus-Samuels B, et al. (2003) Opposite effects of background genotype on muscle and liver insulin sensitivity of lipoatrophic mice. Role of triglyceride clearance. J Biol Chem 278: 3992-3999.**

**26. Lan H, Rabaglia ME, Stoehr JP, Nadler ST, Schueler KL, et al. (2003) Gene expression profiles of nondiabetic and diabetic obese mice suggest a role of hepatic lipogenic capacity in diabetes susceptibility. Diabetes 52: 688-700.**

**27. Jensen MV, Joseph JW, Ronnebaum SM, Burgess SC, Sherry AD, et al. (2008) Metabolic cycling in control of glucose-stimulated insulin secretion. Am J Physiol Endocrinol Metab 295: E1287-1297.**

**28. Flowers JB, Rabaglia ME, Schueler KL, Flowers MT, Lan H, et al. (2007) Loss of stearoyl-CoA desaturase-1 improves insulin sensitivity in lean mice but worsens diabetes in leptin-deficient obese mice. Diabetes 56: 1228-1239.**
